# Saturated fatty acid biomarkers and risk of cardiometabolic diseases: A meta-analysis of prospective studies

**DOI:** 10.3389/fnut.2022.963471

**Published:** 2022-08-15

**Authors:** Zhaoqing Li, Haoyuan Lei, Hong Jiang, Yahui Fan, Jia Shi, Chao Li, Fangyao Chen, Baibing Mi, Mao Ma, Jing Lin, Le Ma

**Affiliations:** ^1^School of Public Health, Xi’an Jiaotong University Health Science Center, Xi’an, China; ^2^The First Affiliated Hospital, Xi’an Jiaotong University Health Science Center, Xi’an, China; ^3^Key Laboratory of Environment and Genes Related to Diseases (Xi’an Jiaotong University), Ministry of Education of China, Xi’an, China

**Keywords:** saturated fatty acid biomarker, cardiometabolic disease, type 2 diabetes, coronary heart disease, stroke, cardiovascular disease, meta-analysis

## Abstract

**Background and aims:**

Evidence regarding associations of circulating saturated fatty acids (SFAs) with chronic diseases is mixed. The objective of this study was to determine the associations between total or individual SFA biomarkers and the risk of cardiometabolic diseases.

**Methods:**

Four electronic databases were searched from inception to March 2022. Three investigators independently assessed for inclusion and extracted data. Random-effects or fixed-effects models was used to estimate the pooled relative risks (RRs) and corresponding 95% confidence intervals (CIs) for the association of total or individual SFA biomarkers, including even-chain SFAs (e.g., 14:0, myristic acid; 16:0, palmitic acid; 18:0, stearic acid), odd-chain SFAs (e.g., 15:0, pentadecanoic acid; 17:0, margaric acid) and very-long-chain SFAs (VLCSFAs; e.g., 20:0, arachidic acid; 22:0, behenic acid; 24:0, lignoceric acid), with risk of incident type 2 diabetes (T2D), cardiovascular disease [CVD; coronary heart disease (CHD) inclusive of stroke], CHD and stroke.

**Results:**

A total of 49 prospective studies reported in 45 articles were included. Higher concentration of circulating total SFAs was associated with an increasing risk of cardiometabolic diseases, the risk increased significantly by 50% for CVD (95%CI:1.31–1.71), 63% for CHD (95%CI:1.38–1.94), 38% for stroke (95%CI:1.05–1.82), respectively. Similarly, levels of even-chain SFAs were positively associated with higher risk of chronic diseases, with RRs ranging from 1.15 to 1.43. In contrast, the risk of cardiometabolic diseases was reduced with increasing odd-chain SFA levels, with RRs ranging from 0.62 to 0.91. A higher level of VLCSFAs corresponded to 19% reduction in CVD. Further dose-response analysis indicated that each 50% increment in percentage of total SFAs in circulating was associated with an 8% higher risk of T2D (RR: 1.08, 95%CI: 1.02–1.14) and trends toward higher risk of CVD (RR: 1.15, 95%CI: 0.98–1.34). Inverse linear relationships were observed between 17:0 biomarker and T2D or CVD risk.

**Conclusion:**

Our findings support the current recommendations of reducing intake of saturated fat as part of healthy dietary patterns. Further studies are needed to confirm our findings on these SFAs in relation to cardiometabolic outcomes and to elucidate underlying mechanisms.

**Systematic review registration:**

[https://www.crd.york.ac.uk/prospero/display_record.php?ID=CRD42022329182], identifier [CRD42022329182].

## Introduction

Limiting intake of saturated fatty acids (SFAs) has been considered as a key component to reduce the risk of chronic disorders for decades (1). The 2020 Dietary Guidelines for Americans recommended that SFAs comprise below 10% of total daily energy intake starting at age 2 years (2). The American Heart Association/American College of Cardiology guidelines have also set a target to decrease SFA intake to 5–6% of calories (3). *In vitro* and animal studies have demonstrated that SFAs can accelerate the atherosclerotic process, induce cellular inflammation and influence the insulin sensitivity (4, 5). Results from a meta-analysis of randomized controlled trials conducted by the American Heart Association demonstrated that replacing SFAs with unsaturated fats, especially polyunsaturated fats, will lower the incidence of cardiovascular disease (CVD) by approximately 30% (6). Therefore, it is important to accurately evaluate the effects of SFAs on health for the control of cardiometabolic diseases.

Associations of dietary SFAs with the incidence of major cardiometabolic diseases have been reported by some large prospective cohort studies, and findings of these studies were inconsistent (7–9). Interpretation has been complicated by the imprecision of dietary questionnaires, measurement errors, different study designs, variation in population characteristics, as well as bioavailability of these fatty acids (10–12). To reduce these limitations, biomarkers which are free of misclassification, reporting bias and other measurement errors can be used to elucidate associations between the intake of SFAs and disease risk (13, 14). An increasing number of prospective studies have been performed to investigate the associations between circulating total SFAs and risk of metabolic and cardiovascular event; however, the results were conflicting (15–17). In addition, previous studies mostly focused on the coronary heart disease (CHD), evidence regarding associations of circulating SFA with other cardiometabolic disease [such as type 2 diabetes (T2D) and stroke] is still needed to be explored. Furthermore, increasing evidence suggested that individual SFA tend to have different biological functions (18). Previous meta-analyses in 2014 reported that even-chain SFAs were positively associated with coronary risk (19); however, null or inverse association was observed for other subtypes of SFAs such as odd-chain SFAs and very-long-chain SFAs (VLCSFAs) in recent studies (20, 21). Therefore, the impact of individual SFA on cardiometabolic disease needs to be further studied and established.

To fill this knowledge gap, a dose-response meta-analysis of data from prospective studies was conducted to evaluate the associations between the circulating even-chain SFAs, odd-chain SFAs, VLCSFAs as well as the total SFAs and the incident of T2D, CVD, CHD, and stroke.

## Materials and methods

This study was registered in PROSPERO (CRD42022329182) and reported in accordance with the Preferred Reporting Items for Systematic Review and Meta-Analysis (PRISMA) guidelines.

## Search strategy

A comprehensive literature search of Web of Science, PubMed, EMBASE, and Cochrane Library were conducted to identify relevant published articles from inception to March 2022. The following terms were used: “fatty acids” or “saturated fatty acid” or “saturated fatty acids” or “lauric acid” or “myristic acid” or “pentadecanoic acid” or “palmitic acid” or “margaric acid” or “stearic acid” or “arachidic acid” or “heneicosylic acid” or “behenic acid” or “tricosylic acid” or “lignoceric acid,” AND “type 2 diabetes” or “diabetes mellitus” or “impaired glucose” or “impaired fasting insulin” or “cardiovascular disease” or “coronary heart disease” or “heart disease” or “ischemic heart disease” or “coronary artery disease” or “myocardial infarction” or “stroke” or “ischemic stroke” or “haemorrhagic stroke,” AND “blood” or “marker” or “biomarker” or “serum” or “plasma” or “whole blood” or “adipose tissue” or “circulating” or “erythrocytes” or “red blood cell” or “cholesterol esters,” AND “prospective” or “nested case-control” or “cohort” or “case-cohort” or “follow-up” or “longitudinal.” The search was restricted to human studies without any language restriction. Additionally, reference lists of retrieved articles, review articles, and meta-analyses were manually scanned to identify any other relevant studies. Corresponding authors of eligible articles were contacted for any further relevant work, published or unpublished, to reduce risk of publication bias.

## Selection criteria

Studies were included in the meta-analysis if they satisfied the following criteria: (1) the study design was prospective (cohort, nested case-control, and case-cohort) with a follow-up more than 1 year; (2) the exposures of interest were total or individual SFA biomarkers [even-chain SFAs (e.g., 14:0, myristic acid; 16:0, palmitic acid; 18:0, stearic acid), odd-chain SFAs (e.g., 15:0, pentadecanoic acid; 17:0, margaric acid) and VLCSFAs (e.g., 20:0, arachidic acid; 22:0, behenic acid; 24:0, lignoceric acid)] measured in any type of tissue [circulating blood (whole blood, serum, plasma, and erythrocyte fraction) or adipose tissue]; (3) the endpoints of interest included incident T2D, CVD, CHD, and stroke; (4) the adjusted relative risks (RRs), odds ratios, or hazard ratios with corresponding 95% confidence intervals (CIs) or standard errors were presented or could be calculated. Whenever reports referred to identical outcomes from the same population, only those with the highest number of cases or the longest follow-up times were retained to avoid data duplication. Potentially eligible studies were assessed independently by three investigators (ZQL, HYL, and HJ), with discrepancies resolved by discussion until consensus was reached.

## Data extraction and quality assessment

Data extraction were conducted independently by 3 investigators (ZQL, HYL, and HJ). A standardized data collection form was applied to extract the following baseline characteristics from each study: first author name, publication year, study location, study name, study design, follow-up duration, participant characteristics (age, proportion of men, and number of participants), SFA subtype, biological sample type (plasma, serum, erythrocyte membrane, and adipose tissue), assessment method [gas chromatography (GC), gas-liquid chromatography (GLC), and nuclear magnetic resonance-based profiling (NMR)], outcomes of interest (type and number of cases), covariates adjusted for in the multivariable model, and the risk estimate with 95%CIs for all categories of each biomarkers. When studies reported multiple results based on different numbers of covariates included in statistical adjustments, the results that adjusted for the most number of variables were extracted.

Study quality was performed by the same authors according to the validated Newcastle-Ottawa Scale (NOS) (22), which awards 0–9 points and incorporates information on selection (range 0–4 points), comparability (range 0–2 points), and outcome assessment (range 0–3 points). We considered NOS scores of 0–3, 4–6, and 7–9 as low, medium, and high quality, respectively. Any discrepancy in data extraction or quality assessments between investigators were discussed or resolved by a senior author (LM) until a joint consensus was reached.

### Data synthesis and statistical analysis

In this meta-analysis, the RRs were used as the common measures of associations across studies, and odds ratios and hazard ratios were considered approximations of RRs. As different studies might report different exposure categories, methods previously described were used to derive estimates of associations corresponding to the comparison between the top and bottom third of SFAs distributions for the meta-analysis (19). The strategy was to harmonize different comparison groups used in individual studies, such as quartiles, quintiles, or other categorizations, or per standard deviation (SD) change. In brief, for studies that provided RRs per SD change of SFAs, we applied a factor of 2.18 to the log RR to derive the RRs comparing extreme thirds, assuming a normal distribution. Similarly, the factor of 2.54 or 2.80 was applied to convert estimates for comparing extreme quartiles or quintiles, respectively. The standard error of the transformed log RRs was calculated after applying the same factors (23). When studies used multiple measures as biomarker, the overall risk estimate was based on different duration of intake reflection according to the following list: adipose tissue, erythrocyte phospholipids, plasma phospholipids, total plasma or serum, and cholesterol esters. Studies that separately reported results by sex without presenting overall estimates were pooled to derive a single effect size for the study. Assessment of heterogeneity between the studies was based on Q test and *I*^2^ statistic (24). A Cochran’s Q *P* < 0.10 and *I*^2^ value > 50% was considered to indicate significant heterogeneity and the random-effects model was used. Otherwise, fixed-effects model was performed. Meta-regression was conducted to examine sources of heterogeneity and the influence of potential residual confounding factors, such as study design [prospective cohort study (PC), prospective case-cohort study (PCC), or nested case-control study (NCC)], geographical location (the United States, Europe, or Asia), assessment method (GC, GLC, or NMR), biomarker type (plasma, serum, erythrocyte membrane, or adipose tissue), number of cases (< 300 or ≥ 300), as well as study quality (moderate or high). Dose-response analyses were conducted based on the method proposed by Greenland and Longnecker (25), studies that reported RRs with 95%CIs for at least three exposure categories were included. When studies reported only the total number of cases or total person-years and the exposure was defined in quantile, the distribution of cases or person-years was calculated by dividing the total number by the number of quantiles. For the studies that did not present the median or mean doses of SFAs, we chose the midpoint of each category as the alternative. When the highest category did not have an upper bound, the length of the open-ended interval was assumed to be the same as that of the adjacent interval. When the lowest category was open-ended, the lower bound was set to zero. Sensitivity analyses were conducted by omitting one study in each turn to assess the impact of individual studies on the overall estimated risk. Potential publication bias was explored by using visual appreciation of funnel plots, Begg’s rank correlation and Egger’s weighted regression tests. The influence of a potential publication bias on findings was explored by using the Duval and Tweedie trim and fill procedure (26). All analyses were performed using Stata, version 12.0 (Stata Corporation, College Station, TX, United States). All *P*-values reported are two-sided and a *P*-value < 0.05 was considered statistically significant, except where otherwise specified.

## Results

### Studies retrieved and characteristics

**FIGURE 1:**
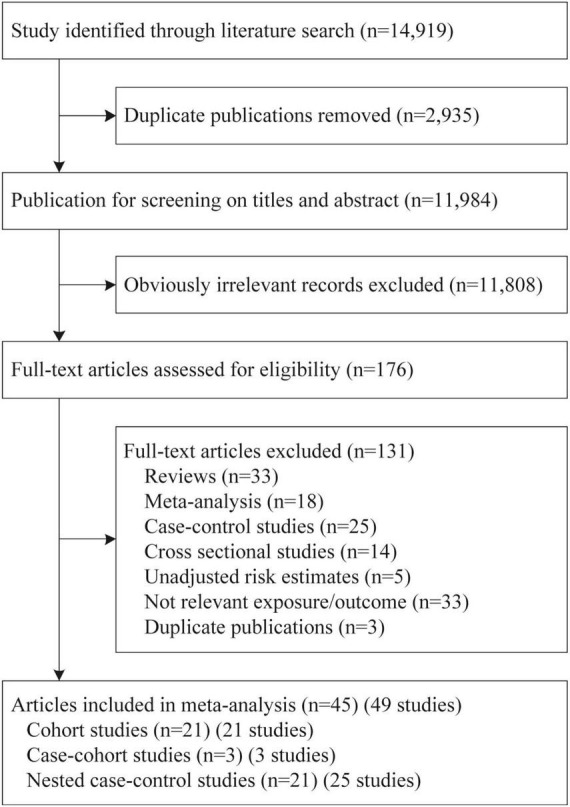
Flow chart of study selection.

A total of 14,919 potentially relevant citations were identified by search strategy, of which 2,935 duplicates were initially excluded. After screening titles and abstracts, 11,808 records were further removed, leaving 176 articles retrieved for full-text review. Finally, a total of 49 studies reported in 45 articles were included for the present meta-analysis (Figure 1) (15–17, 20, 21, 27–66).

The main characteristics of the included prospective studies are listed in Supplementary Table 1. The 49 identified studies comprised of 21 prospective cohort studies, three case-cohort studies, and 25 nested case-control studies. The number of participants in each included study varied from 188 to 95,854, with a total of 220,590 participants and 46,355 cases (21,252 T2D cases, 14,613 CVD cases, 7,314 CHD cases, and 3,176 stroke cases). 24 studies were conducted in the United States, 15 in Europe, and 10 in Asia. The duration of follow-up in prospective studies ranged from 1.5 to 30.7 years. The average age of participants ranged from 48.9 to 79.2 years. 32 studies comprised both men and women as participants, nine studies included men only and eight included women only. Concentrations of circulating SFAs were quantified by GC (*n* = 28), GLC (*n* = 18), NMR (*n* = 2), and GC-tandem mass spectrometry (*n* = 1). 48 studies measured SFA biomarkers in circulating blood (21 in plasma, 15 in erythrocyte membrane, and 12 in serum) and one in adipose tissue. Most studies were adjusted for age (*n* = 47) and smoking (*n* = 46), and alcohol drinking (*n* = 40), physical activity (*n* = 39) and body mass index (*n* = 38) were also controlled for in many studies. The results of the study quality assessment showed that 44 studies were graded as high quality, others were graded as moderate quality (Supplementary Table 2).

### Association between saturated fatty acid biomarkers and type 2 diabetes

**FIGURE 2:**
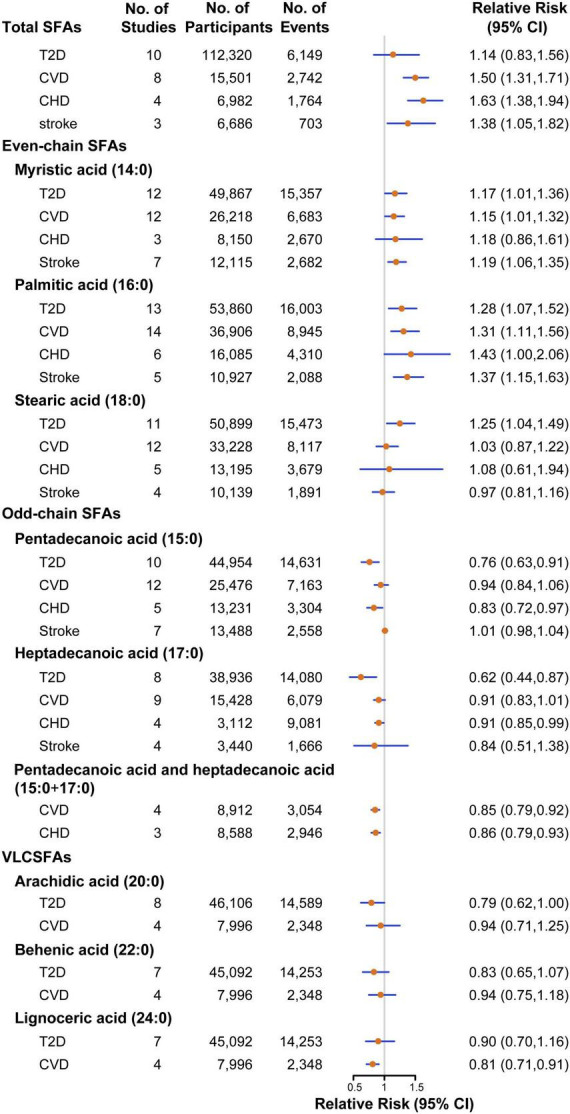
Pooled relative risks of T2D, CVD, CHD, stroke comparing the highest with the lowest tertile of SFA biomarkers. CHD, coronary heart disease; CI, confidence interval; CVD, cardiovascular disease; SFAs, saturated fatty acids; T2D, type 2 diabetes; VLCSFAs, very-long-chain SFAs.

Twenty-two studies were assessed to estimate risk between the concentrations of SFA biomarkers and T2D, including 166,745 participants and 21,252 T2D events (17, 20, 27–44). The pooled estimate indicated that a higher level of total SFAs was associated with a non-significant higher risk of T2D (RR: 1.14, 95%CI: 0.83–1.56, *P* = 0.43; *P*_*heterogeneity*_ < 0.001; Figure 2 and Supplementary Figure 1). For even-chain SFAs, compared with participants in the lowest tertile, those in the highest tertile of 16:0 level had a 28% significantly higher T2D risk (RR: 1.28, 95%CI: 1.07–1.52, *P* = 0.01; *P*_*heterogeneity*_ < 0.001; Supplementary Figure 2). Similar association was also found between other two even-chain SFAs and CVD risk, with the RR of 1.17 (95%CI: 1.01–1.36, *P* = 0.03; *P*_*heterogeneity*_ = 0.004; Supplementary Figure 3) for 14:0 and 1.25 (95%CI: 1.04–1.49, *P* = 0.01; *P*_*heterogeneity*_ < 0.001; Supplementary Figure 4) for 18:0, respectively. By contrast, when comparing the extreme tertiles of odd-chain SFA level, the risk in T2D decreased significantly by 24% for 15:0 (RR: 0.76, 95%CI: 0.63–0.91, *P* = 0.003; *P*_*heterogeneity*_ = 0.002; Supplementary Figure 5), 38% for 17:0 (RR: 0.62, 95%CI: 0.44–0.87, *P* = 0.01; *P*_*heterogeneity*_ < 0.001; Supplementary Figure 6). Higher VLCSFAs status was associated with a non-significant lower risk of T2D [RR was 0.79 for 20:0 (95%CI: 0.62–1.00, *P* = 0.06; *P*_*heterogeneity*_ < 0.001; Supplementary Figure 7), 0.83 for 22:0 (95%CI: 0.65–1.07, *P* = 0.16; *P*_*heterogeneity*_ < 0.001; Supplementary Figure 8), 0.90 for 24:0 (95%CI: 0.70–1.16, *P* = 0.42; *P*_*heterogeneity*_ = 0.001; Supplementary Figure 9)]. The association between circulating SFAs and T2D risk was not significantly modified by study type, sex, geographical location, number of cases, assessment method, biomarker type and study quality (Supplementary Figures 10–12). The result of dose-response analysis found that each 50% increment in percentage of total SFAs in circulating corresponded to an 8% (RR: 1.08, 95%CI: 1.02–1.14, *P* = 0.01; *P*_*linearity*_ = 0.84) higher risk of T2D. For every 50% increase in percentage of odd-chain SFAs concentration in total SFAs, the risk of T2D was reduced by 8% for 15:0 (RR: 0.92, 95%CI: 0.83–1.02, *P* = 0.11; *P*_*linearity*_ = 0.25), 16% for 17:0 (RR: 0.84, 95%CI: 0.76–0.92, *P* < 0.001; *P*_*linearity*_ = 0.12), respectively.

### Association between saturated fatty acid biomarkers and cardiovascular disease

Association between circulating SFAs and risk of CVD were investigated in 27 studies, with 53,845 participants and 14,613 CVD events (15, 16, 21, 45–66). Participants in the highest category of circulating total SFAs had a 50% higher risk with CVD compared with those in the lowest category (RR: 1.50, 95%CI: 1.31–1.71, *P* < 0.001; *P*_heterogeneity_ = 0.56; Supplementary Figure 13). For even-chain SFAs, the pooled results indicated that higher 16:0 status was associated with a 31% increased risk of CVD (RR: 1.31, 95%CI: 1.11–1.56, *P* = 0.002; *P*_heterogeneity_ < 0.001; Supplementary Figure 14). Similarly, higher levels of 14:0 was also associated with a 15% higher risk of CVD (RR: 1.15, 95%CI: 1.01–1.32, *P* = 0.03; *P*_heterogeneity_ < 0.001; Supplementary Figure 15). For odd-chain SFAs, higher levels of the sum of 15:0 and 17:0 had a 15% lower risk of CVD (RR: 0.85, 95%CI: 0.79–0.92, *P* < 0.001; *P*_heterogeneity_ = 0.15; Supplementary Figure 16). For VLCSFAs, in comparison with the lowest category, the highest category of 24:0 was associated with a 19% (RR: 0.81, 95%CI: 0.71–0.91, *P* = 0.001; *P*_heterogeneity_ = 0.45; Supplementary Figure 17) reduced risk of CVD. 15:0, 17:0, 18:0, 20:0, and 22:0 biomarkers were not significantly associated with incidence of CVD (Supplementary Figures 18–22). In stratified analyses, the association between 16:0 status and CVD risk was significantly stronger among NCC studies (RR: 1.86, 95%CI: 1.32–2.62) compared with PC studies (Supplementary Figures 10–12). In the dose-response analysis, a non-significant linear association was noted between total SFAs biomarker and CVD risk, with the RR of 1.15 (95%CI: 0.98–1.34, *P* = 0.09; *P*_linearity_ = 0.96) for each 50% increment in percentage of total SFAs in circulating. For every 50% increment in percentage of 17:0 and 24:0 concentrations of total SFAs, the RR of CVD decreased by 18% for 17:0 (RR:0.82, 95%CI: 0.70–0.97, *P* = 0.02; *P*_linearity_ = 0.84), 10% for 24:0 (RR:0.90, 95%CI: 0.82–0.98, *P* = 0.02; *P*_linearity_ = 0.14), respectively.

### Association between saturated fatty acid biomarkers and coronary heart disease

A total of 14 studies with 7,314 cases in 26,772 participants were included to investigate the relationship between circulating SFAs and CHD risk (15, 16, 49–59). The overall effect estimates of CHD comparing the top to bottom categories were 1.63 for total SFAs (95%CI: 1.38–1.94, *P* < 0.001; *P*_heterogeneity_ = 0.45; Supplementary Figure 23). For even-chain SFAs, the pooled result indicated that a higher level of 16:0 was associated with a higher risk of CHD (RR: 1.43, 95%CI: 1.00–2.06, *P* = 0.05; *P*_heterogeneity_ = 0.003; Supplementary Figure 24). The levels of odd-chain SFAs was associated with a significantly lower CHD risk by 17% for 15:0 (RR: 0.83, 95%CI: 0.72–0.97, *P* = 0.02; *P*_heterogeneity_ = 0.21; Supplementary Figure 25), 9% for 17:0 (RR: 0.91, 95%CI: 0.85–0.99, *P* = 0.03; *P*_heterogeneity_ = 0.22; Supplementary Figure 26) and 14% for the sum of 15:0 and 17:0 (RR: 0.86, 95%CI: 0.79–0.93, *P* < 0.001; *P*_heterogeneity_ = 0.23; Supplementary Figure 27), respectively. No significant associations were observed between 14:0, 18:0 concentrations and incidence of CHD (Supplementary Figures 28, 29). In stratified analyses, no evidence of heterogeneity was detected between subgroups. Due to the limited number of included studies, evaluation for dose-response analysis between SFA biomarkers and risk of CHD was not performed.

### Association between saturated fatty acid biomarkers and stroke

The association between circulating SFAs with stroke risk were investigated in nine studies, comprising 16,589 participants and 3,176 stroke events (49, 60–66). The estimated RR of stroke for the comparison of extreme categories was 1.38 for total SFAs (95%CI: 1.05–1.82, *P* = 0.02; *P*_heterogeneity_ = 0.76; Supplementary Figure 30). For even-chain SFAs, both 16:0 and 14:0 biomarkers showed positive associations with stroke risk, with the pooled effect estimates of 1.37 (95%CI: 1.15–1.63, *P* < 0.001; *P*_heterogeneity_ = 0.11; Supplementary Figure 31) and 1.19 (95%CI: 1.06–1.35, *P* = 0.004; *P*_heterogeneity_ = 0.10; Supplementary Figure 32), respectively. No significant associations were detected for circulating 15:0, 17:0 or 18:0 and stroke risk (Supplementary Figures 33–35). No evidence of heterogeneity was detected within any of the subgroups. Dose-response analysis between circulating SFAs and stroke risk was limited by a lack of studies.

### Sensitivity analysis and publication bias

Sensitivity analysis showed that no single study substantially changed the statistical significance or direction of the combined RR for any of the outcomes. Visual inspection of the funnel plot did not reveal substantial asymmetry (Supplementary Figures 36–67). Similarly, the results of the Egger’s tests and Begg’s tests indicated that no evidence of publication bias was found for associations between circulating SFAs and incidence of these cardiometabolic diseases (*P* > 0.05 for both tests).

## Discussion

In the present meta-analysis, higher concentration of circulating total SFAs was associated with an increasing risk of CVD, CHD, and stroke, rather than T2D. Compared with the adverse effect of even-chain SFAs, odd-chain SFAs and VLCSFAs showed a significant inverse association with these cardiometabolic diseases. Our study suggested that individual circulating SFAs may not be equally associated with the risk of chronic diseases, which raises the possibility of generating public health recommendations and nutritional guidelines of SFAs in these cardiometabolic diseases prevention.

Previous studies have found that higher dietary intake of SFAs was associated with an increasing risk of chronic health condition (67). Data from prospective cohort studies also showed that dietary replacement of SFAs with polyunsaturated fat or whole grain carbohydrates might significantly reduce the incidence of cardiometabolic disease (8). Nevertheless, investigations into the effects of dietary SFA consumption on chronic disease in humans have been limited by challenges in accurately assessing saturated fat intake from dietary questionnaires, due to errors in recall and wide variations in SFA content of otherwise similar foods. In this setting, measurements of circulating fatty acids provide a more valid and objective biomarker of dietary fat. Up to date, the associations between circulating saturated fats and the risk of vascular disease still remain controversial. A meta-analysis conducted in 2013 have found essentially null associations between circulating SFAs and coronary risk (19), while a recent matched case-control study among 2,428 postmenopausal women in the Women’s Health Initiative Observational Study showed that 1 mol% increase in plasma phospholipid SFAs was associated with a 20% higher CHD risk (59). Similarly, among 7,354 participants from the European Prospective Investigation into Cancer study who were free of CHD at baseline, the risk of developing CHD almost doubled in participants with highest level of circulating SFAs, when compared with those with lowest level (68). Consistent with these recent findings, the present studies suggested that circulating total SFAs was associated with an increased risk of cardiovascular events. Meanwhile, this result was also supported by subgroups analysis of even-chain SFAs which increment in circulating myristic acid, palmitic acid and stearic acid corresponded to the increased risk of these diseases. As the major components of SFAs, 14:0, 16:0, and 18:0 contribute more than 90% of the fatty acid composition of plasma (69). The strength of the positive associations with cardiometabolic risk were similar for this fatty acid subtype and total SFAs, indicating that the detrimental effects of SFAs may mainly be attributed to even-chain SFAs. The results of the present study also indicated that the positive association with cardiometabolic diseases was more pronounced for high palmitic acid level, as compared with other even-chain SFAs (such as: myristic acid, and stearic acid). In a study of 6,379 postmenopausal women followed up for 10 years, participants in the highest category of circulating palmitic acid level experienced a 24% higher T2D risk compared with those in the lowest category, whereas a weak or no association was found for other even-chain SFAs (39). Similarly, the results from the Cardiovascular Health Study also showed an increased risk of cardiometabolic diseases with increasing circulating concertation of palmitic acid, rather than stearic acid (38). Therefore, our observations, together with evidence from previous studies, indicated that decreasing even-chain SFA level, especially palmitic acid, confers benefits for the prevention of cardiometabolic diseases.

The potential mechanisms through which circulating SFAs especially even-chain SFAs increase the risk of chronic disorders have been proposed, including producing inflammation, inducing lipoprotein disorders, and activating endoplasmic reticulum (ER) stress (70–73). Systemic inflammation is recognized as an important contributor to insulin resistance and atherosclerosis, all of which drives the development of metabolic diseases and CVD (72, 74). In previous *in vitro* experiments, Pal et al. found that even-chain SFAs could induce proinflammatory cytokine expression in adipocytes through the FetA and TLR4 pathway, resulting in insulin resistance (75). Animal experiments have also suggested that SFAs like 16:0 could activate the NLRP3 inflammasome in macrophages by an AMPK-autophagy-ROS signaling pathway (76). Along with the activating of proinflammatory responses, SFAs was thought to impair the LDL-receptor activity and consequently increase the concentration of LDL-cholesterol (LDL-C), an apolipoprotein B-containing lipoprotein that can become trapped in the artery wall and ultimately implicated in the generation of atherosclerosis (77, 78). The adverse effect of palmitic acid on lipoprotein metabolism might explain part of the stronger association between palmitic acid and risk of cardiometabolic diseases. A previous animal study suggested that increasing the absolute amount of dietary 16:0 could increase LDL-cholesterol (LDL-C) more than other even-chain SFAs, through specific modulation of the expression of the LDL receptor and apolipoprotein B genes (79). Clinical trials also showed that serum total cholesterol, apo A-I concentrations and plasma cholesteryl ester transfer protein activity significantly increased in the palmitic acid diet as compared with the stearic acid diet (80, 81). Meanwhile, emerging evidence has suggested that palmitic acid but not myristic or stearic acid exerts adverse effects on ER function by stimulating stress signaling XBP1 and ATF6 (82), which may play a pivotal role in arresting the cell cycle progression in islet (83) and initiating a positive feedback loop in production of even-chain SFAs *via de novo* lipogenesis (84), ultimately contributes to the occurrence of metabolic diseases.

Compared with the deleterious effects of even-chain SFAs, the present results suggested that a higher level of odd-chain SFAs was associated with a lower risk of chronic conditions. Data from Northern Sweden Health and Disease Study suggested that each percent increase of the proportion of this fatty acid species was significantly associated with a risk reduction of a first myocardial infarction (54). The Västerbotten Intervention Programme has also reported that for participants who were free from diabetes at baseline, per SD increase in circulating 15:0 and 17:0 confer relative reductions in T2D incidence of 29 and 46%, respectively (30). The protection against dysregulated lipid metabolism and low-grade inflammation have been proposed to underlie the beneficial role of odd-chain SFAs because the disorder of fat homeostasis and the expression of inflammatory proteins trigger atherosclerosis and other metabolic syndrome (85). In the European Prospective Investigation into Cancer and Nutrition-InterAct study, inverse associations have been observed between plasma phospholipid 15:0 and 17:0 and lipid markers, such as total cholesterol, triglycerides, apolipoprotein A-1, apolipoprotein B. This study also suggested that increasing odd-chain SFAs was accompanied by a decreased C-reactive protein, per 1-*SD* increment of the sum of 15:0 and 17:0 was associated with a 10% decrement of this inflammatory marker (86). Additionally, emerging evidence has indicated that this subclass of fatty acids may provide protection against incident T2D by ameliorating the degree of insulin resistance and β-cell dysfunction. In a multicenter study analyzing different race groups in America without diabetes at baseline, Santaren et al. found that serum concentrations of pentadecanoic acid was positively associated with insulin sensitivity and β cell function (Disposition Index), suggesting that this fatty acid species may play a role in T2D prevention (87). Moreover, as the primary source of odd-chain SFAs, similar association was also found in dairy products (53, 88–93). Two recent meta-analyses of randomized controlled trials reported significant reduction in cardiovascular events with dairy foods intake (94, 95). Despite the possible interactions among these two odd-numbered FAs and multiple bioactive substances in dairy products are largely unknown, the previous studies for dairy consumption still lend support to accumulating evidences of an inverse association between odd-chain SFAs and chronic disease.

For VLCSFAs, the present study of an inverse association between circulating VLCSFAs and CVD risk was in line with the results from previous epidemiological studies (20, 96). While diet is unlikely the main source of these SFAs in the human body (97), the VLCSFAs can be synthesized endogenously (98, 99) and serve as essential components of sphingolipids that is the key constituents of membranes in the human body (100, 101). Previous studies have demonstrated a potential association between sphingolipid metabolism and early markers of the latter chronic disease condition such as adiponectin. In a rat model treated with dietary sphingolipids, a 45-day VLCSFAs supplementation significantly improved adiponectin signaling and consequently increased insulin sensitivity (102). Moreover, as the elongation products of long chain SFAs, this consistent association has also been observed between these fatty acids and plasmalogen (103), an ether lipid which may function as cellular antioxidants and scavenge a variety of reactive oxygen species (104).

Some limitations of this study should be mentioned. Firstly, individual blood SFAs were quantitatively measured once and changes of these SFAs over time might lead to potential random measurement error caused by within-person variation. However, among the Nurses’ Health Study and Nurses’ Health Study II participants, reasonable validity and reproducibility of concentration of SFAs such as 15:0, 16:0, and 24:0 have been demonstrated with intraclass correlation coefficients, indicating a single measurement of fatty acid biomarker levels can reliably represent long-term levels over time (105). Secondly, participants with high SFAs consumption tended to have more unhealthy dietary habits and engage in less physical activity (8) which associated with higher cardiometabolic risk (106). Despite all included studies have adjusted for potential confounding factors, we cannot fully exclude the impact of residual or unmeasured confounding on the observed associations. Because of the prospective design of the current study, misclassification caused by potential confounders such as lifestyle was independent of the outcome ascertainment and was therefore more likely to be non-differential, which would tend to attenuate true associations toward the null. However, the positive association between 16:0 status and CVD risk appeared to be more evident among NCC studies compared with PC studies. This may be attributed to their inherent limitations as the NCC studies are more prone to be influenced by survival bias due to all the cases and controls are enrolled at the same time, which may lead to an overestimation of the magnitude of the association (107). Thirdly, different subtypes of SFAs may have possible additive or synergistic effects that results in the development of cardiometabolic diseases. Further additional studies are warranted to assess the potential interaction between levels of different SFA types and cardiovascular disease. Finally, publication bias should be ascertained. Although no significant publication bias was detected, the potential bias could not be completely ruled out.

## Conclusion

Our meta-analysis of existing prospective studies indicated that a higher concentration of total SFAs and even-chain SFAs was associated with an increasing risk of cardiometabolic diseases, which supported the current recommendations of reducing intake of saturated fat as part of healthy dietary patterns. In addition, our finding of protective effects of odd-chain SFAs has potentially important clinical implications for preventing cardiometabolic diseases. Further studies are apparently needed to confirm our findings on these SFAs in relation to cardiometabolic outcomes and to elucidate underlying mechanisms.

## Data availability statement

The original contributions presented in this study are included in the article/Supplementary material, further inquiries can be directed to the corresponding author/s.

## Author contributions

LM, JL, and MM generated the idea for the study, formulated an analytical plan, and supervised the study. ZL, HL, and HJ searched for relevant studies, conducted quality assessment, and extracted the data. ZL and HJ performed data analysis. ZL and HL wrote the manuscript and all other authors revised the manuscript. All authors approved the final manuscript for submission and acquired, analyzed, or interpreted the data.
