# Supplementary material for: Saturated fatty acid biomarkers and risk of cardiometabolic diseases: A meta-analysis of prospective studies
Source: Front Nutr. 2022 Aug 15;9:963471. doi: 10.3389/fnut.2022.963471 (PMC9421298; doi:10.3389/fnut.2022.963471)
Supplement: Supplementary file 1 [file Data_Sheet_1.pdf]

Figure S1. Pooled relative risk of type 2 diabetes for the highest versus lowest categories of total saturated fatty acid biomarker level.

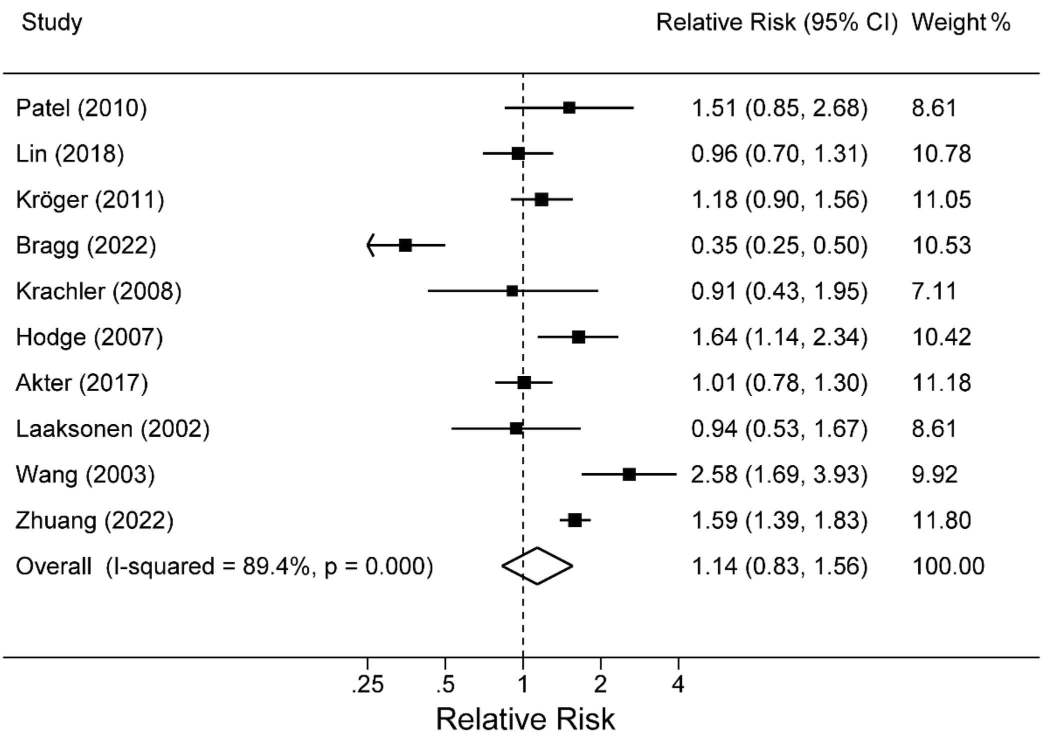

95%CI, 95% confidence interval.

Figure S2. Pooled relative risk of type 2 diabetes for the highest versus lowest categories of palmitic acid (16:0) biomarker level.

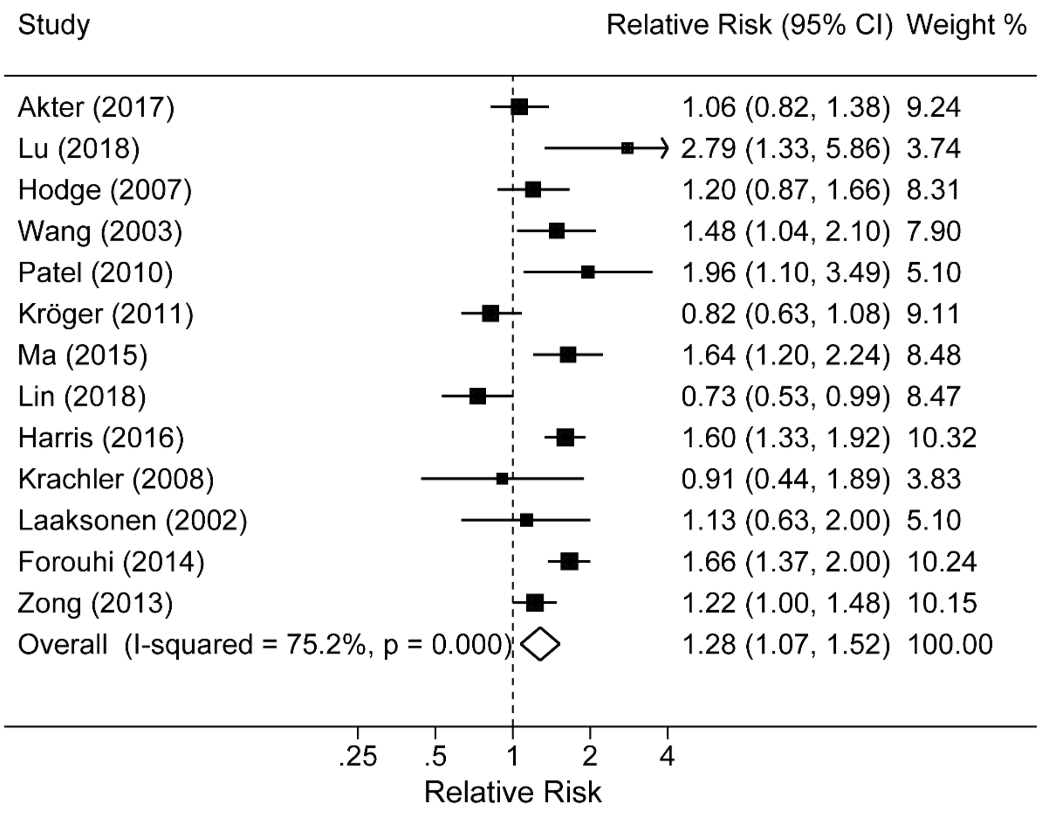

95%CI, 95% confidence interval.

Figure S3. Pooled relative risk of type 2 diabetes for the highest versus lowest categories of myristic acid (14:0) biomarker level.

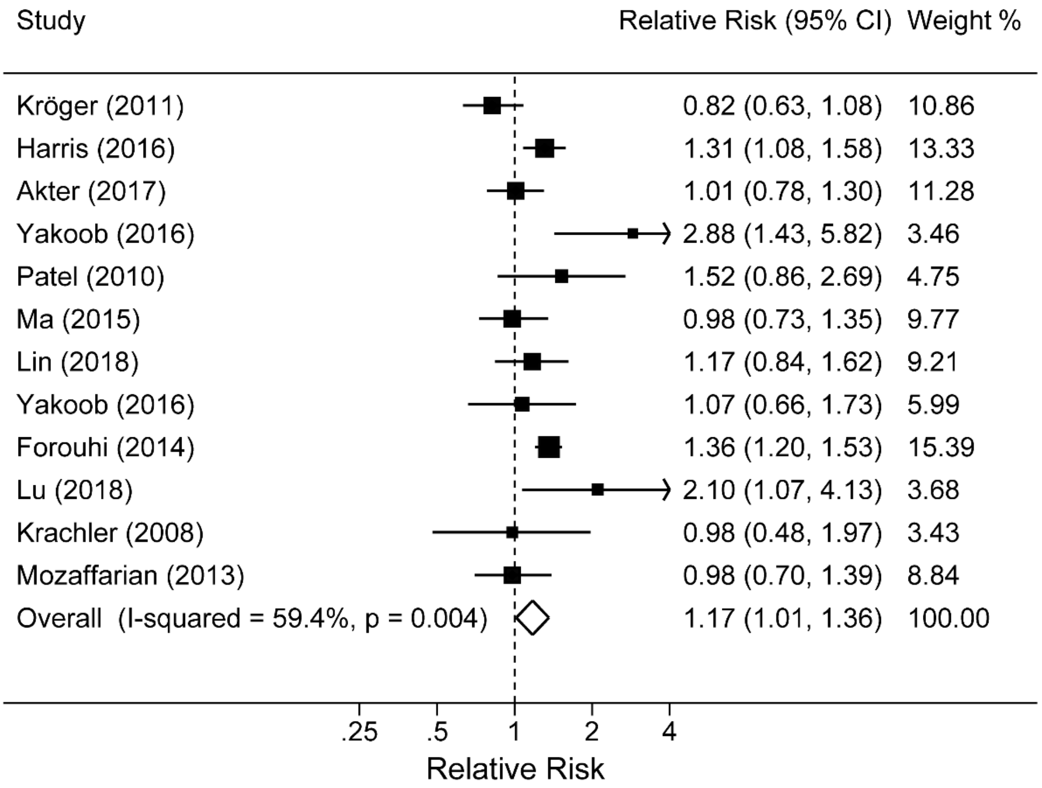

95%CI, 95% confidence interval.

Figure S4. Pooled relative risk of type 2 diabetes for the highest versus lowest categories of stearic acid (18:0) biomarker level.

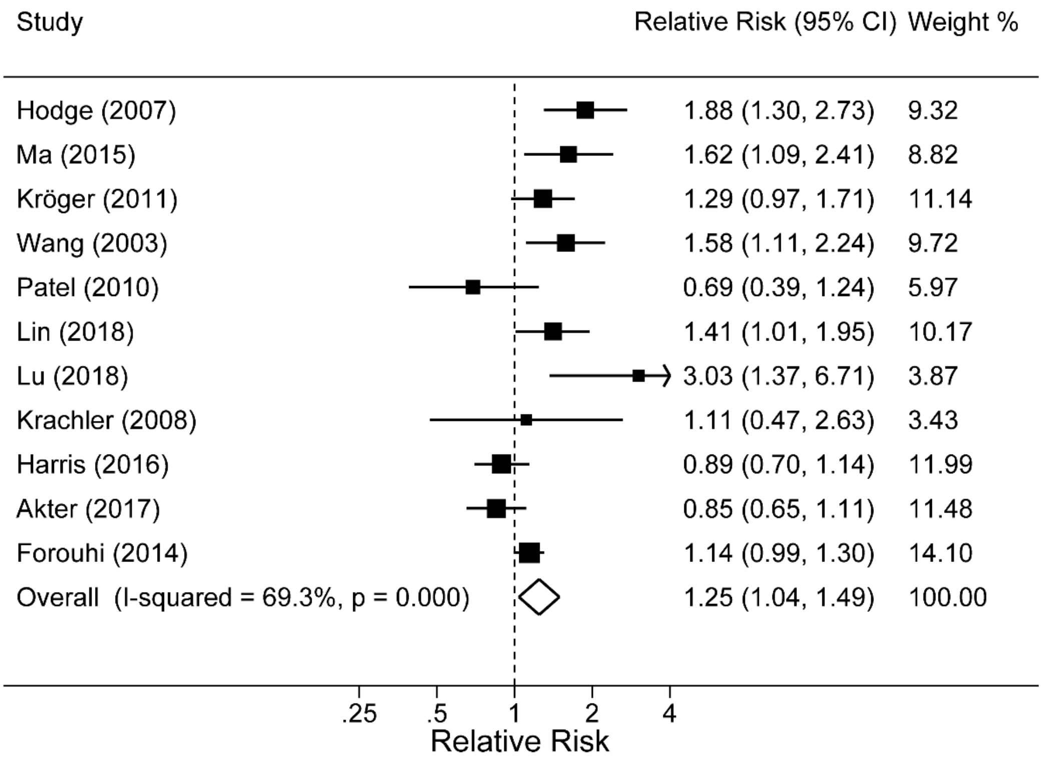

95%CI, 95% confidence interval.

Figure S5. Pooled relative risk of type 2 diabetes for the highest versus lowest categories of pentadecanoic acid (15:0) biomarker level.

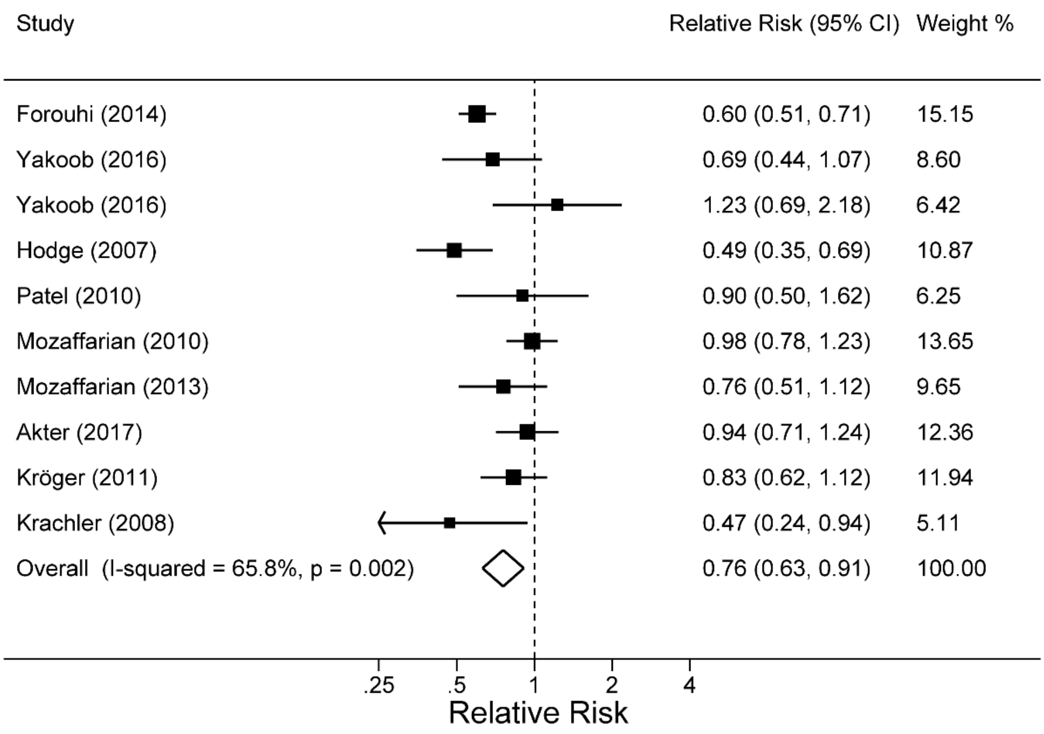

95%CI, 95% confidence interval.

Figure S6. Pooled relative risk of type 2 diabetes for the highest versus lowest categories of margaric acid (17:0) biomarker level.

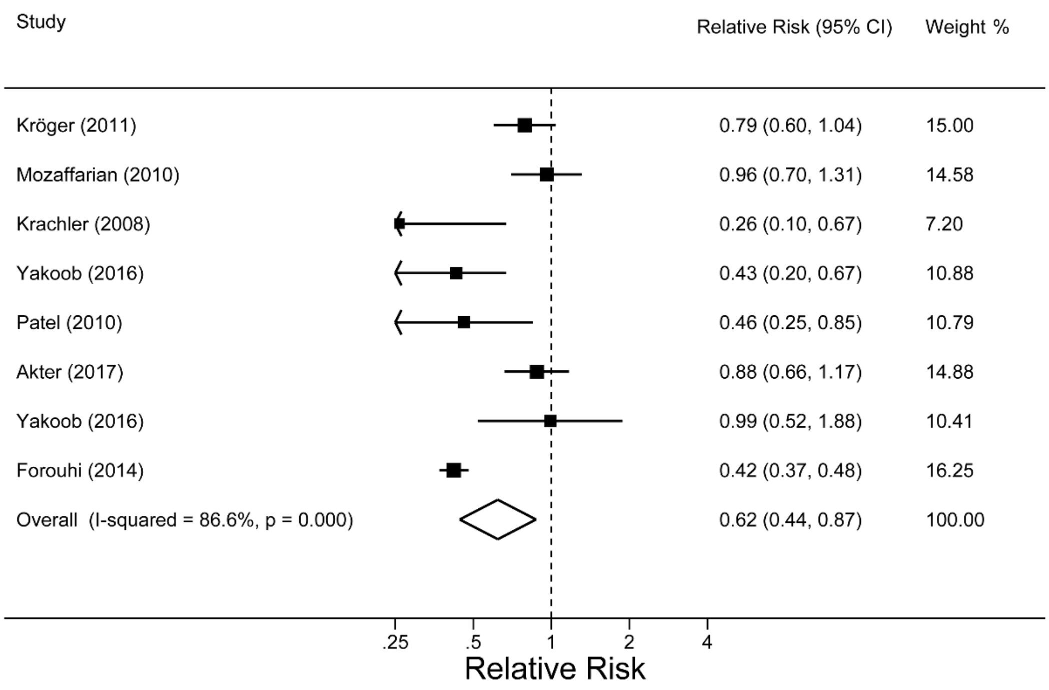

95%CI, 95% confidence interval.

Figure S7. Pooled relative risk of type 2 diabetes for the highest versus lowest categories of arachidic acid (20:0) biomarker level.

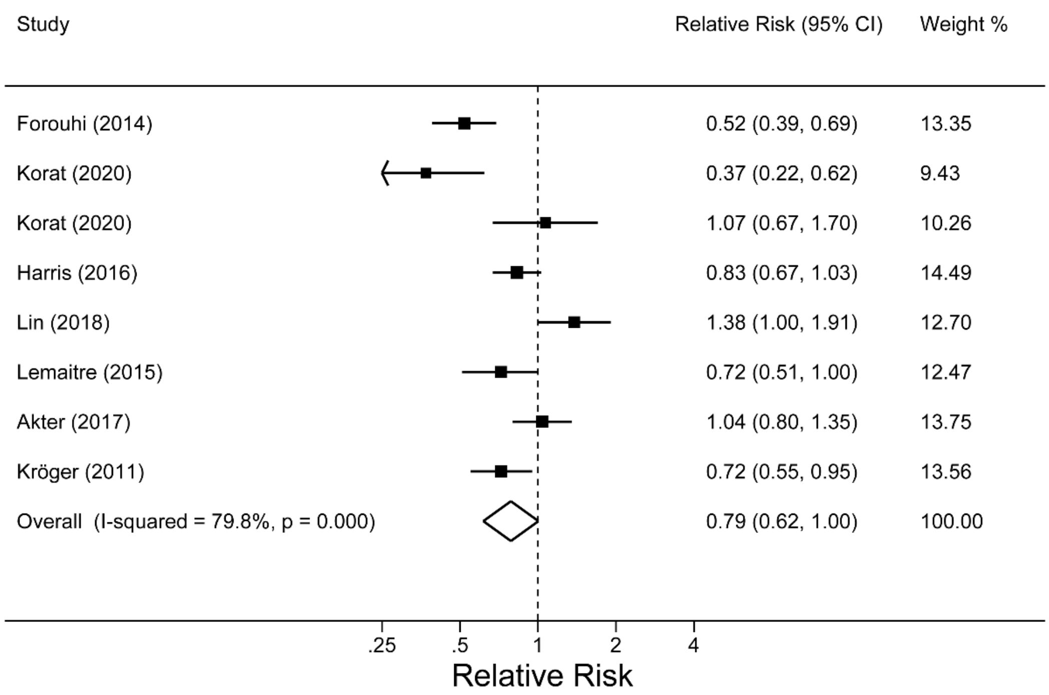

95%CI, 95% confidence interval.

Figure S8. Pooled relative risk of type 2 diabetes for the highest versus lowest categories of behenic acid (22:0) biomarker level.

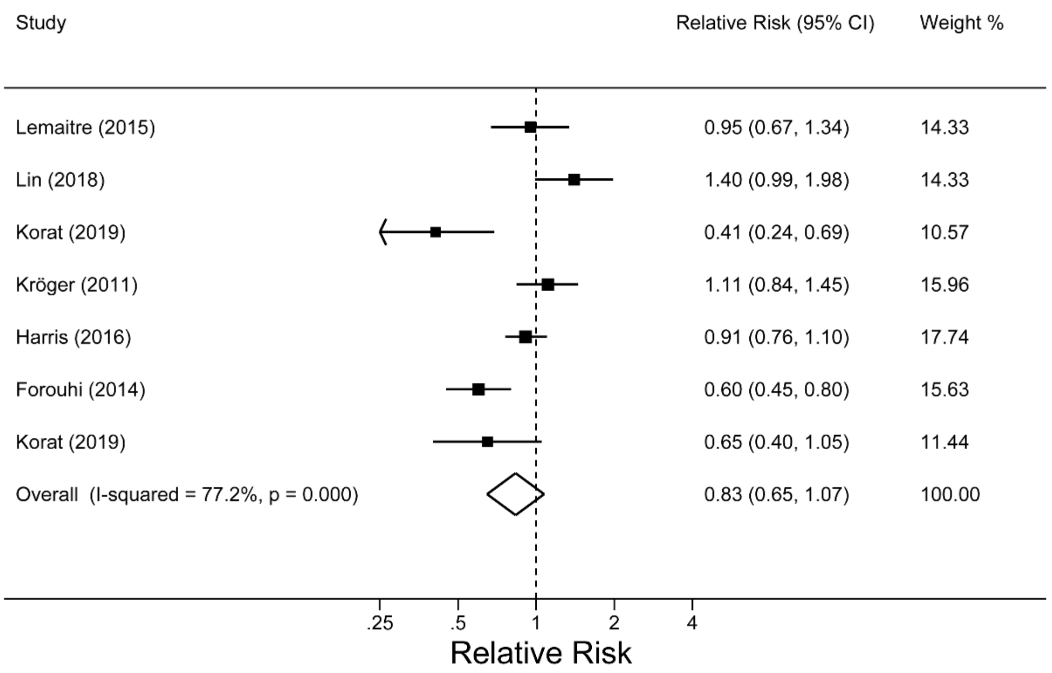

95%CI, 95% confidence interval.

Figure S9. Pooled relative risk of type 2 diabetes for the highest versus lowest categories of lignoceric acid (24:0) biomarker level.

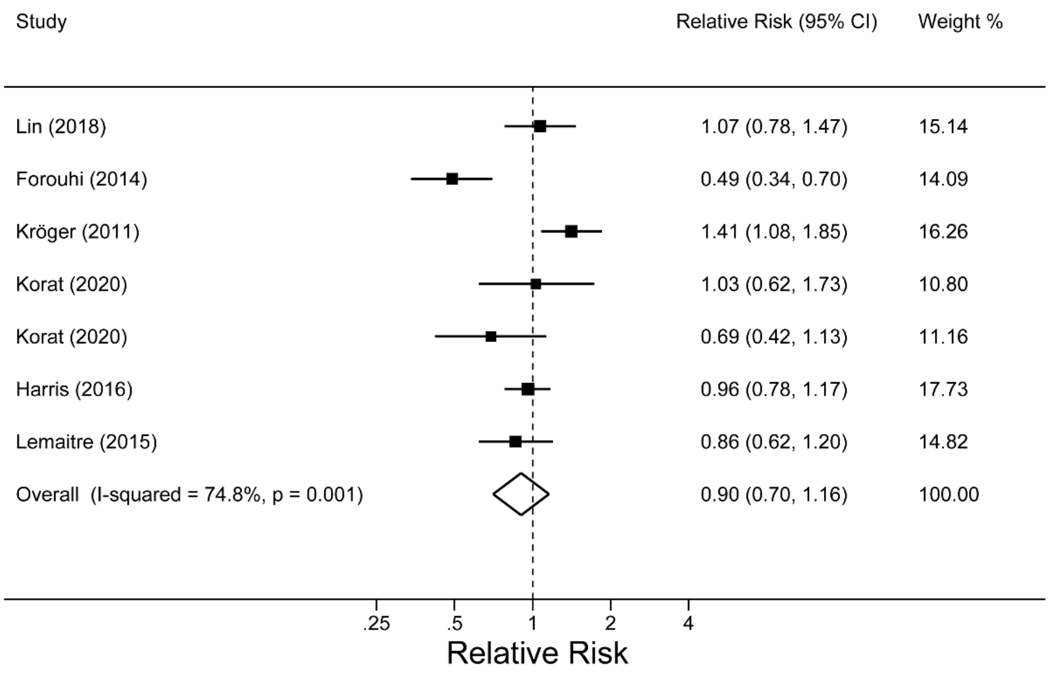

95%CI, 95% confidence interval.

Figure S10. Subgroup analyses of total saturated fatty acid biomarkers and cardiometabolic diseases.

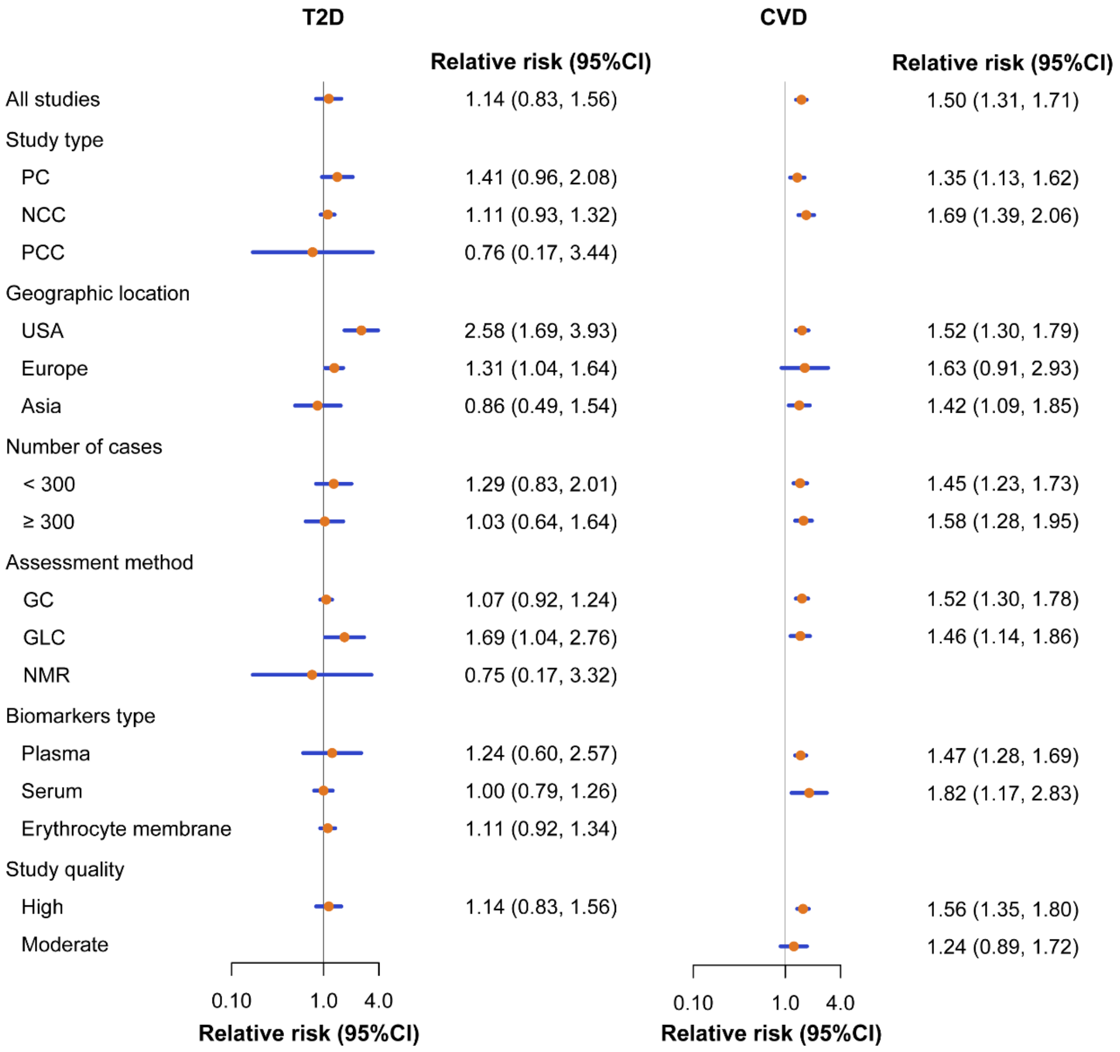

95%CI, 95% confidence interval; CVD, cardiovascular disease; GC, gas chromatography; GLC, gas-liquid chromatography; NCC, nested case-control study; NMR, nuclear magnetic resonance-based profiling; PC, prospective cohort study; PCC, prospective case-cohort study; T2D, type 2 diabetes; USA, the United States of America.

Figure S11. Subgroup analyses of even-chain saturated fatty acid biomarkers and cardiometabolic diseases.

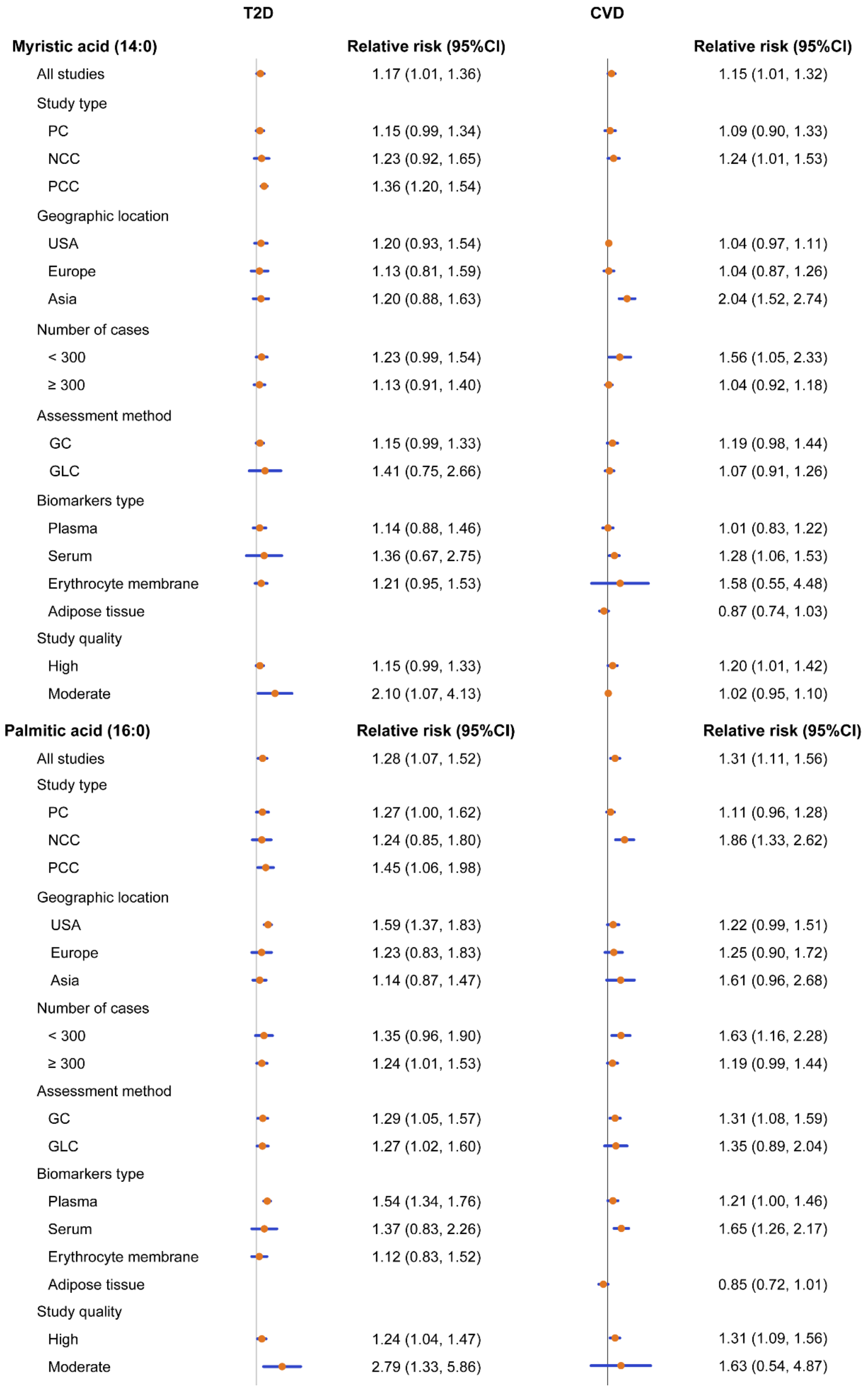

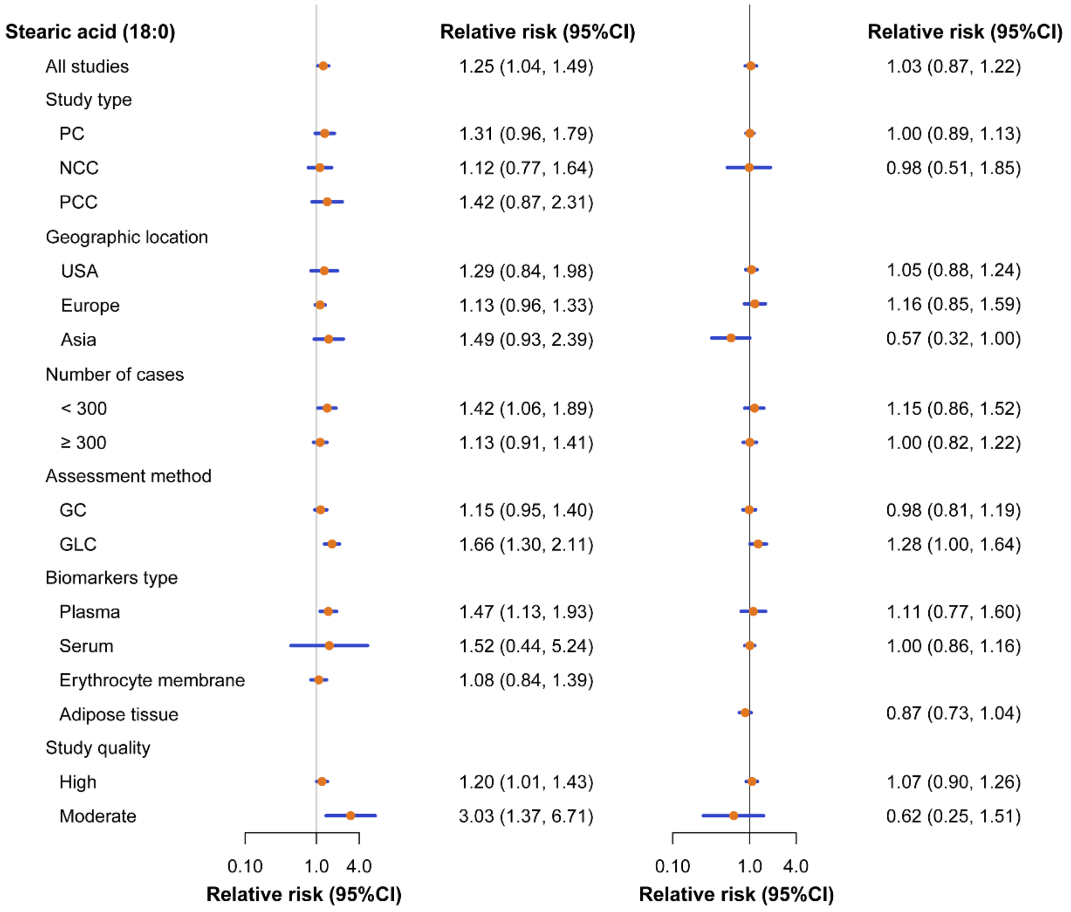

95%CI, 95% confidence interval; CVD, cardiovascular disease; GC, gas chromatography; GLC, gas-liquid chromatography; NCC, nested case-control study; NMR, nuclear magnetic resonance-based profiling; PC, prospective cohort study; PCC, prospective case-cohort study; T2D, type 2 diabetes; USA, the United States of America.

Figure S12. Subgroup analyses of odd-chain saturated fatty acid biomarkers and cardiometabolic diseases.

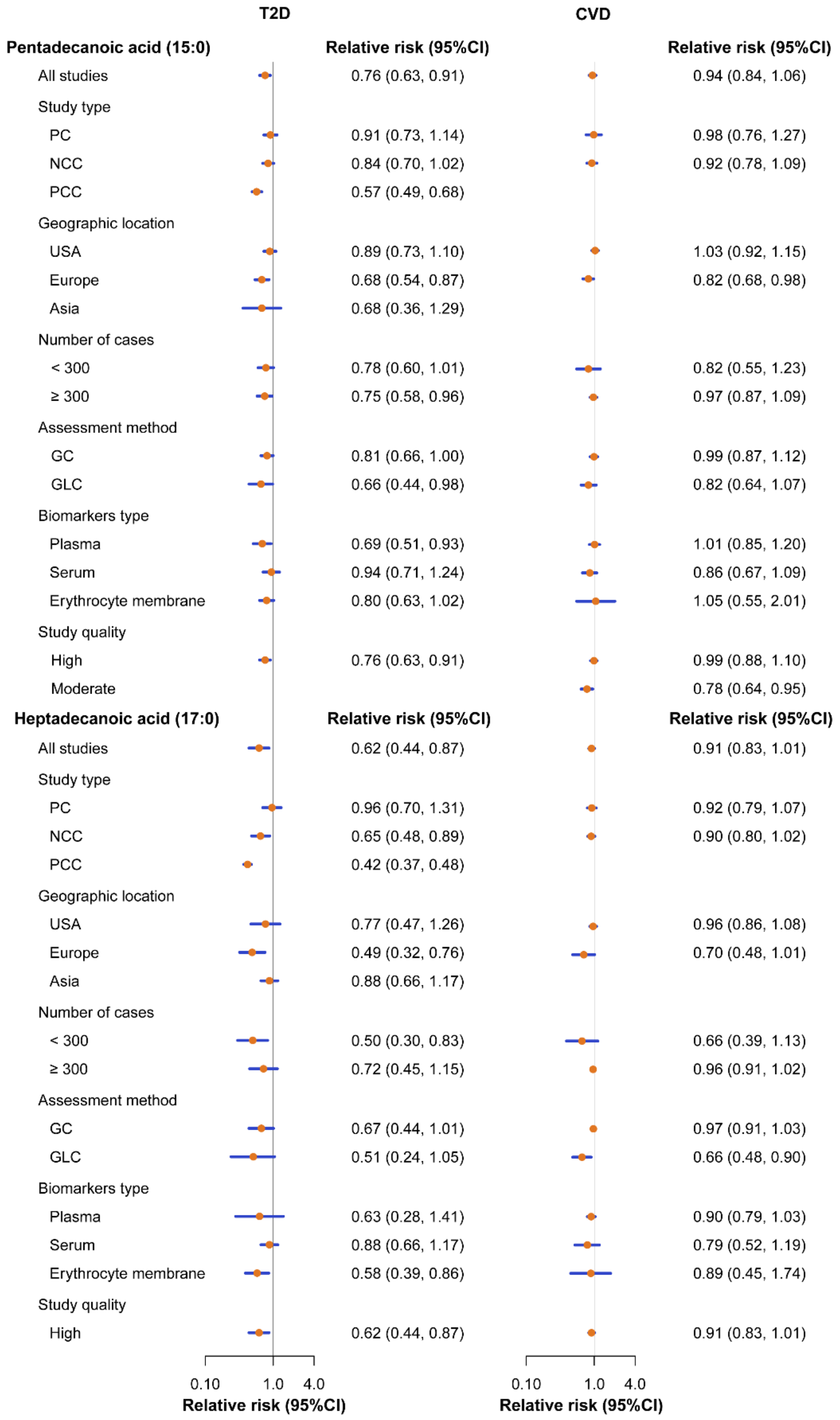

95%CI, 95% confidence interval; CVD, cardiovascular disease; GC, gas chromatography; GLC, gas-liquid chromatography; NCC, nested case-control study; NMR, nuclear magnetic resonance-based profiling; PC, prospective cohort study; PCC, prospective case-cohort study; T2D, type 2 diabetes; USA, the United States of America.

Figure S13. Pooled relative risk of cardiovascular disease for the highest versus lowest categories of total saturated fatty acid biomarker level.

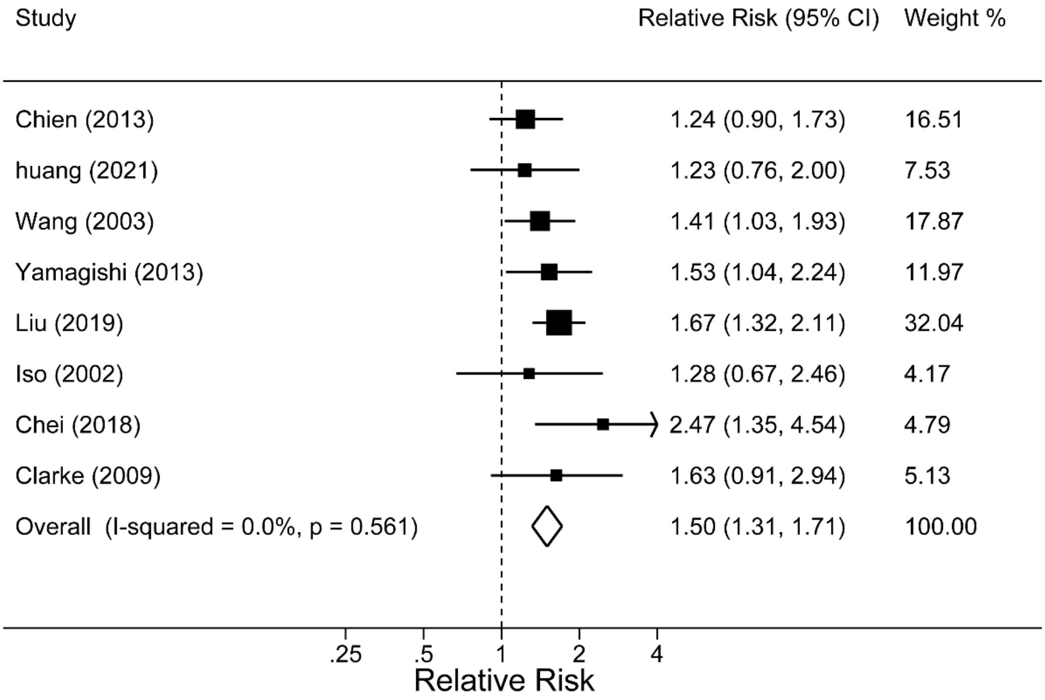

95%CI, 95% confidence interval.

Figure S14. Pooled relative risk of cardiovascular disease for the highest versus lowest categories of palmitic acid (16:0) biomarker level.

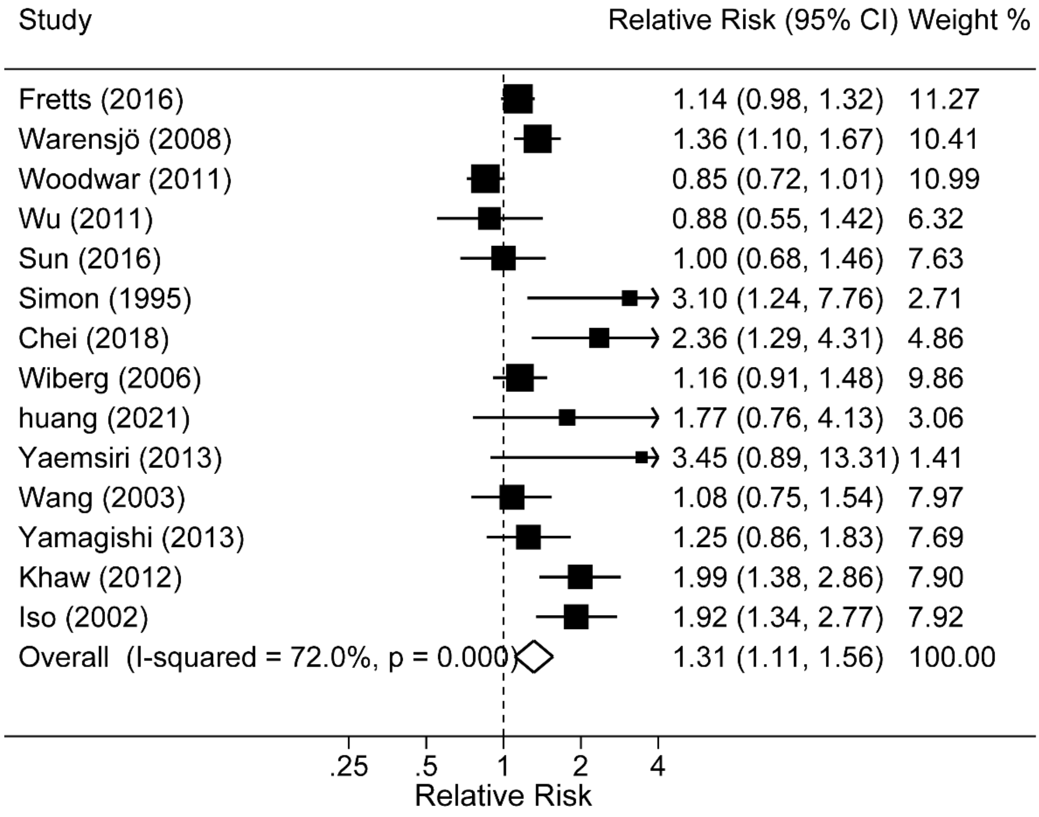

95%CI, 95% confidence interval.

Figure S15. Pooled relative risk of cardiovascular disease for the highest versus lowest categories of myristic acid (14:0) biomarker level.

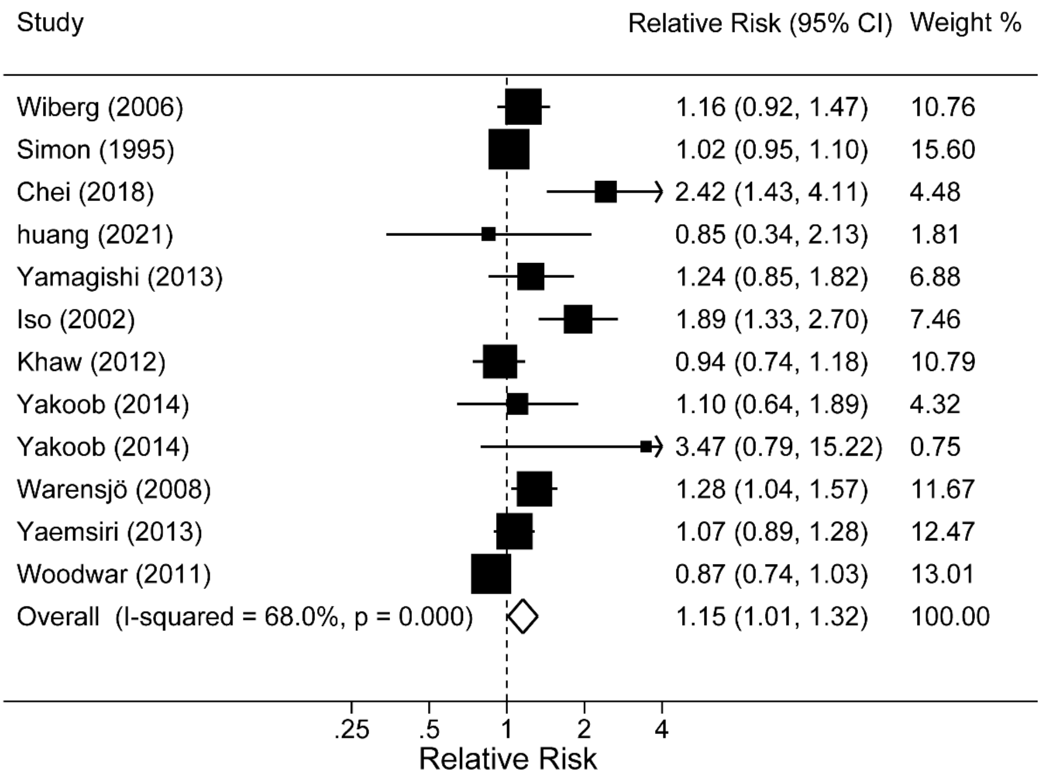

95%CI, 95% confidence interval.

Figure S16. Pooled relative risk of cardiovascular disease for the highest versus lowest categories of sum of pentadecanoic acid (15:0) and margaric acid (17:0) biomarker level.

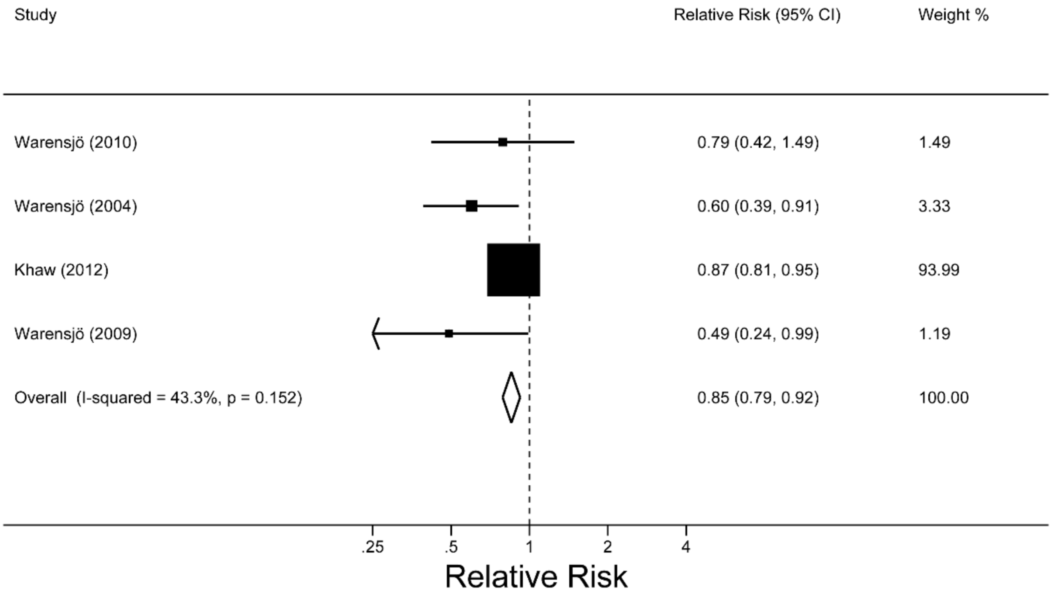

95%CI, 95% confidence interval.

Figure S17. Pooled relative risk of cardiovascular disease for the highest versus lowest categories of lignoceric acid (24:0) biomarker level.

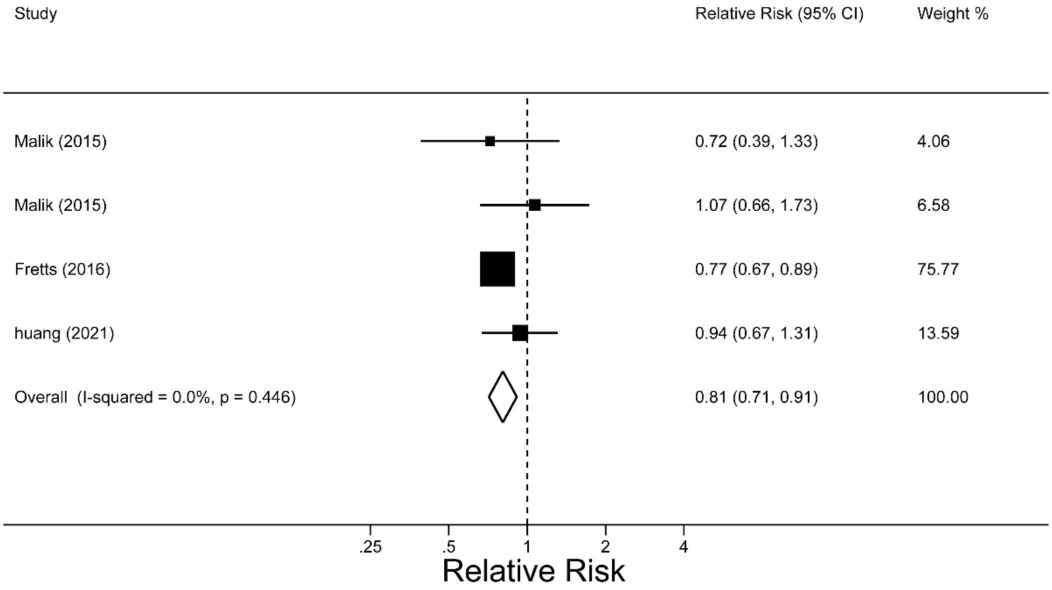

95%CI, 95% confidence interval.

Figure S18. Pooled relative risk of cardiovascular disease for the highest versus lowest categories of pentadecanoic acid (15:0) biomarker level.

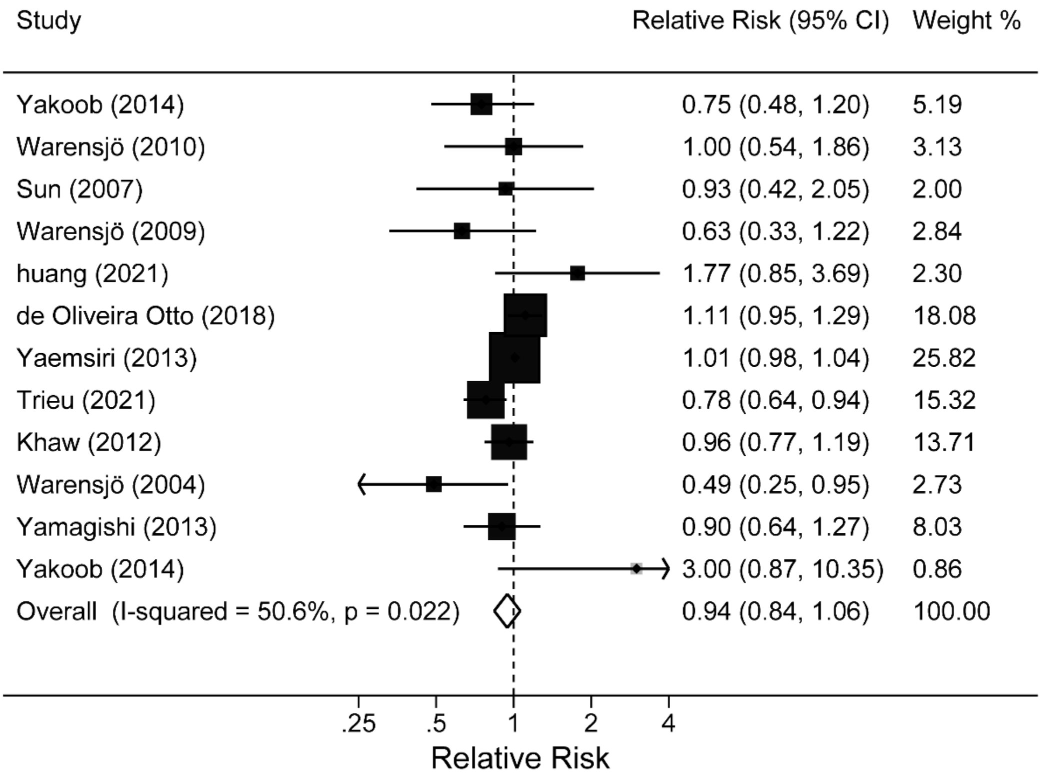

95%CI, 95% confidence interval.

Figure S19. Pooled relative risk of cardiovascular disease for the highest versus lowest categories of margaric acid (17:0) biomarker level.

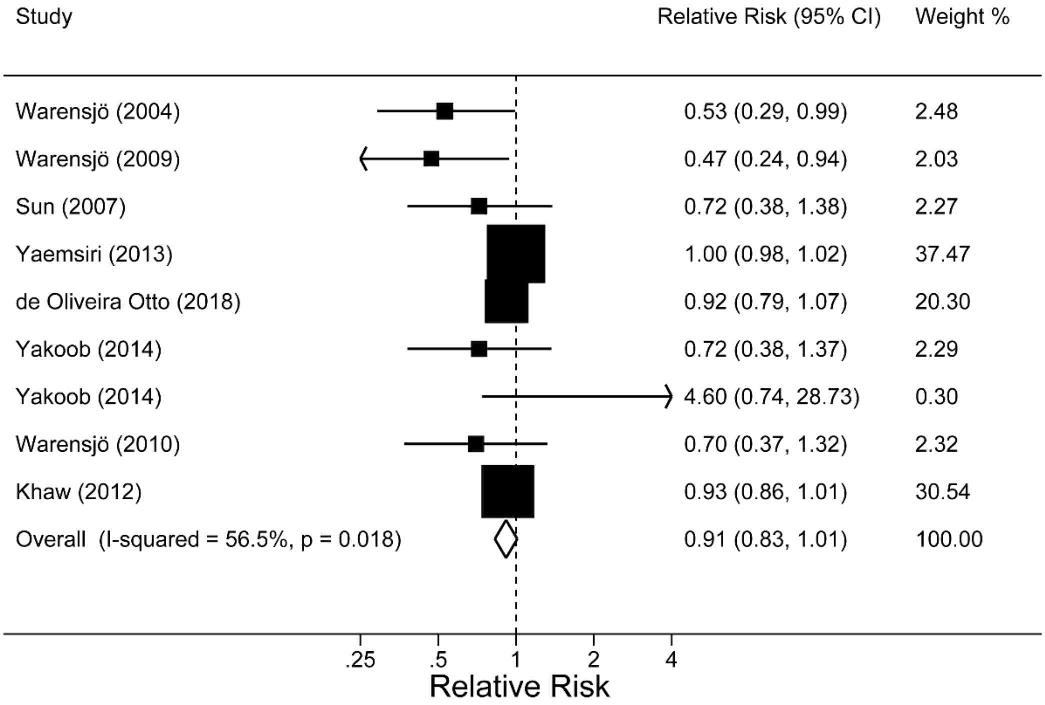

95%CI, 95% confidence interval.

Figure S20. Pooled relative risk of cardiovascular disease for the highest versus lowest categories of stearic acid (18:0) biomarker level.

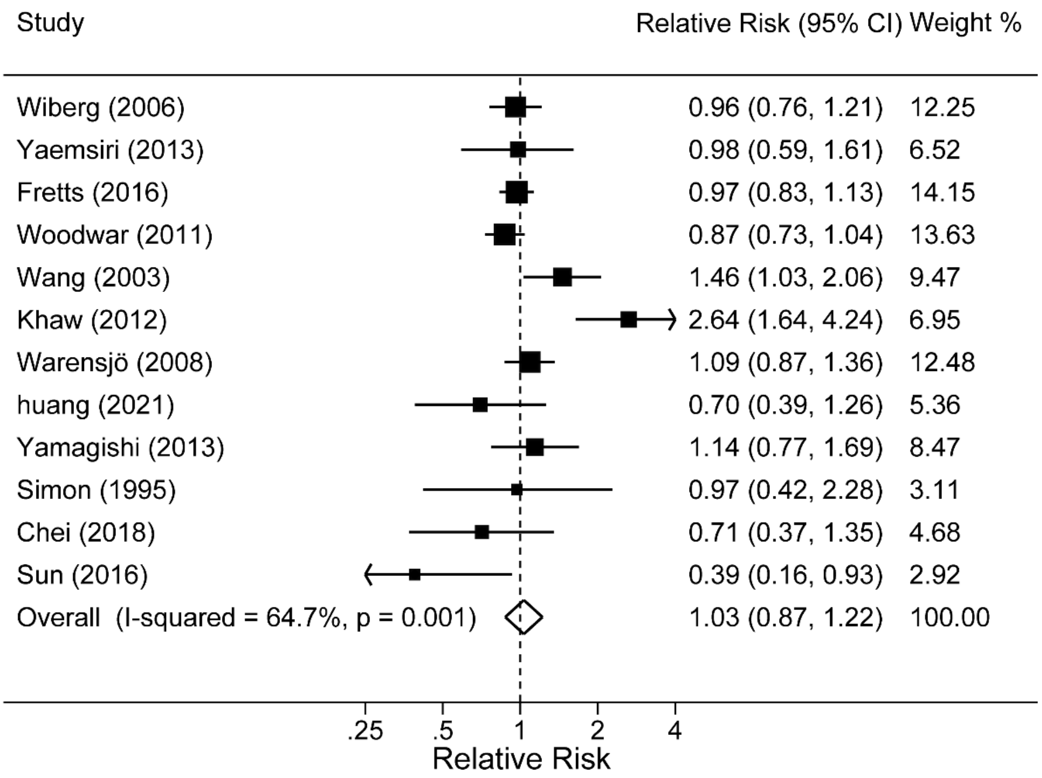

95%CI, 95% confidence interval.

Figure S21. Pooled relative risk of cardiovascular disease for the highest versus lowest categories of arachidic acid (20:0) biomarker level.

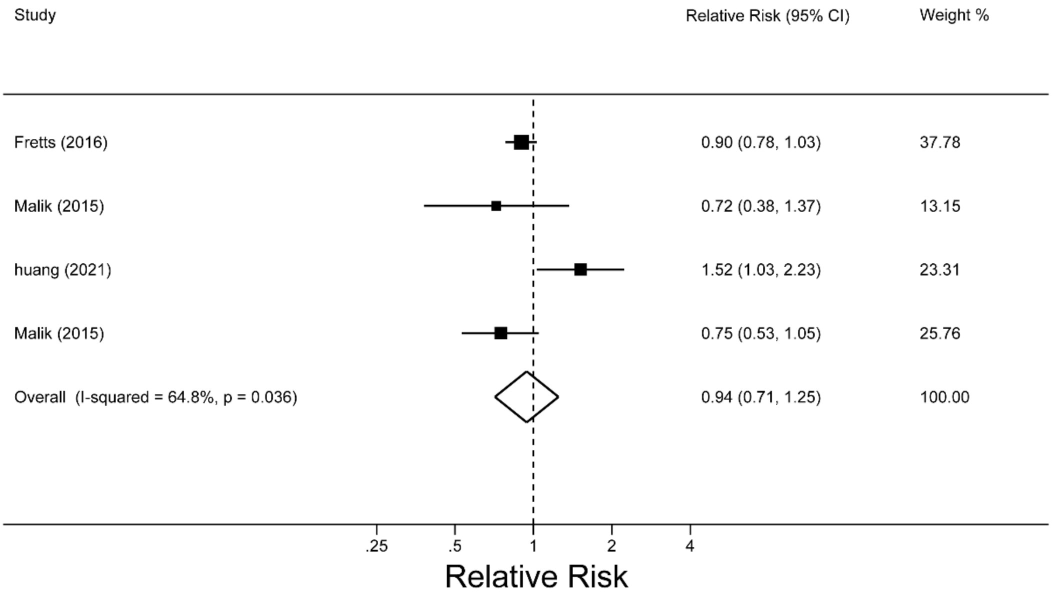

95%CI, 95% confidence interval.

Figure S22. Pooled relative risk of cardiovascular disease for the highest versus lowest categories of behenic acid (22:0) biomarker level.

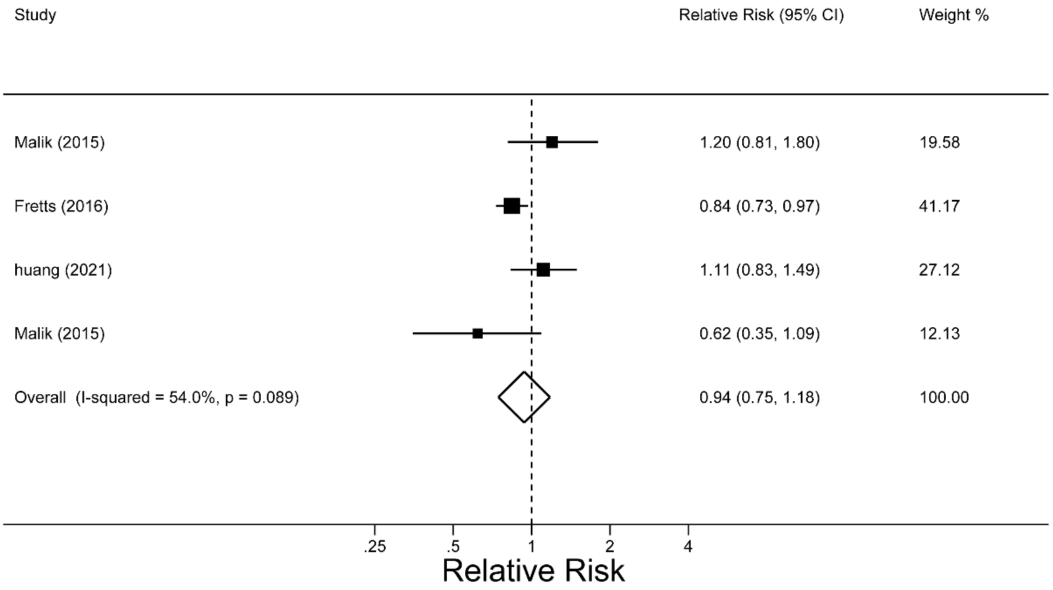

95%CI, 95% confidence interval.

Figure S23. Pooled relative risk of coronary heart disease for the highest versus lowest categories of total saturated fatty acid biomarker level.

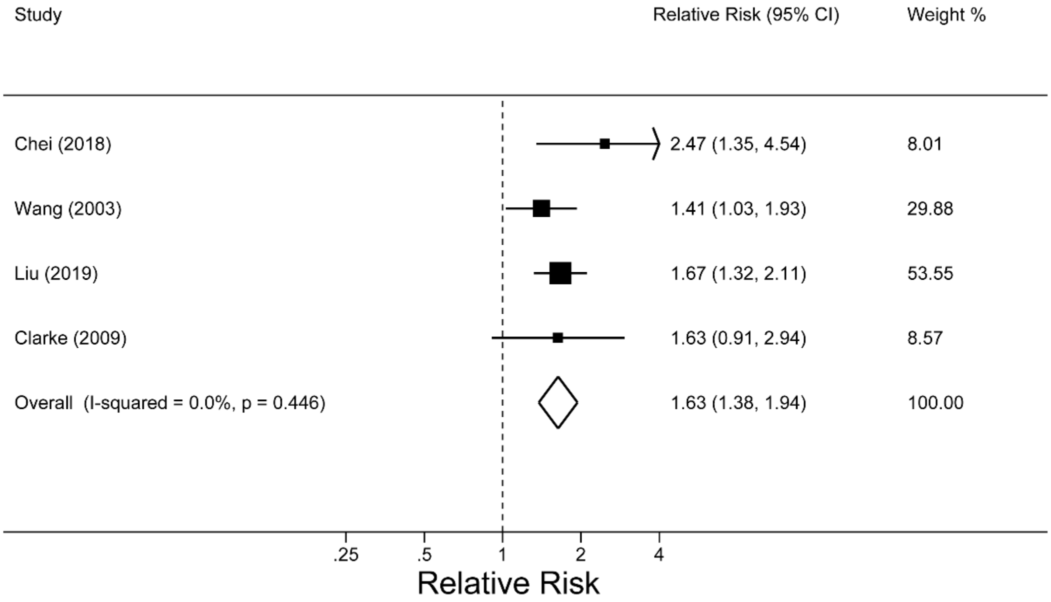

95%CI, 95% confidence interval.

Figure S24. Pooled relative risk of coronary heart disease for the highest versus lowest categories of palmitic acid (16:0) biomarker level.

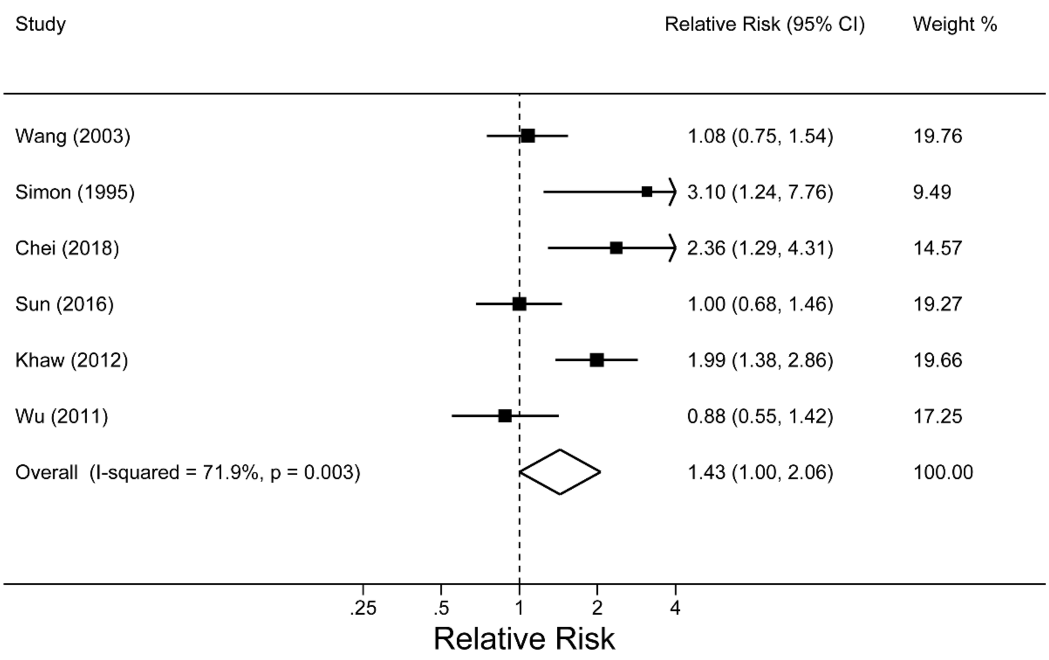

95%CI, 95% confidence interval.

Figure S25. Pooled relative risk of coronary heart disease for the highest versus lowest categories of pentadecanoic acid (15:0) biomarker level.

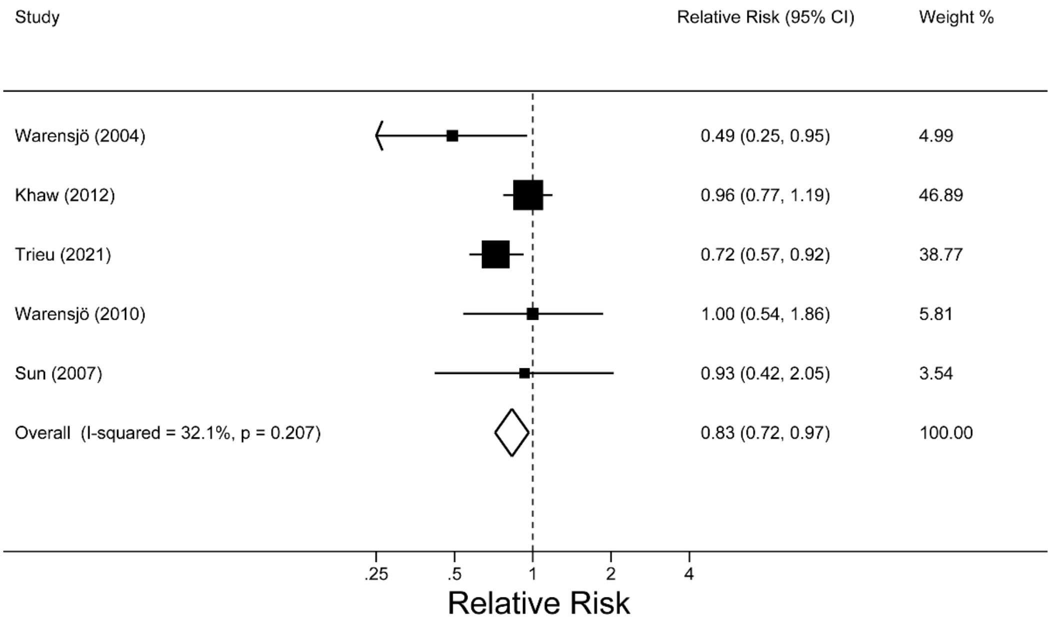

95%CI, 95% confidence interval.

Figure S26. Pooled relative risk of coronary heart disease for the highest versus lowest categories of margaric acid (17:0) biomarker level.

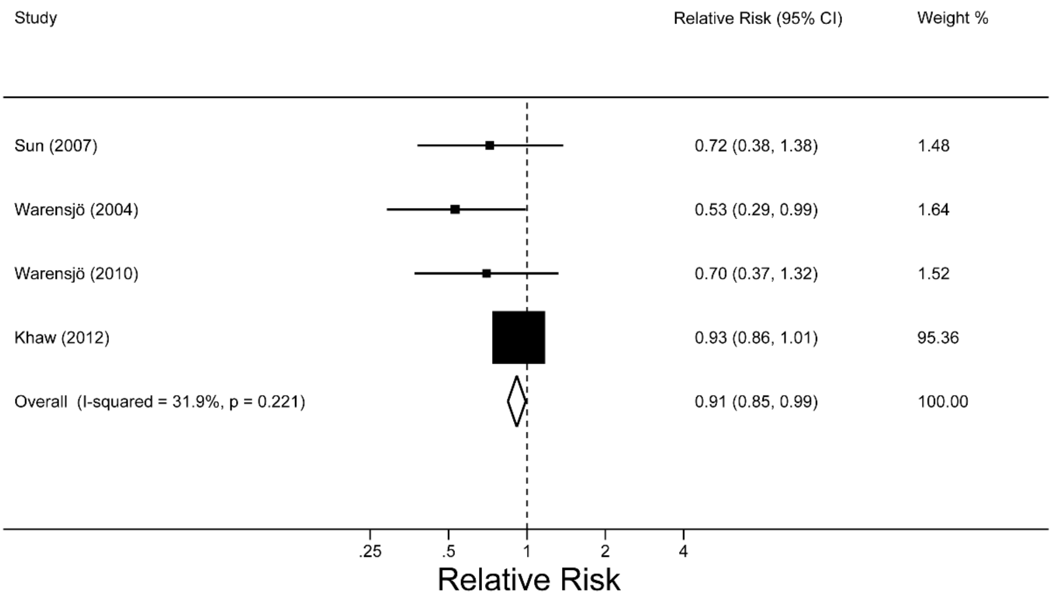

95%CI, 95% confidence interval.

Figure S27. Pooled relative risk of coronary heart disease for the highest versus lowest categories of sum of pentadecanoic acid (15:0) and margaric acid (17:0) biomarker level.

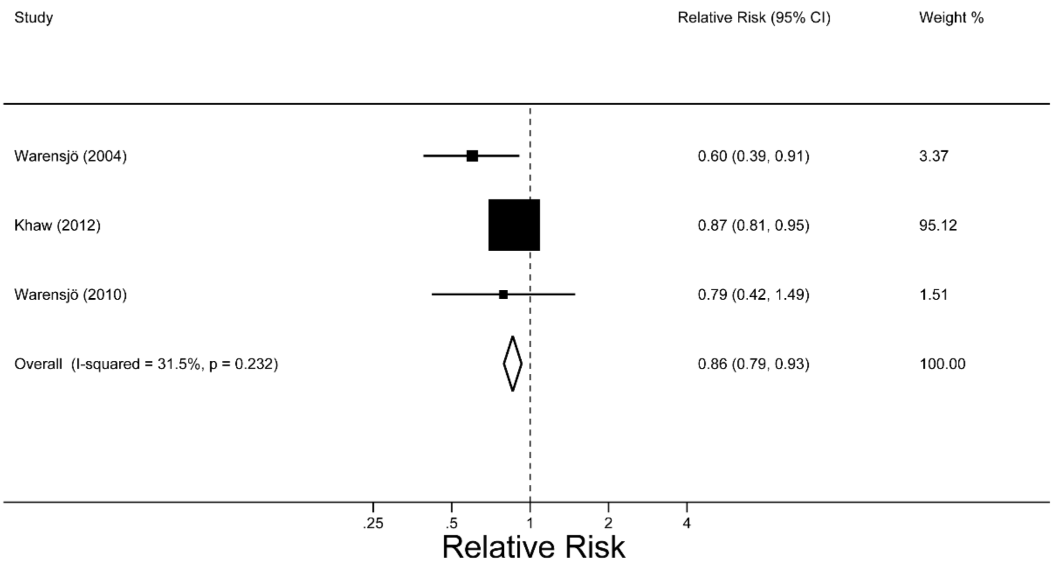

95%CI, 95% confidence interval.

Figure S28. Pooled relative risk of coronary heart disease for the highest versus lowest categories of myristic acid (14:0) biomarker level.

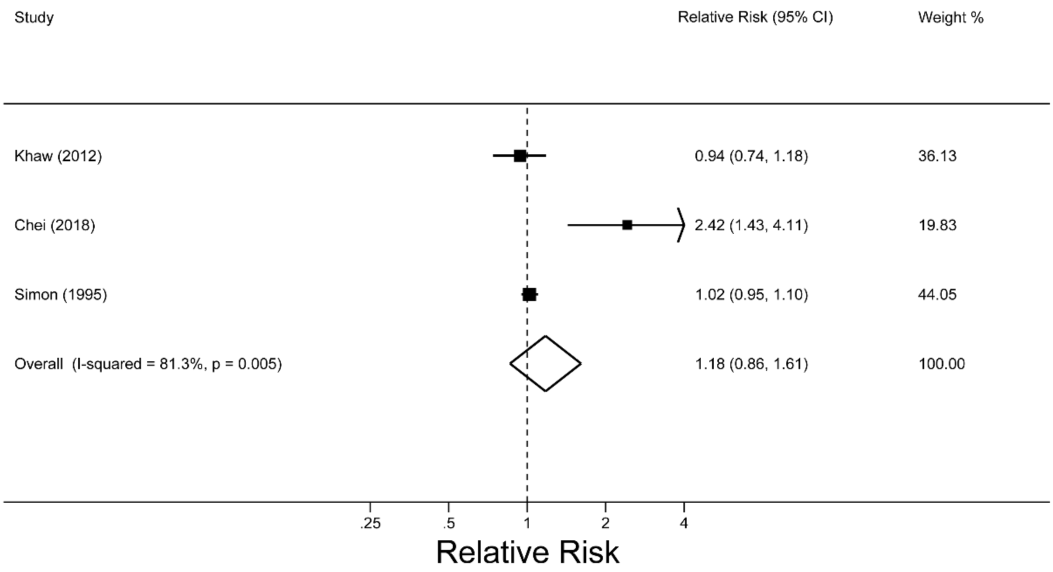

95%CI, 95% confidence interval.

Figure S29. Pooled relative risk of coronary heart disease for the highest versus lowest categories of stearic acid (18:0) biomarker level.

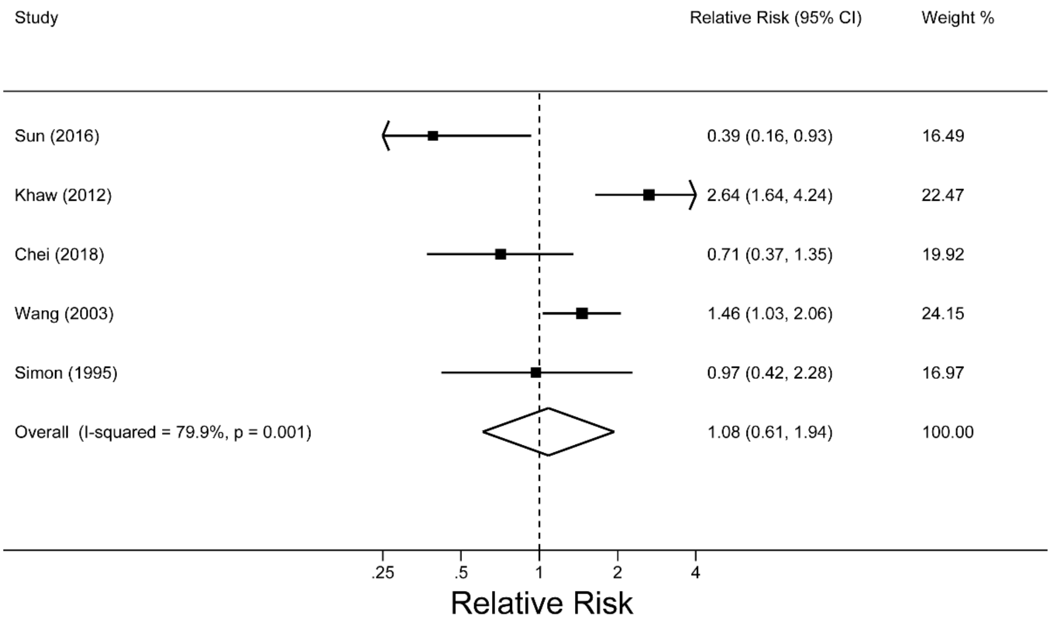

95%CI, 95% confidence interval.

Figure S30. Pooled relative risk of stroke for the highest versus lowest categories of total saturated fatty acid biomarker level.

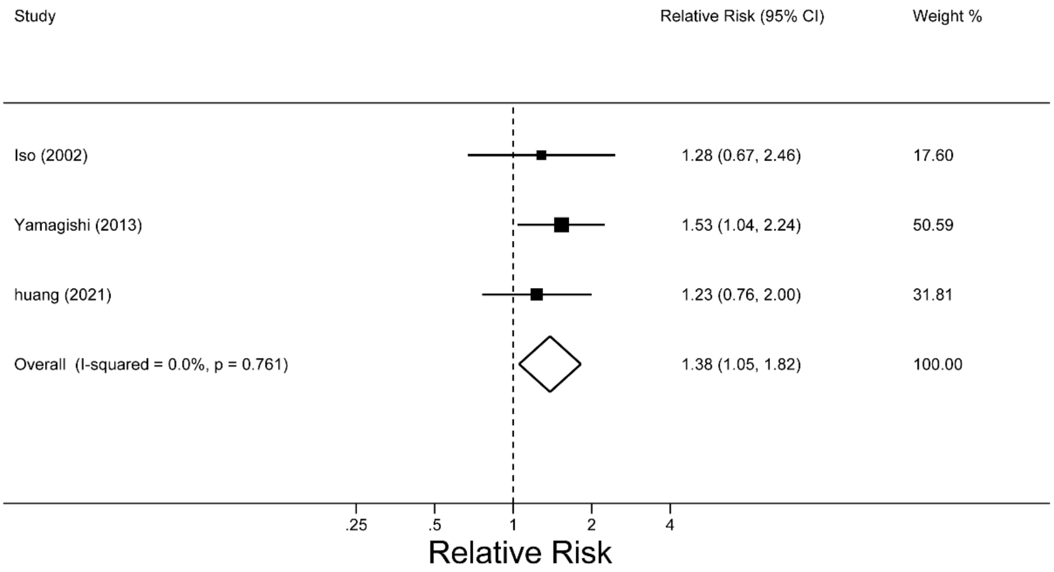

95%CI, 95% confidence interval.

Figure S31. Pooled relative risk of stroke for the highest versus lowest categories of palmitic acid (16:0) biomarker level.

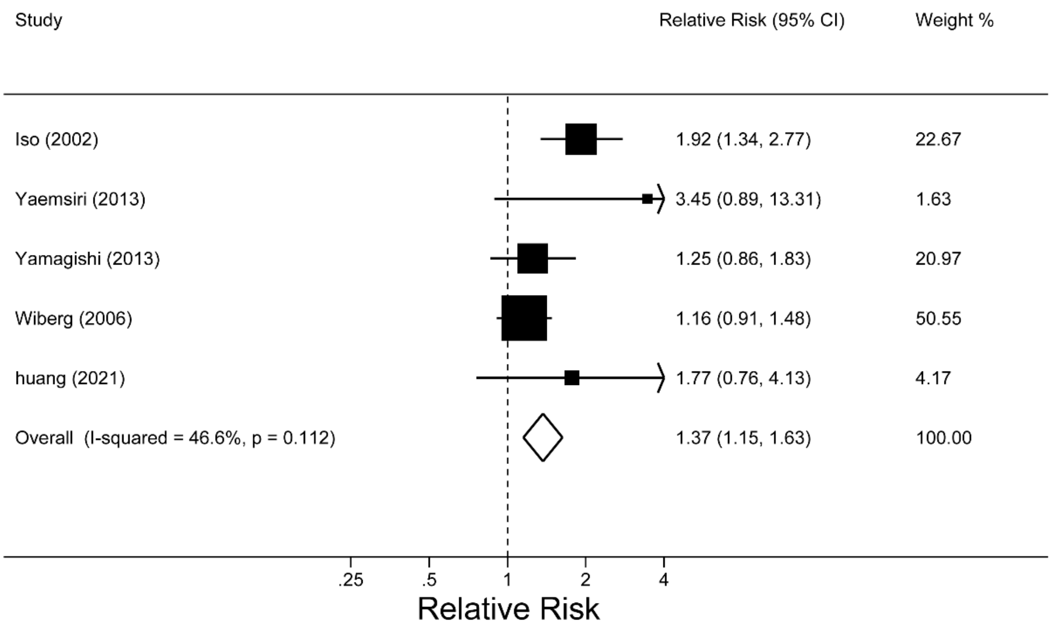

95%CI, 95% confidence interval.

Figure S32. Pooled relative risk of stroke for the highest versus lowest categories of myristic acid (14:0) biomarker level.

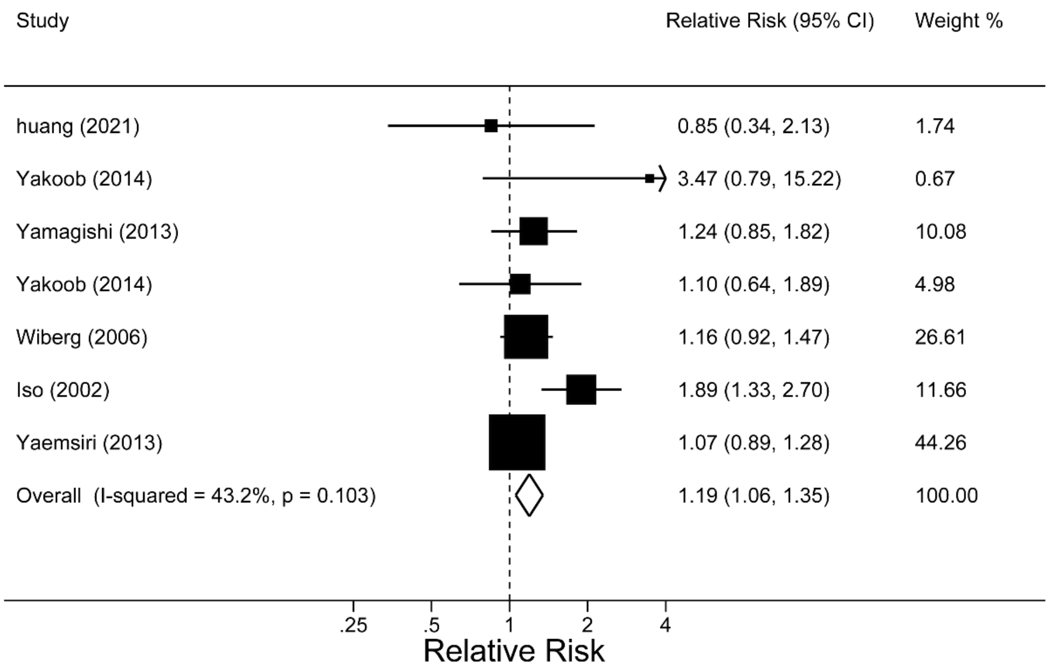

95%CI, 95% confidence interval.

Figure S33. Pooled relative risk of stroke for the highest versus lowest categories of pentadecanoic acid (15:0) biomarker level.

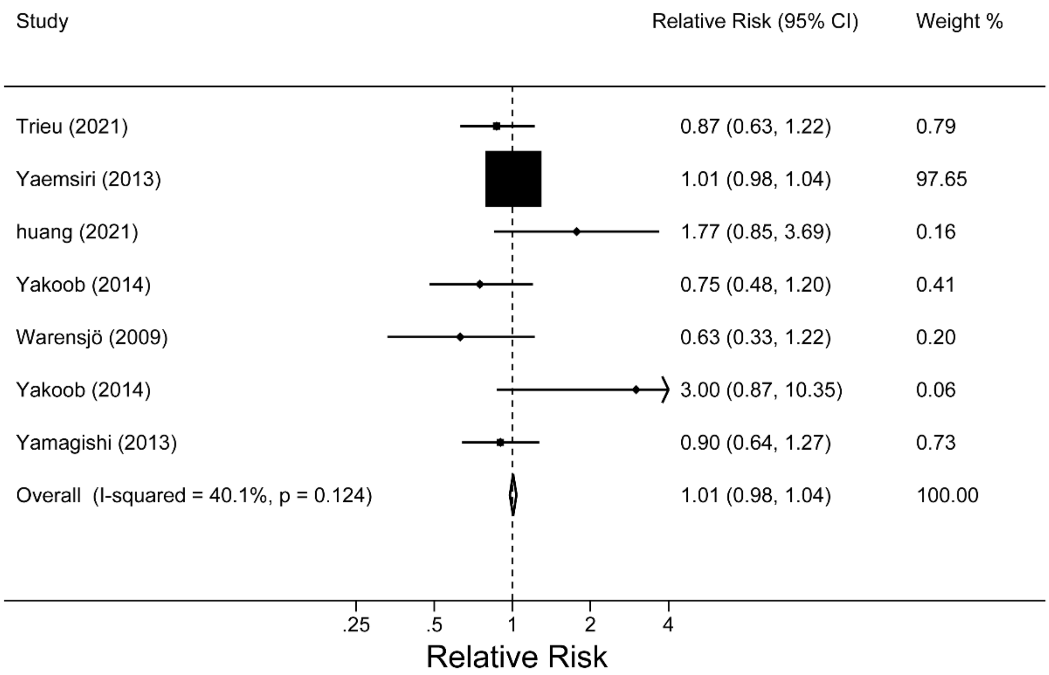

95%CI, 95% confidence interval.

Figure S34. Pooled relative risk of stroke for the highest versus lowest categories of margaric acid (17:0) biomarker level.

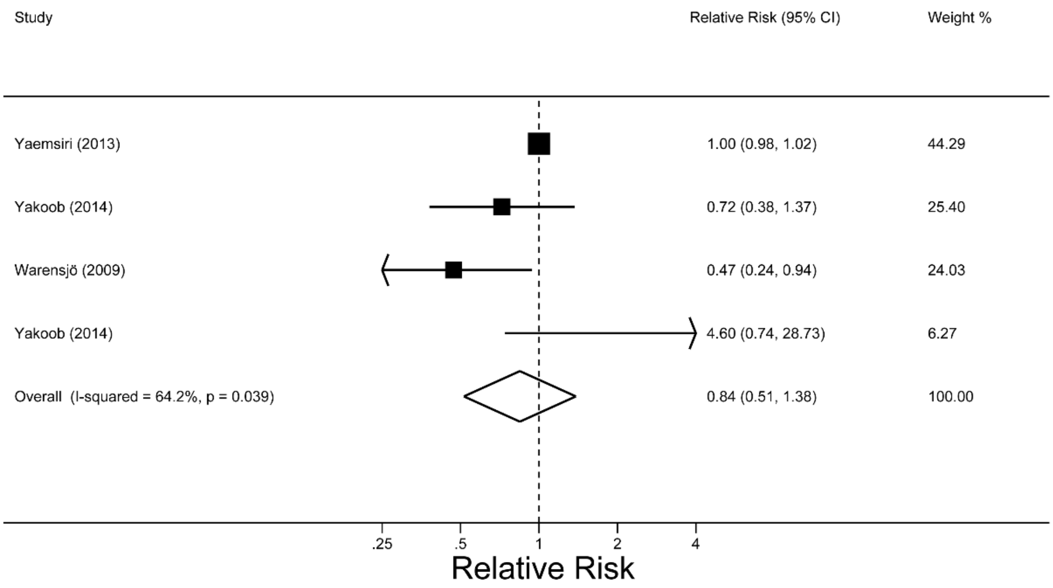

95%CI, 95% confidence interval.

Figure S35. Pooled relative risk of stroke for the highest versus lowest categories of stearic acid (18:0) biomarker level.

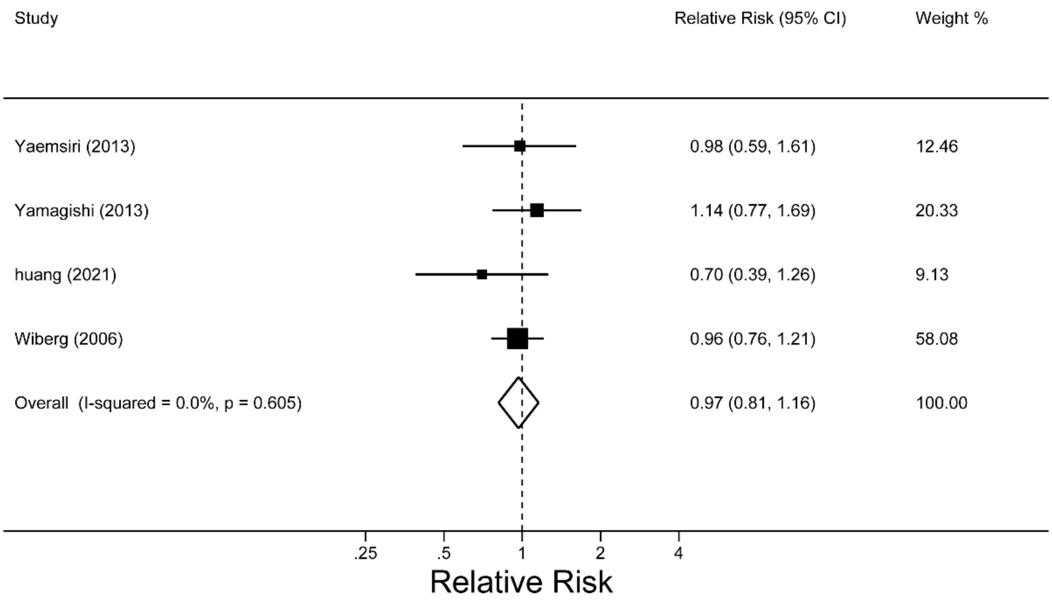

95%CI, 95% confidence interval.

Figure S36. Funnel plot of relative risk (RR) for type 2 diabetes comparing the lowest with the highest categories for total saturated fatty acid biomarker level.

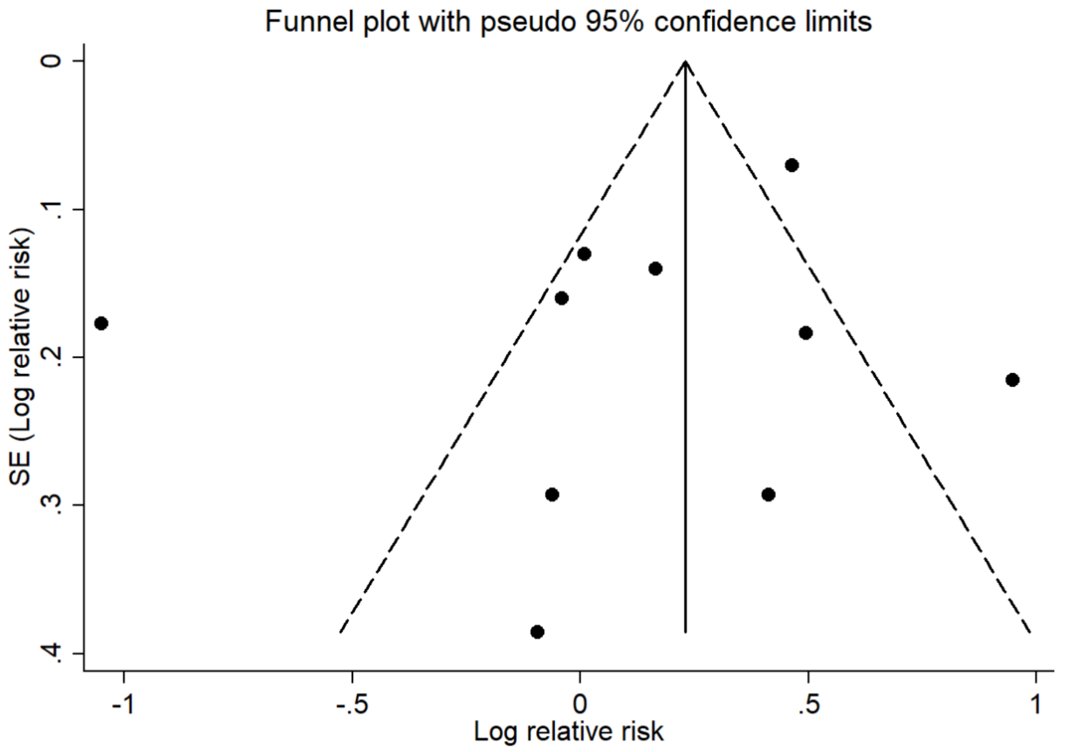

The vertical line represents the pooled RR. The vertical line represents the pooled RR. The dashed lines represent the pseudo-95% confidence interval of the RR. The circles represent risk estimates for each cohort, and the horizontal line represents standard errors of the RR. RR, relative risk.

Figure S37. Funnel plot of relative risk (RR) for type 2 diabetes comparing the lowest with the highest categories for myristic acid (14:0) biomarker level.

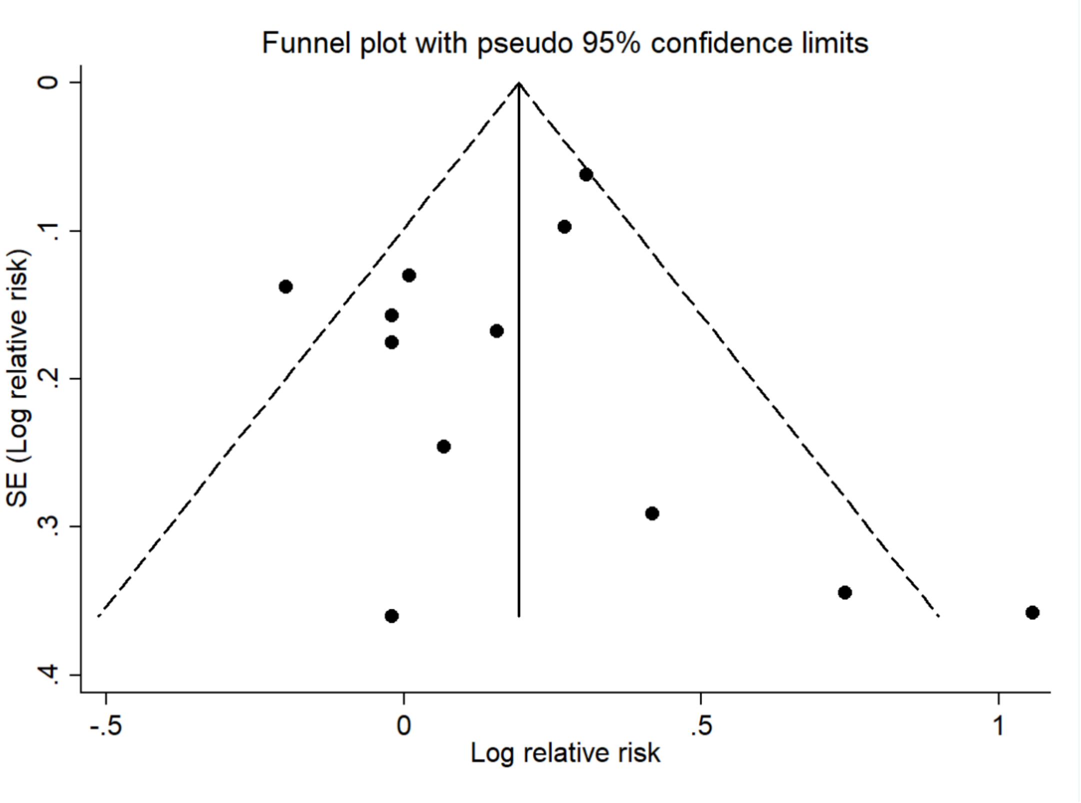

The vertical line represents the pooled RR. The dashed lines represent the pseudo-95% confidence interval of the RR. The circles represent risk estimates for each cohort, and the horizontal line represents standard errors of the RR. RR, relative risk.

Figure S38. Funnel plot of relative risk (RR) for type 2 diabetes comparing the lowest with the highest categories for palmitic acid (16:0) biomarker level.

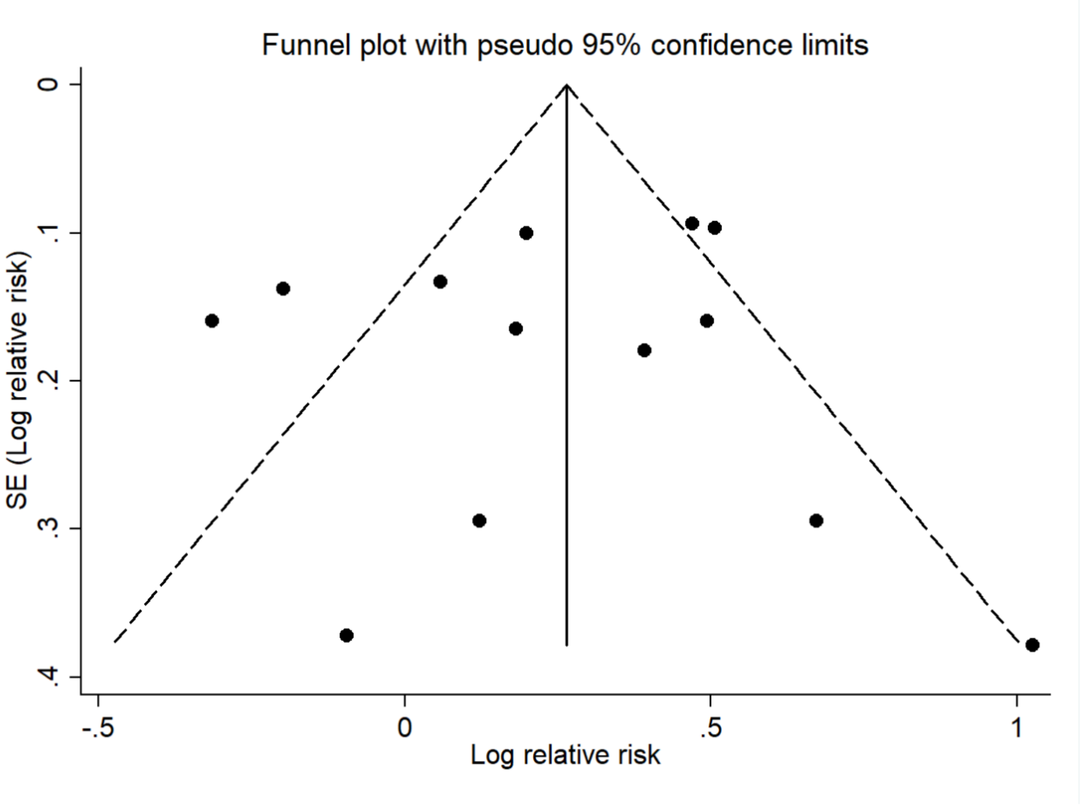

The vertical line represents the pooled RR. The dashed lines represent the pseudo-95% confidence interval of the RR. The circles represent risk estimates for each cohort, and the horizontal line represents standard errors of the RR. RR, relative risk.

Figure S39. Funnel plot of relative risk (RR) for type 2 diabetes comparing the lowest with the highest categories for stearic acid (18:0) biomarker level.

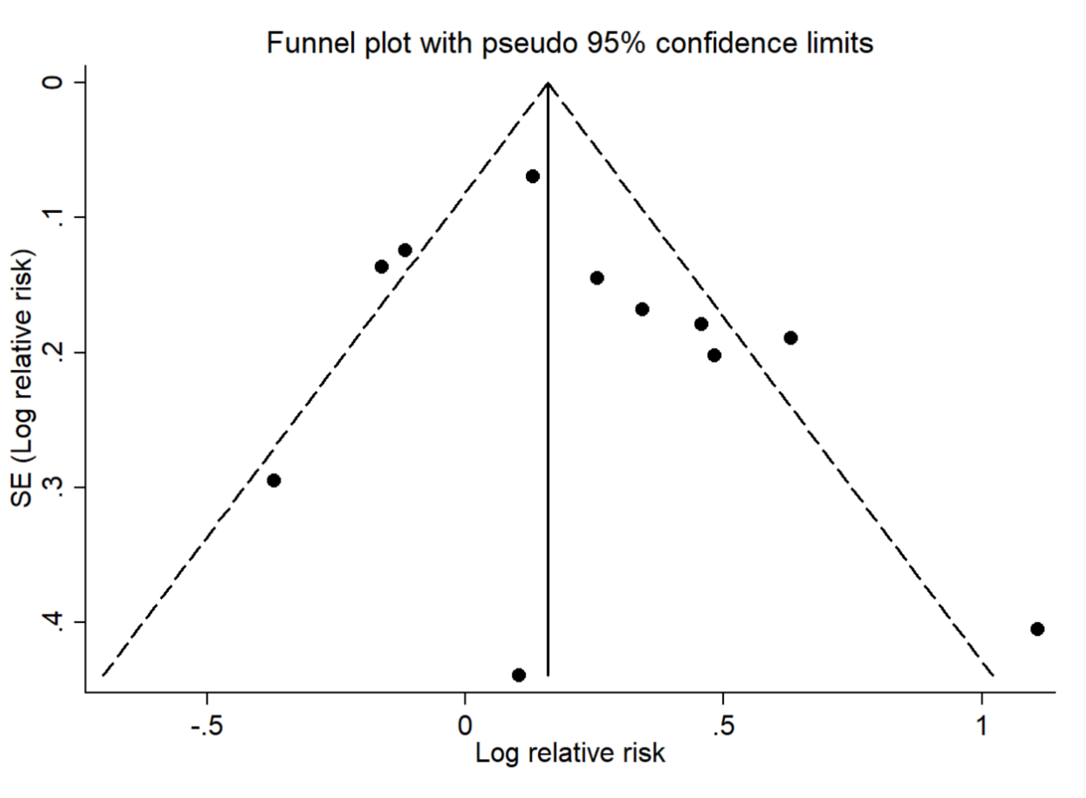

The vertical line represents the pooled RR. The dashed lines represent the pseudo-95% confidence interval of the RR. The circles represent risk estimates for each cohort, and the horizontal line represents standard errors of the RR. RR, relative risk.

Figure S40. Funnel plot of relative risk (RR) for type 2 diabetes comparing the lowest with the highest categories for pentadecanoic acid (15:0) biomarker level.

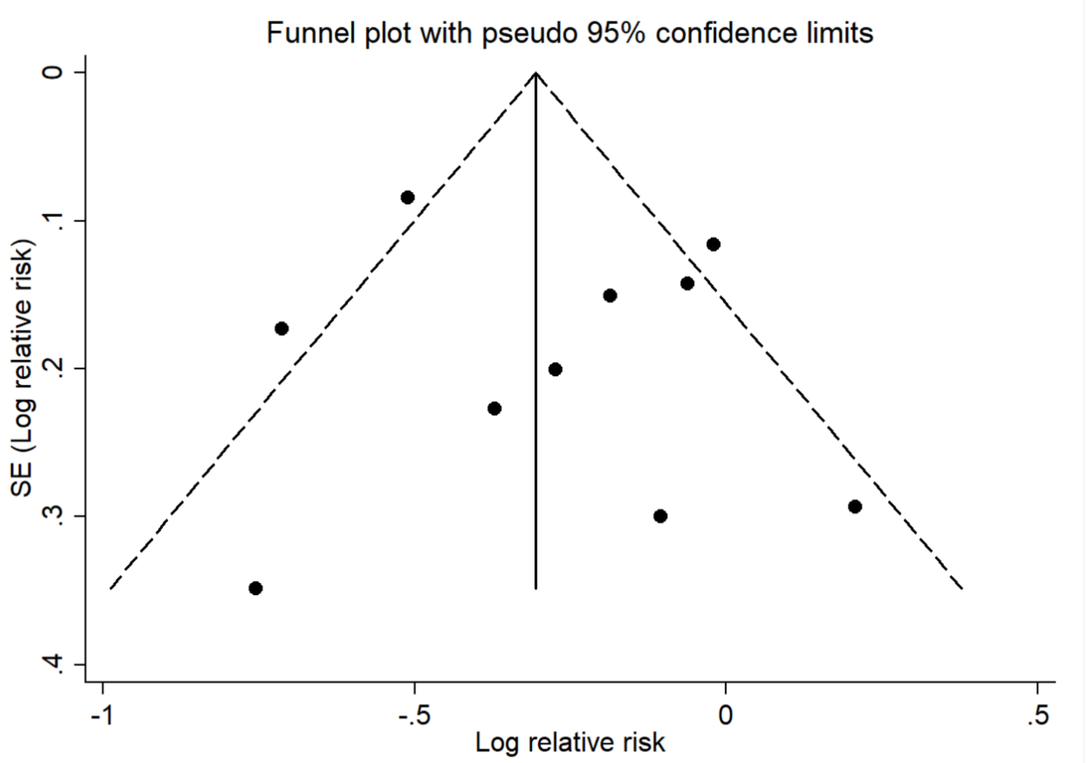

The vertical line represents the pooled RR. The dashed lines represent the pseudo-95% confidence interval of the RR. The circles represent risk estimates for each cohort, and the horizontal line represents standard errors of the RR. RR, relative risk.

Figure S41. Funnel plot of relative risk (RR) for type 2 diabetes comparing the lowest with the highest categories for margaric acid (17:0) biomarker level.

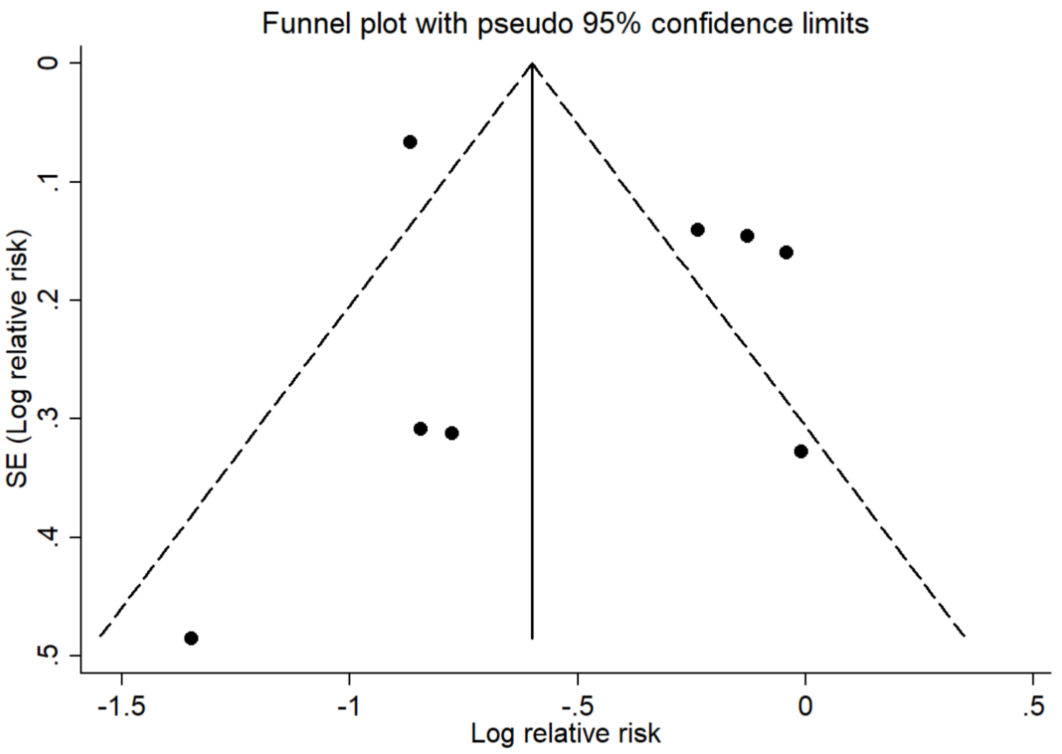

The vertical line represents the pooled RR. The dashed lines represent the pseudo-95% confidence interval of the RR. The circles represent risk estimates for each cohort, and the horizontal line represents standard errors of the RR. RR, relative risk.

Figure S42. Funnel plot of relative risk (RR) for type 2 diabetes comparing the lowest with the highest categories for arachidic acid (20:0) biomarker level.

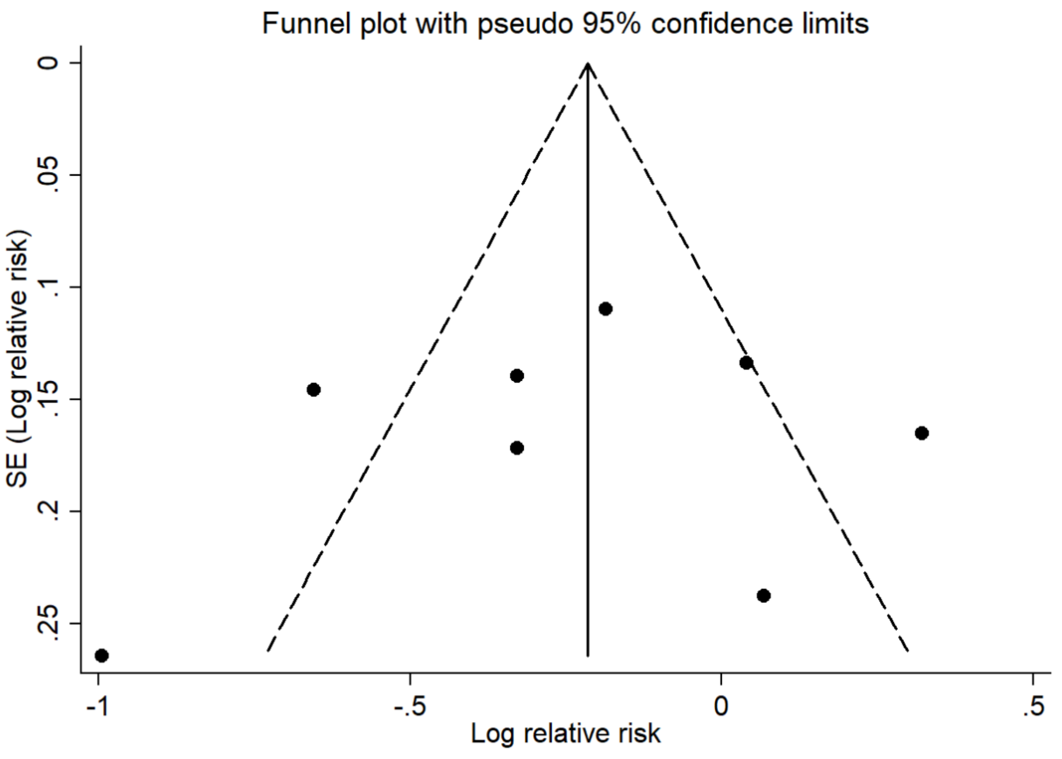

The vertical line represents the pooled RR. The dashed lines represent the pseudo-95% confidence interval of the RR. The circles represent risk estimates for each cohort, and the horizontal line represents standard errors of the RR. RR, relative risk.

Figure S43. Funnel plot of relative risk (RR) for type 2 diabetes comparing the lowest with the highest categories for behenic acid (22:0) biomarker level.

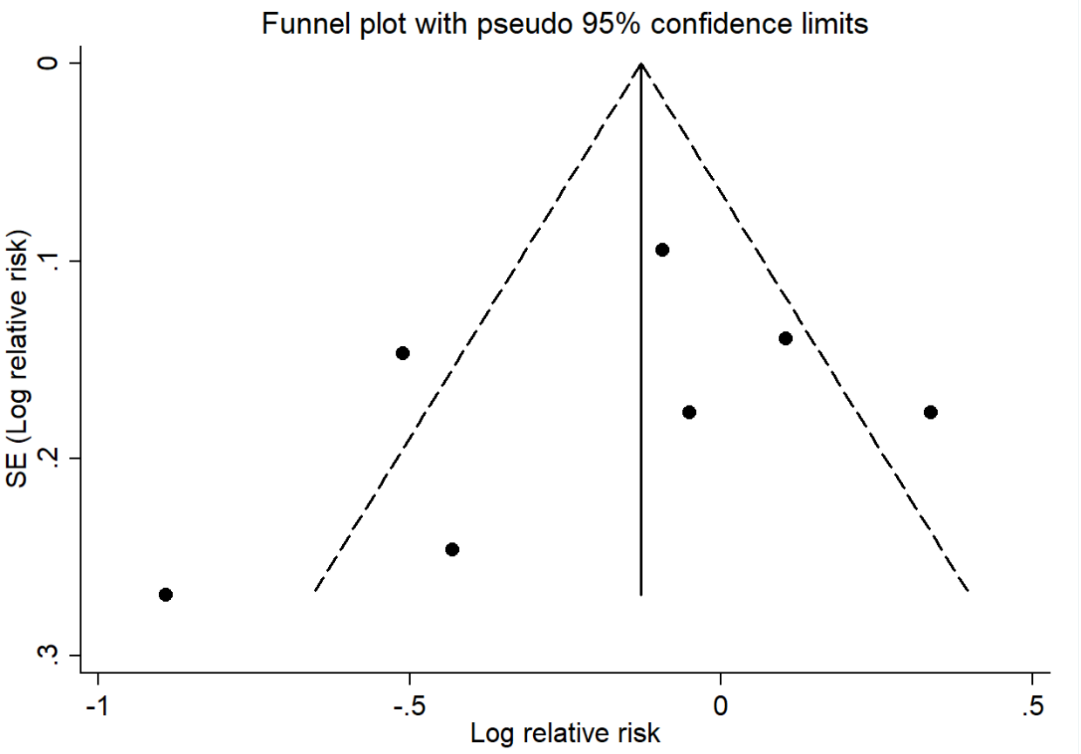

The vertical line represents the pooled RR. The dashed lines represent the pseudo-95% confidence interval of the RR. The circles represent risk estimates for each cohort, and the horizontal line represents standard errors of the RR. RR, relative risk.

Figure S44. Funnel plot of relative risk (RR) for type 2 diabetes comparing the lowest with the highest categories for lignoceric acid (24:0) biomarker level.

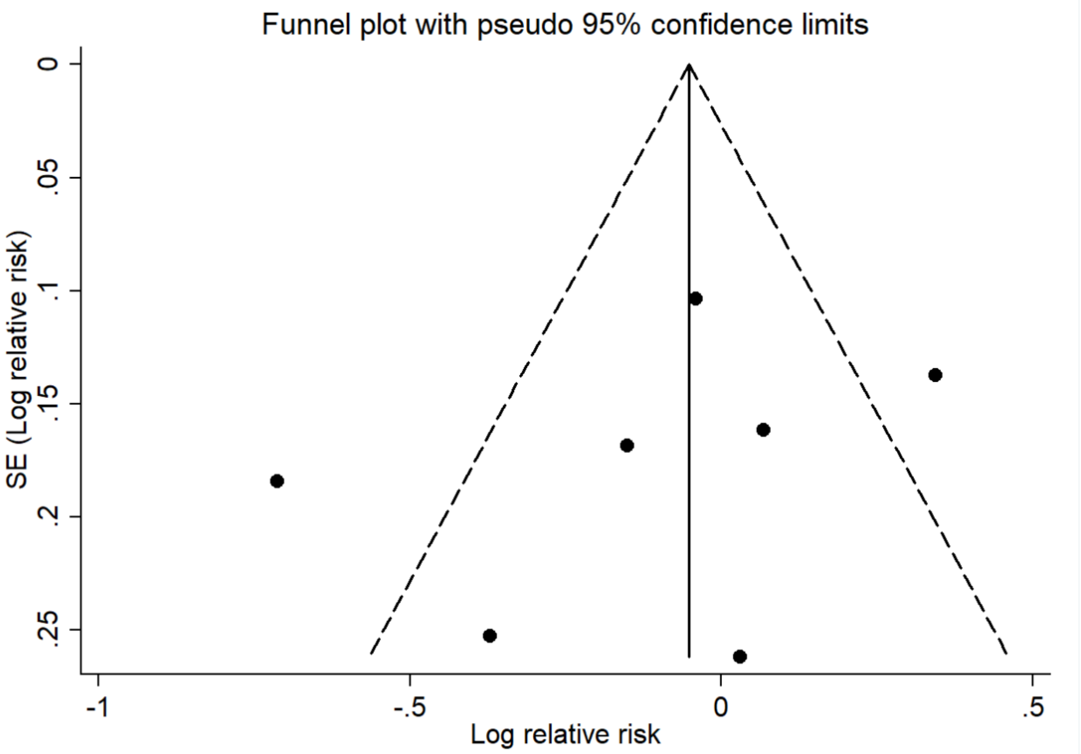

The vertical line represents the pooled RR. The dashed lines represent the pseudo-95% confidence interval of the RR. The circles represent risk estimates for each cohort, and the horizontal line represents standard errors of the RR. RR, relative risk.

Figure S45. Funnel plot of relative risk (RR) for cardiovascular disease comparing the lowest with the highest categories for total saturated fatty acid biomarker level.

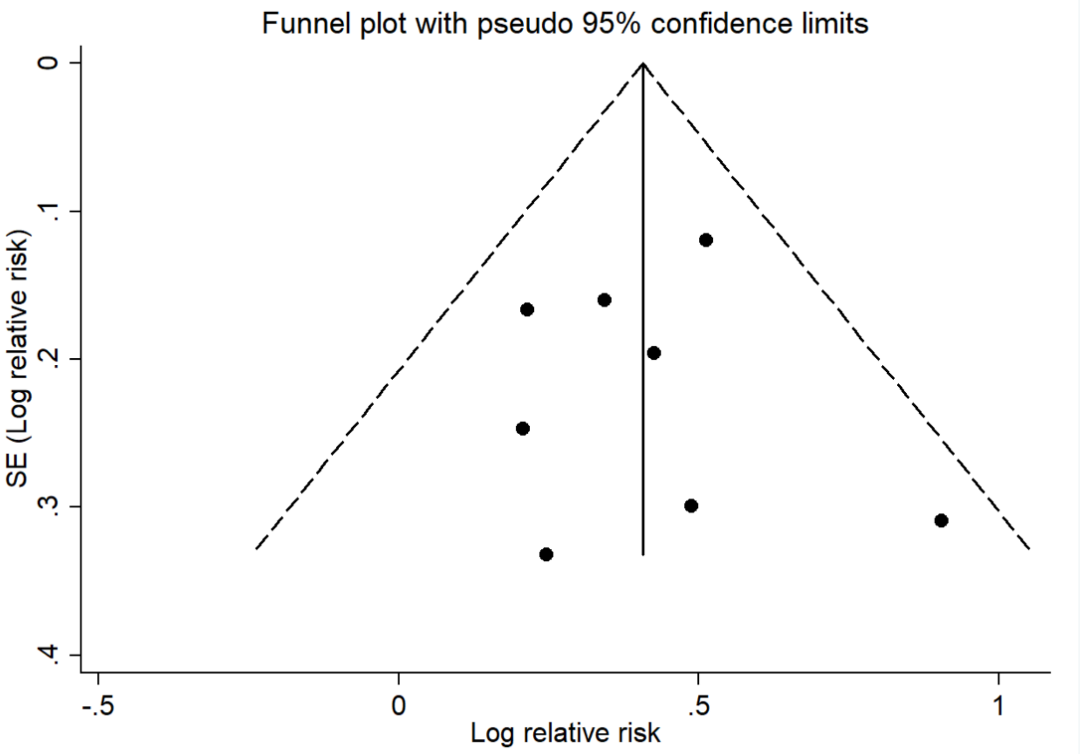

The vertical line represents the pooled RR. The dashed lines represent the pseudo-95% confidence interval of the RR. The circles represent risk estimates for each cohort, and the horizontal line represents standard errors of the RR. RR, relative risk.

Figure S46. Funnel plot of relative risk (RR) for cardiovascular disease comparing the lowest with the highest categories for myristic acid (14:0) biomarker level.

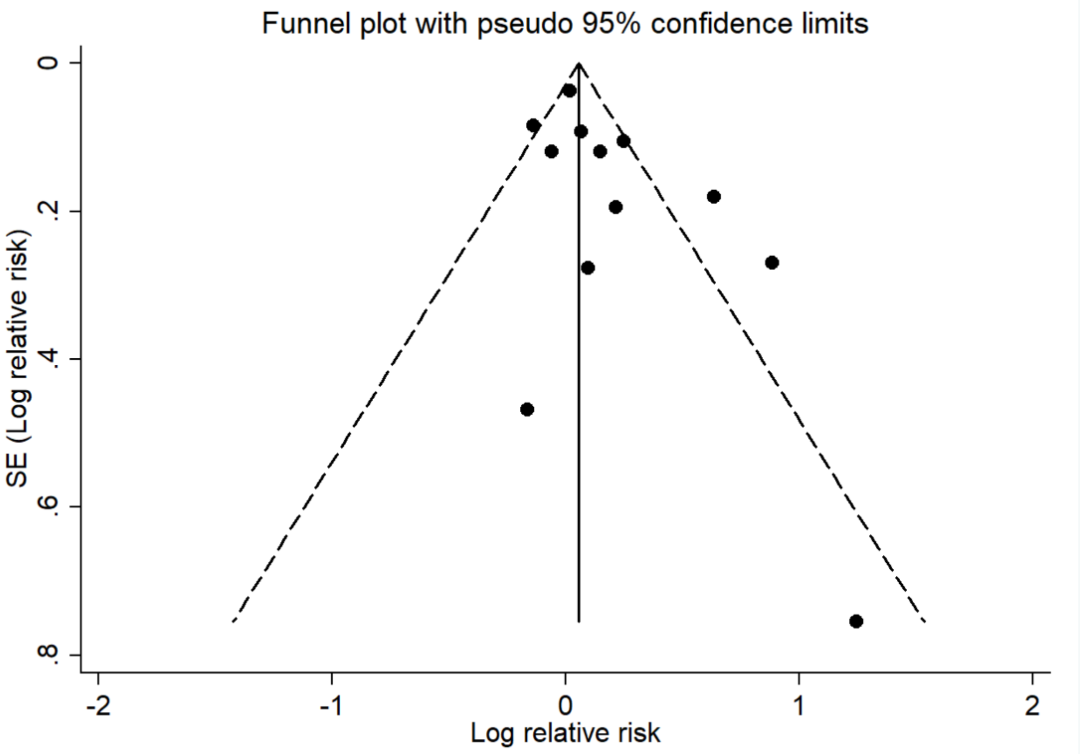

The vertical line represents the pooled RR. The dashed lines represent the pseudo-95% confidence interval of the RR. The circles represent risk estimates for each cohort, and the horizontal line represents standard errors of the RR. RR, relative risk.

Figure S47. Funnel plot of relative risk (RR) for cardiovascular disease comparing the lowest with the highest categories for palmitic acid (16:0) biomarker level.

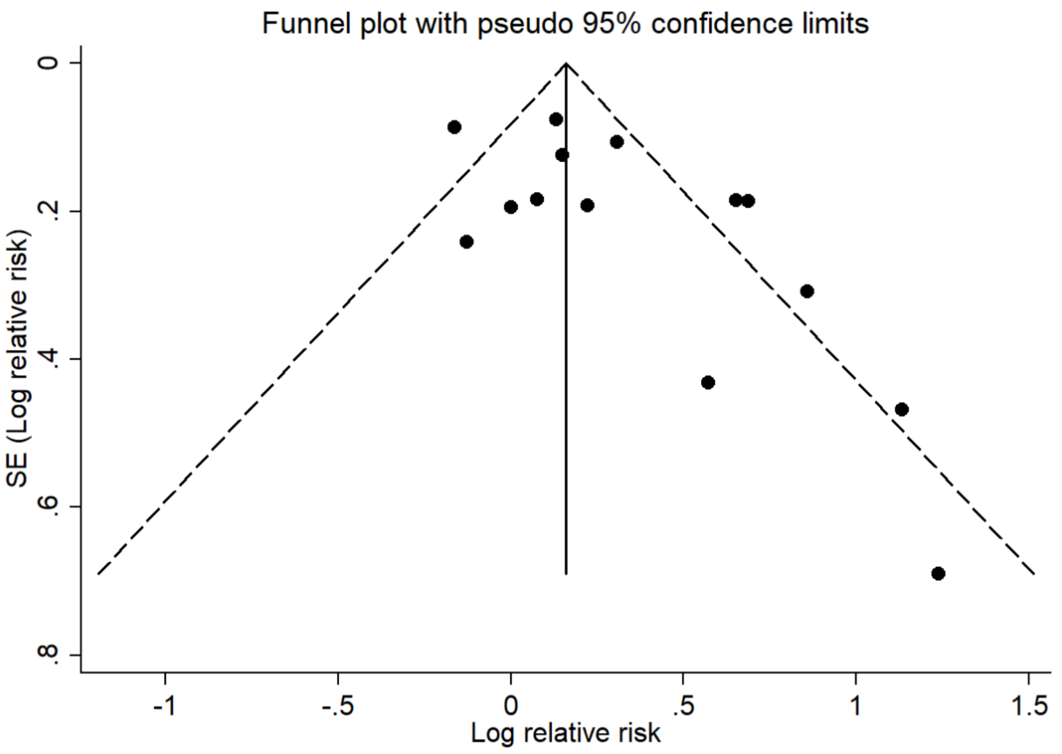

The vertical line represents the pooled RR. The dashed lines represent the pseudo-95% confidence interval of the RR. The circles represent risk estimates for each cohort, and the horizontal line represents standard errors of the RR. RR, relative risk.

Figure S48. Funnel plot of relative risk (RR) for cardiovascular disease comparing the lowest with the highest categories for sum of pentadecanoic acid (15:0) and margaric acid (17:0) biomarker level.

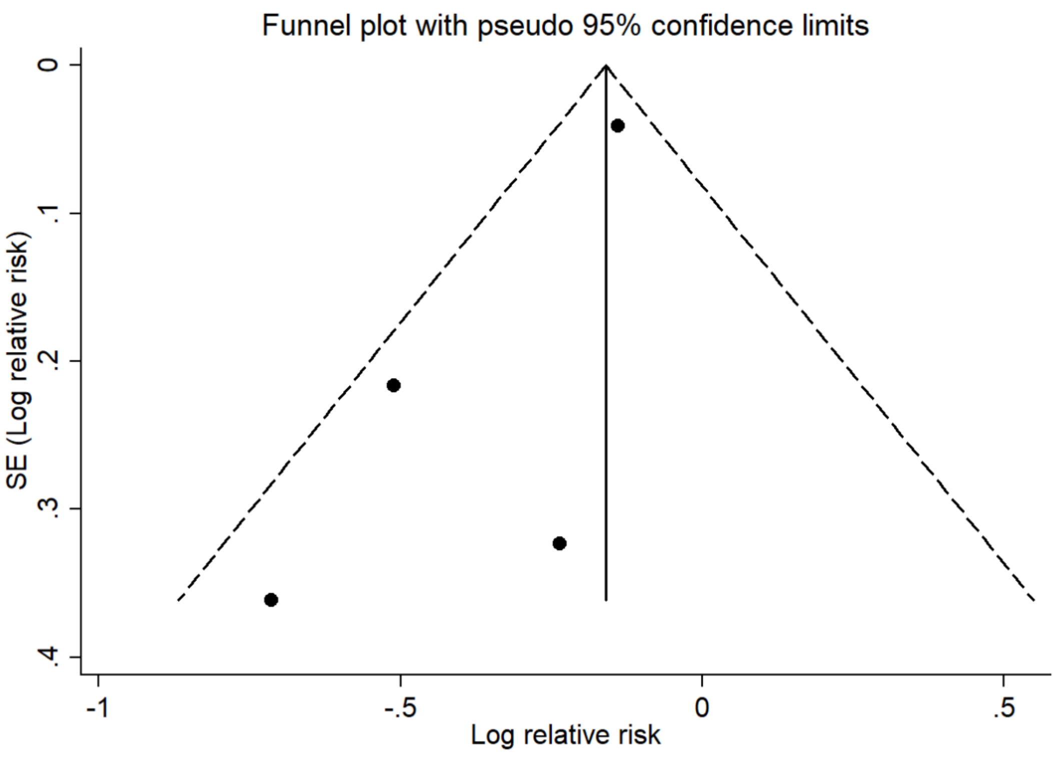

The vertical line represents the pooled RR. The dashed lines represent the pseudo-95% confidence interval of the RR. The circles represent risk estimates for each cohort, and the horizontal line represents standard errors of the RR. RR, relative risk.

Figure S49. Funnel plot of relative risk (RR) for cardiovascular disease comparing the lowest with the highest categories for lignoceric acid (24:0) biomarker level.

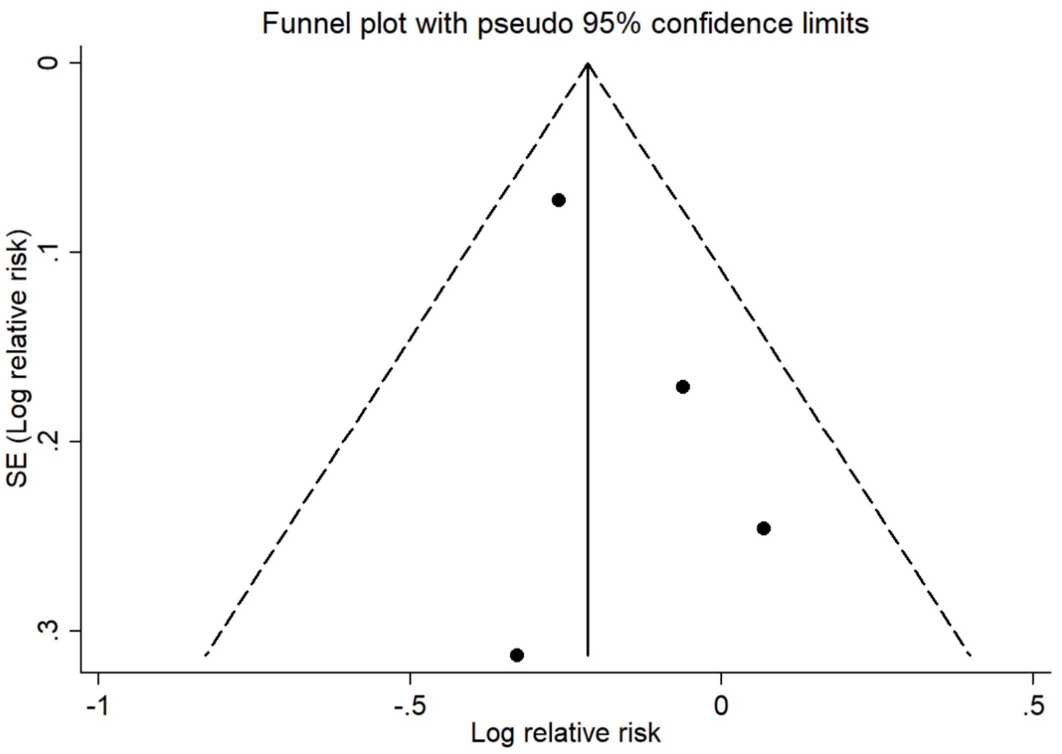

The vertical line represents the pooled RR. The dashed lines represent the pseudo-95% confidence interval of the RR. The circles represent risk estimates for each cohort, and the horizontal line represents standard errors of the RR. RR, relative risk.

Figure S50. Funnel plot of relative risk (RR) for cardiovascular disease comparing the lowest with the highest categories for pentadecanoic acid (15:0) biomarker level.

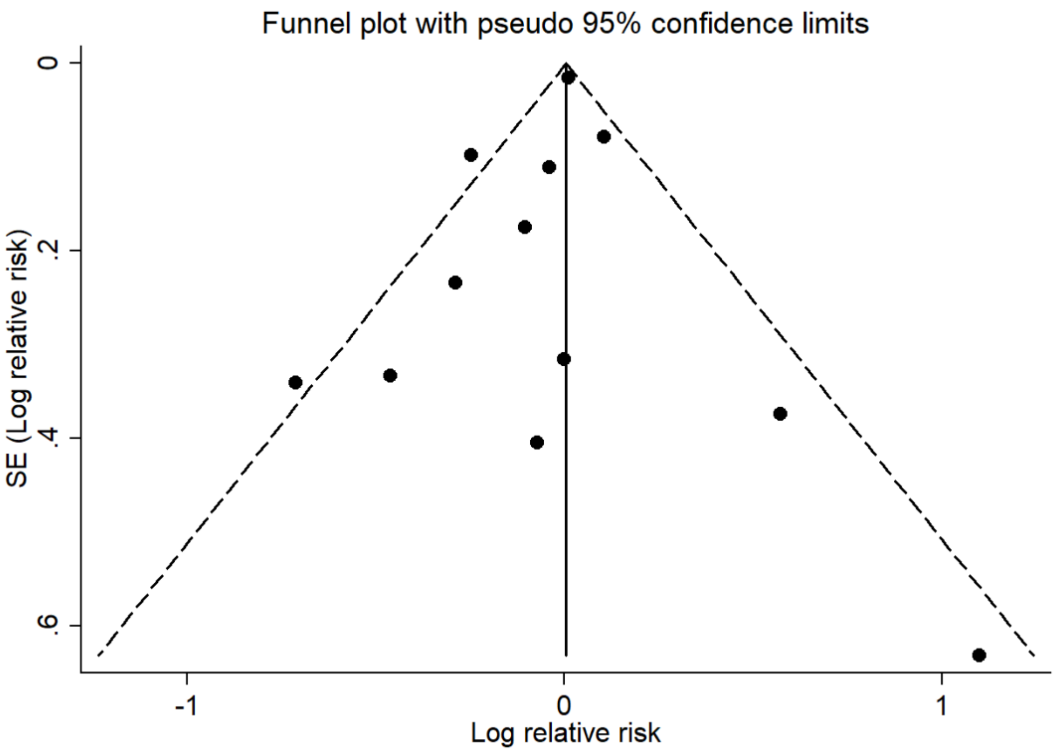

The vertical line represents the pooled RR. The dashed lines represent the pseudo-95% confidence interval of the RR. The circles represent risk estimates for each cohort, and the horizontal line represents standard errors of the RR. RR, relative risk.

Figure S51. Funnel plot of relative risk (RR) for cardiovascular disease comparing the lowest with the highest categories for margaric acid (17:0) biomarker level.

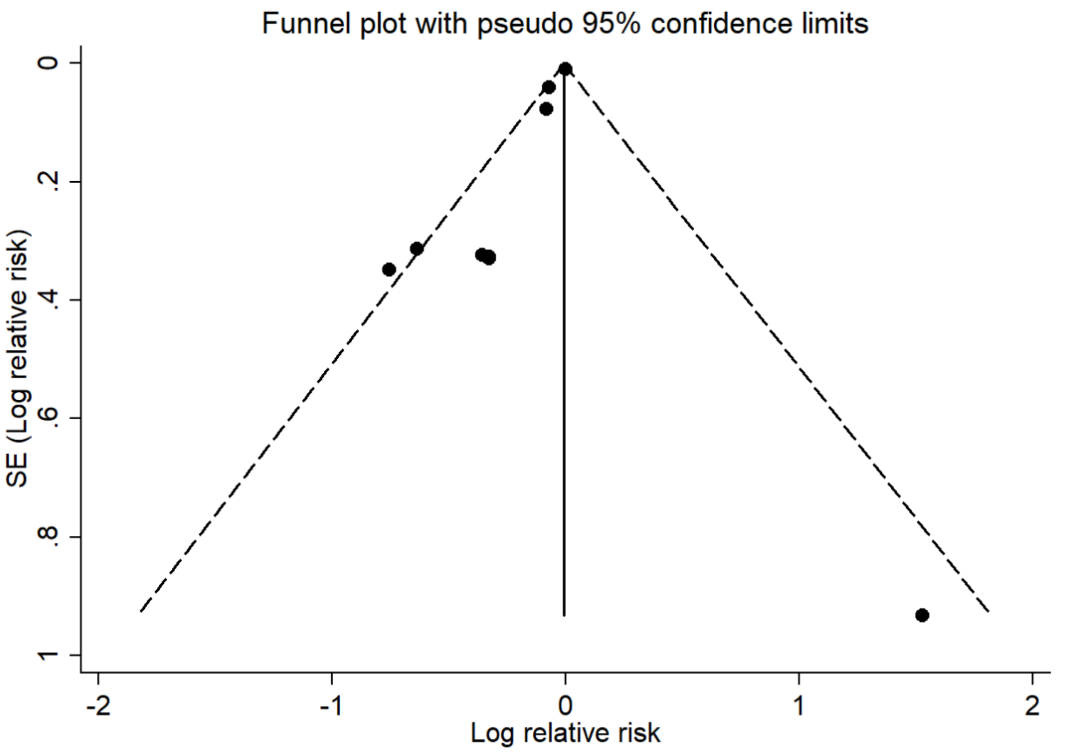

The vertical line represents the pooled RR. The dashed lines represent the pseudo-95% confidence interval of the RR. The circles represent risk estimates for each cohort, and the horizontal line represents standard errors of the RR. RR, relative risk.

Figure S52. Funnel plot of relative risk (RR) for cardiovascular disease comparing the lowest with the highest categories for stearic acid (18:0) biomarker level.

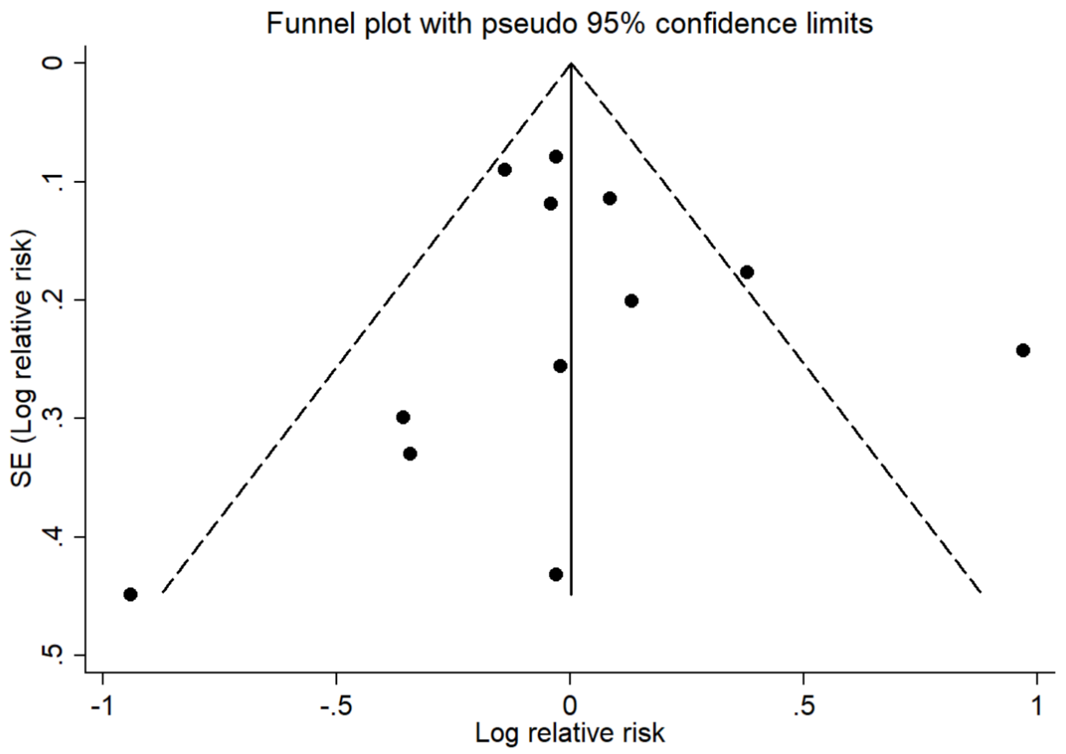

The vertical line represents the pooled RR. The dashed lines represent the pseudo-95% confidence interval of the RR. The circles represent risk estimates for each cohort, and the horizontal line represents standard errors of the RR. RR, relative risk.

Figure S53. Funnel plot of relative risk (RR) for cardiovascular disease comparing the lowest with the highest categories for arachidic acid (20:0) biomarker level.

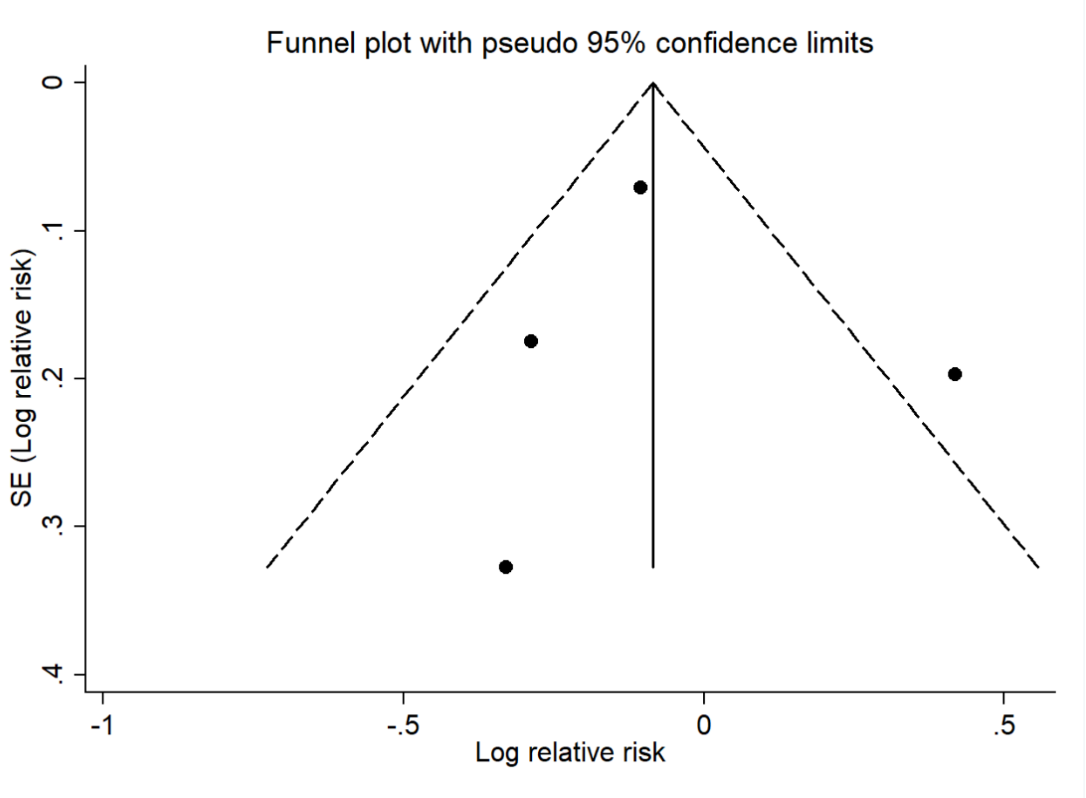

The vertical line represents the pooled RR. The dashed lines represent the pseudo-95% confidence interval of the RR. The circles represent risk estimates for each cohort, and the horizontal line represents standard errors of the RR. RR, relative risk.

Figure S54. Funnel plot of relative risk (RR) for cardiovascular disease comparing the lowest with the highest categories for behenic acid (22:0) biomarker level.

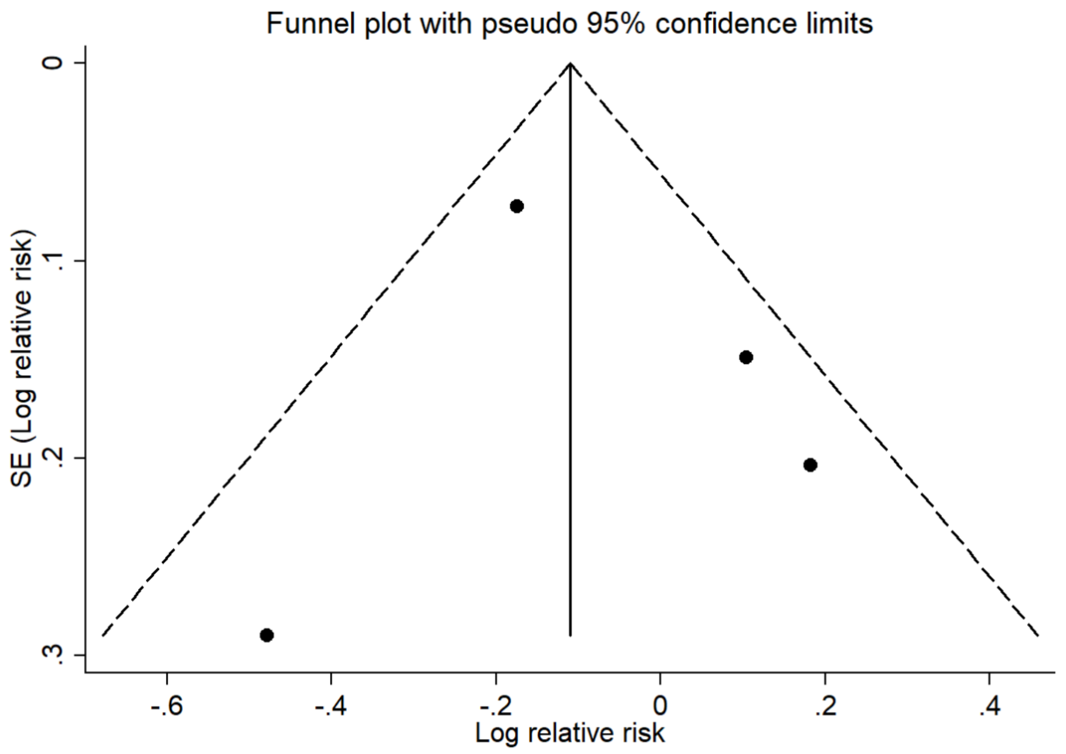

The vertical line represents the pooled RR. The dashed lines represent the pseudo-95% confidence interval of the RR. The circles represent risk estimates for each cohort, and the horizontal line represents standard errors of the RR. RR, relative risk.

Figure S55. Funnel plot of relative risk (RR) for coronary heart disease comparing the lowest with the highest categories for total saturated fatty acid biomarker level.

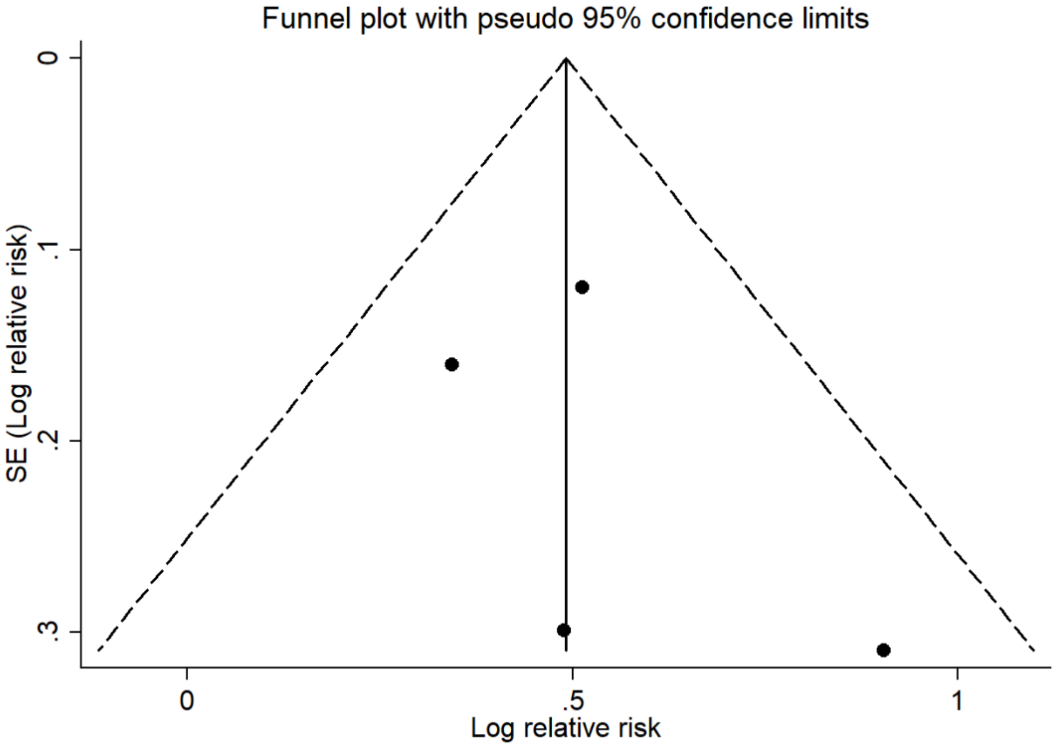

The vertical line represents the pooled RR. The dashed lines represent the pseudo-95% confidence interval of the RR. The circles represent risk estimates for each cohort, and the horizontal line represents standard errors of the RR. RR, relative risk.

Figure S56. Funnel plot of relative risk (RR) for coronary heart disease comparing the lowest with the highest categories for palmitic acid (16:0) biomarker level.

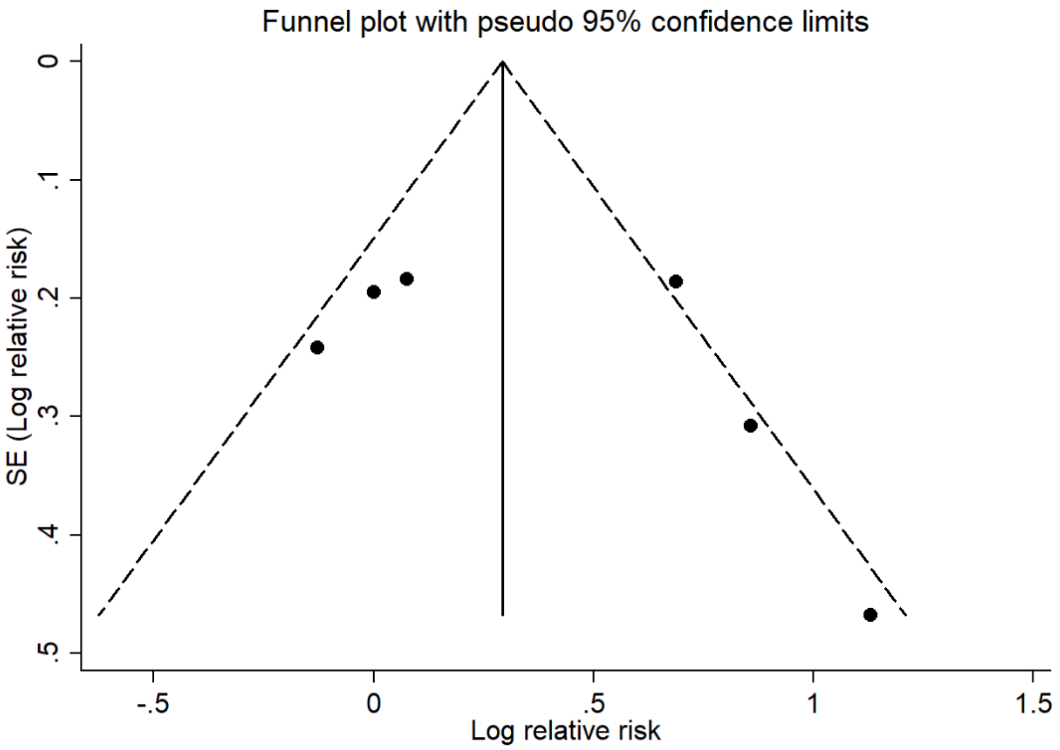

The vertical line represents the pooled RR. The dashed lines represent the pseudo-95% confidence interval of the RR. The circles represent risk estimates for each cohort, and the horizontal line represents standard errors of the RR. RR, relative risk.

Figure S57. Funnel plot of relative risk (RR) for coronary heart disease comparing the lowest with the highest categories for pentadecanoic acid (15:0) biomarker level.

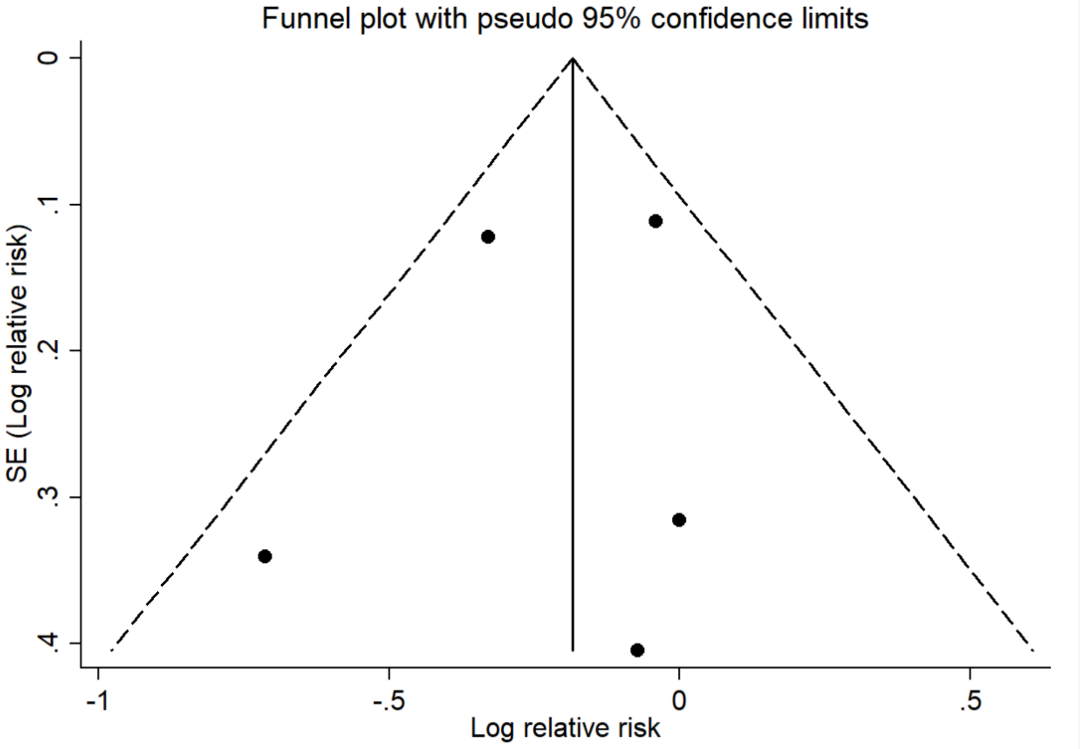

The vertical line represents the pooled RR. The dashed lines represent the pseudo-95% confidence interval of the RR. The circles represent risk estimates for each cohort, and the horizontal line represents standard errors of the RR. RR, relative risk.

Figure S58. Funnel plot of relative risk (RR) for coronary heart disease comparing the lowest with the highest categories for margaric acid (17:0) biomarker level.

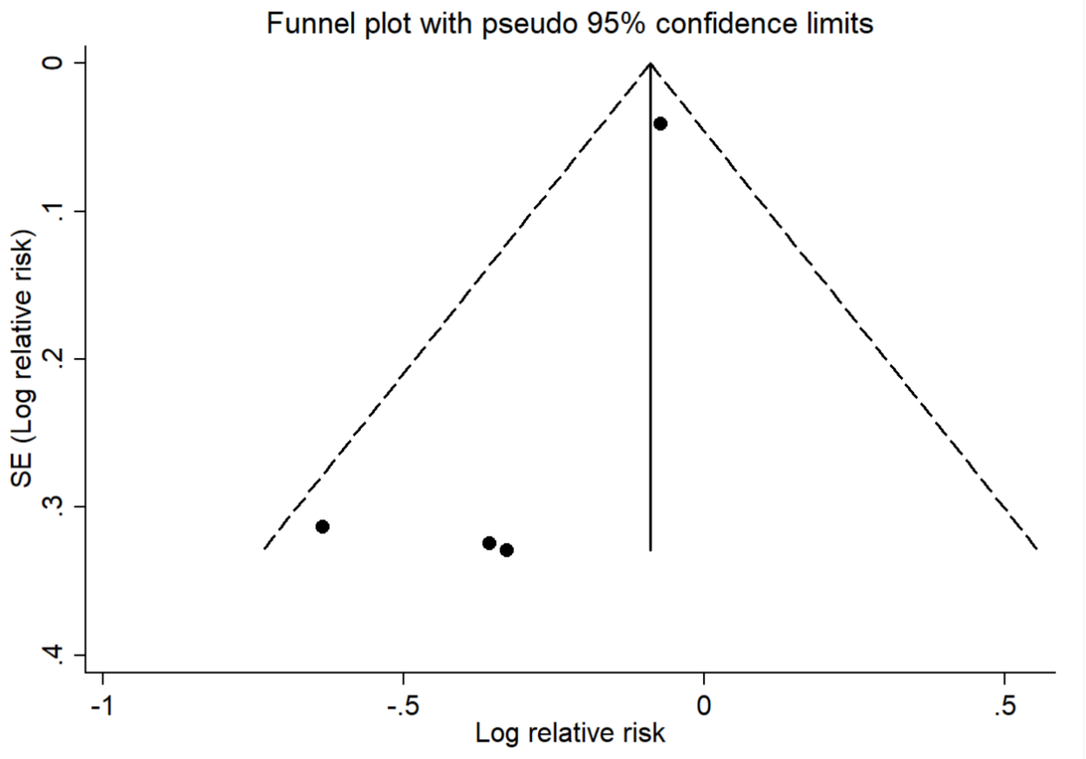

The vertical line represents the pooled RR. The dashed lines represent the pseudo-95% confidence interval of the RR. The circles represent risk estimates for each cohort, and the horizontal line represents standard errors of the RR. RR, relative risk.

Figure S59. Funnel plot of relative risk (RR) for coronary heart disease comparing the lowest with the highest categories for sum of pentadecanoic acid (15:0) and margaric acid (17:0) biomarker level.

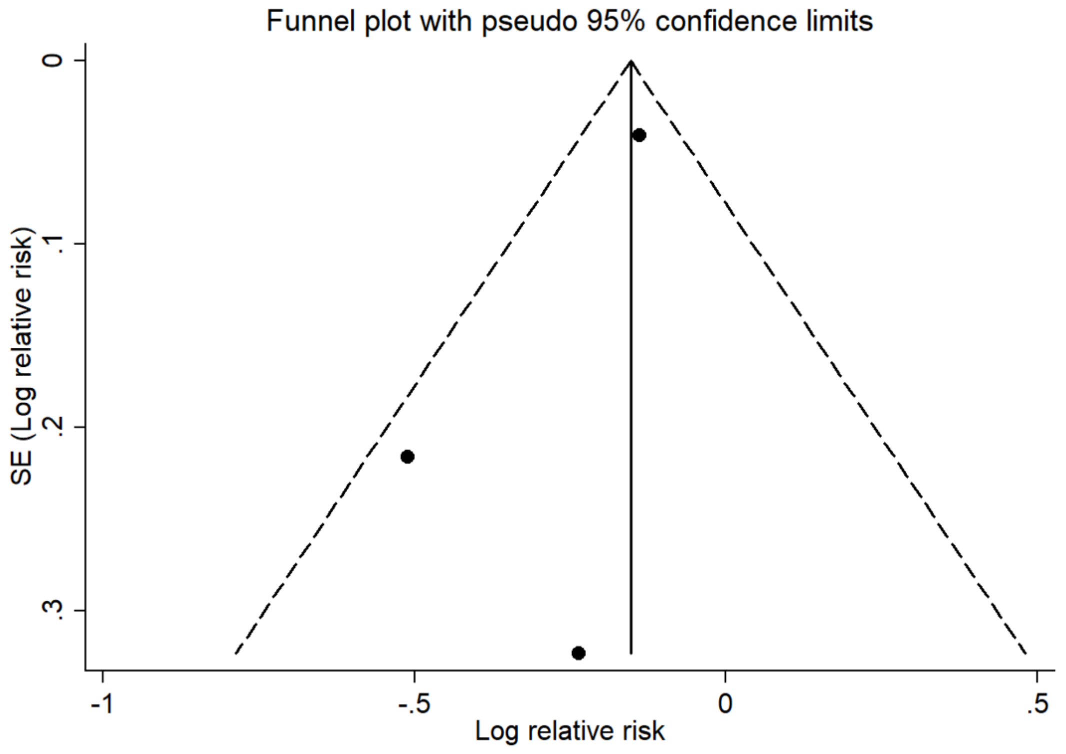

The vertical line represents the pooled RR. The dashed lines represent the pseudo-95% confidence interval of the RR. The circles represent risk estimates for each cohort, and the horizontal line represents standard errors of the RR. RR, relative risk.

Figure S60. Funnel plot of relative risk (RR) for coronary heart disease comparing the lowest with the highest categories for myristic acid (14:0) biomarker level.

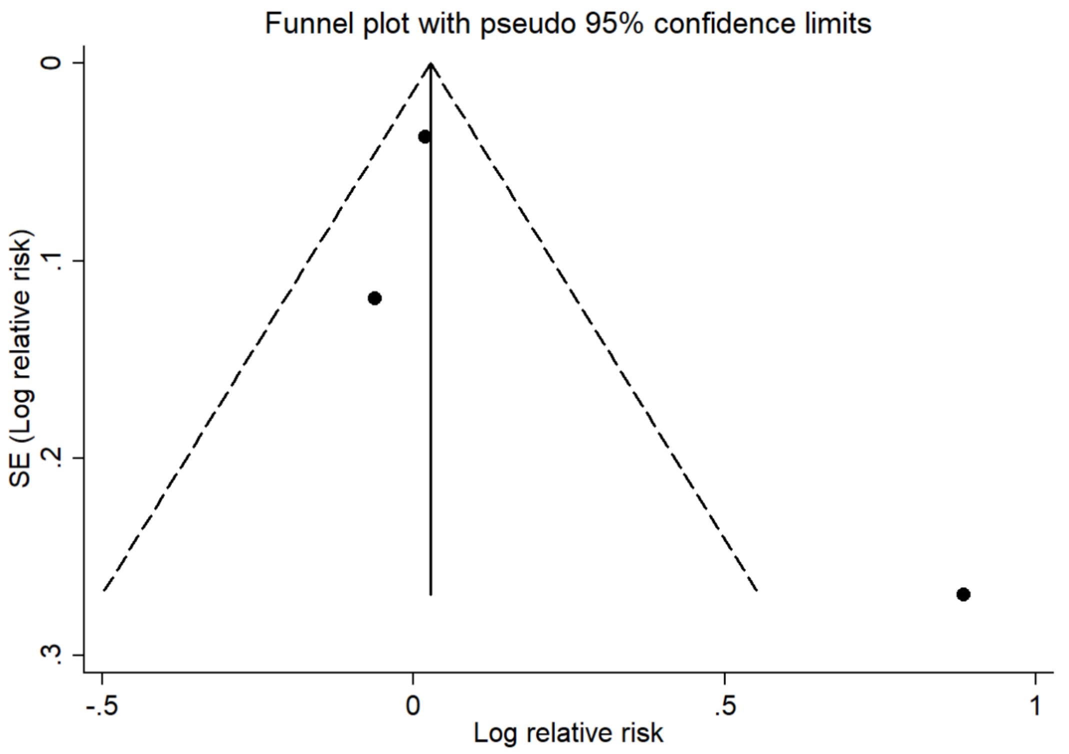

The vertical line represents the pooled RR. The dashed lines represent the pseudo-95% confidence interval of the RR. The circles represent risk estimates for each cohort, and the horizontal line represents standard errors of the RR. RR, relative risk.

Figure S61. Funnel plot of relative risk (RR) for coronary heart disease comparing the lowest with the highest categories for stearic acid (18:0) biomarker level.

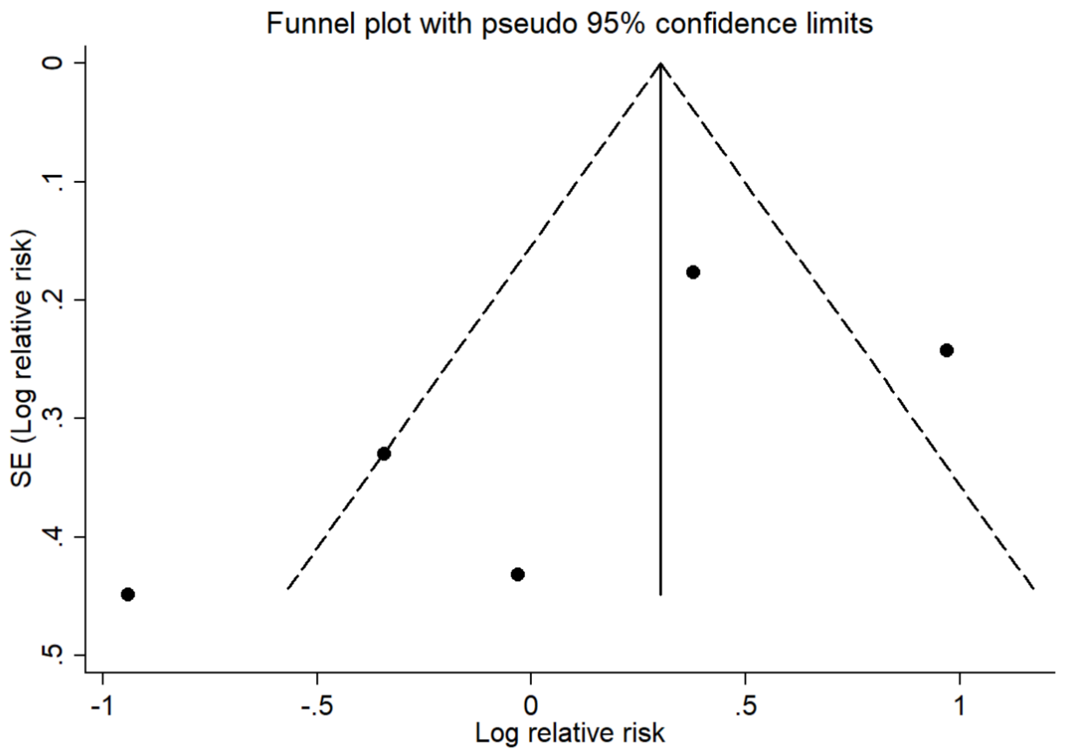

The vertical line represents the pooled RR. The dashed lines represent the pseudo-95% confidence interval of the RR. The circles represent risk estimates for each cohort, and the horizontal line represents standard errors of the RR. RR, relative risk.

Figure S62. Funnel plot of relative risk (RR) for stroke comparing the lowest with the highest categories for total saturated fatty acid biomarker level.

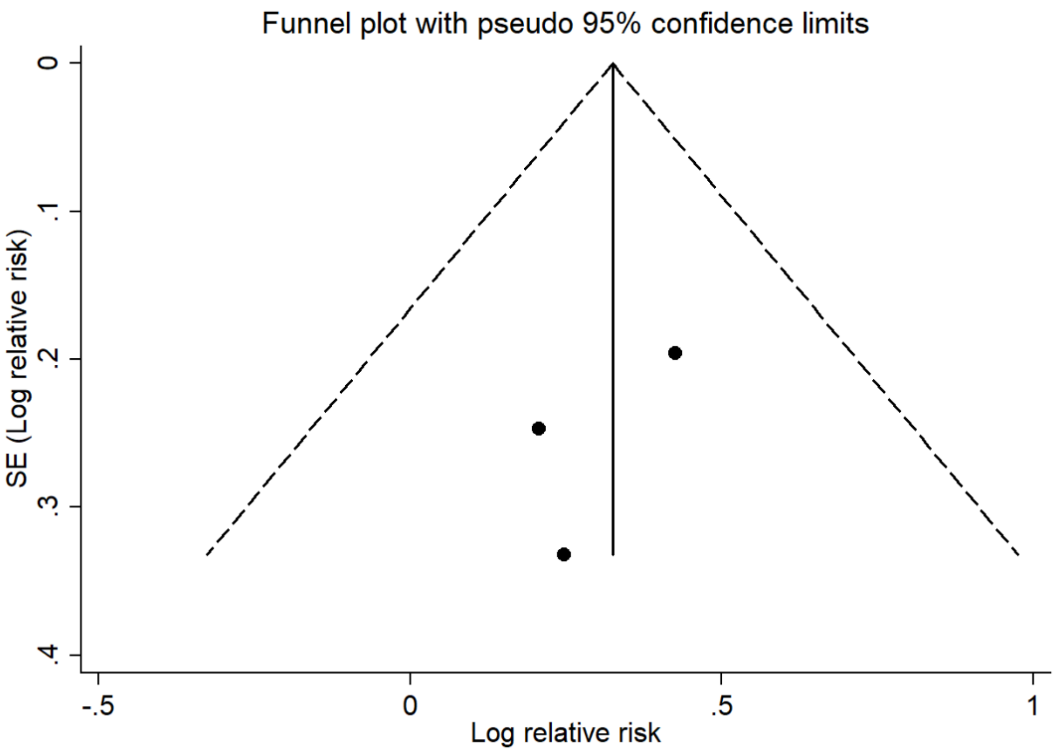

The vertical line represents the pooled RR. The dashed lines represent the pseudo-95% confidence interval of the RR. The circles represent risk estimates for each cohort, and the horizontal line represents standard errors of the RR. RR, relative risk.

Figure S63. Funnel plot of relative risk (RR) for stroke comparing the lowest with the highest categories for myristic acid (14:0) biomarker level.

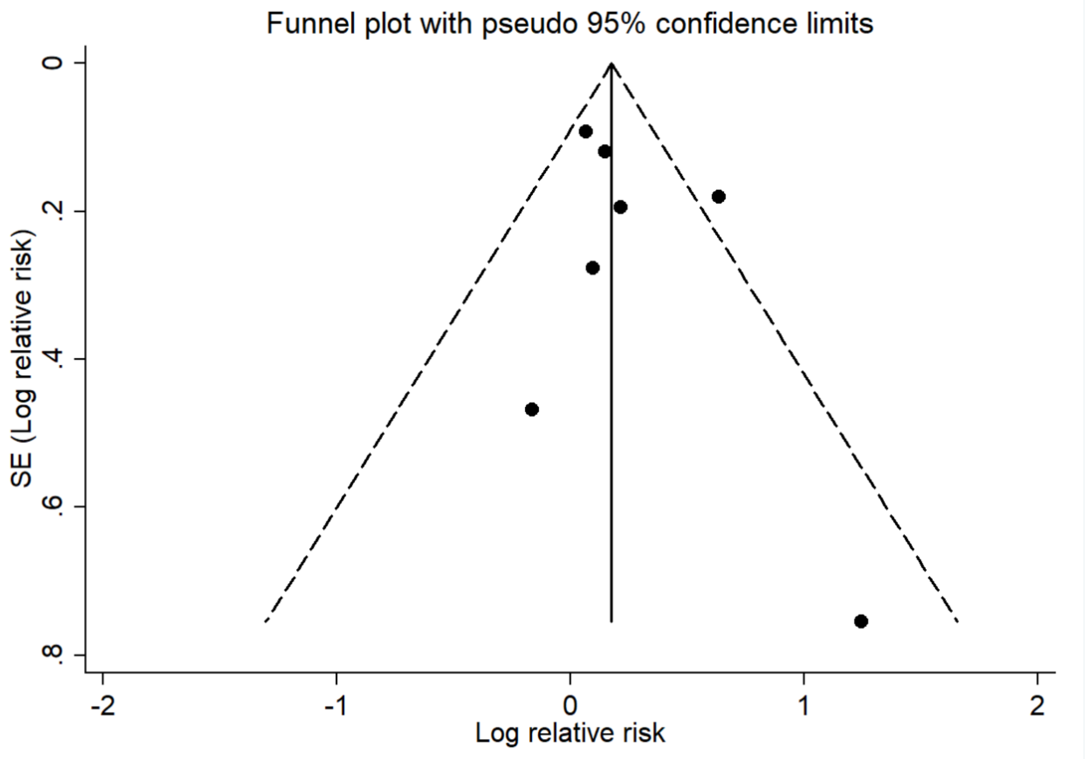

The vertical line represents the pooled RR. The dashed lines represent the pseudo-95% confidence interval of the RR. The circles represent risk estimates for each cohort, and the horizontal line represents standard errors of the RR. RR, relative risk.

Figure S64. Funnel plot of relative risk (RR) for stroke comparing the lowest with the highest categories for palmitic acid (16:0) biomarker level.

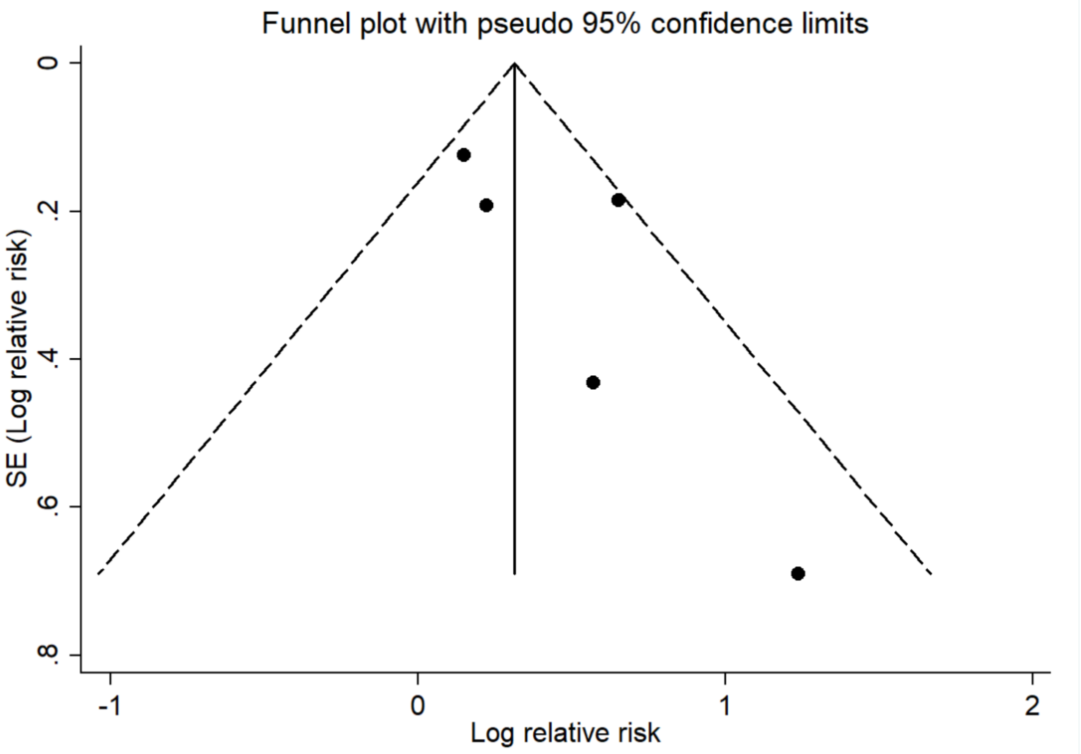

The vertical line represents the pooled RR. The dashed lines represent the pseudo-95% confidence interval of the RR. The circles represent risk estimates for each cohort, and the horizontal line represents standard errors of the RR. RR, relative risk.

Figure S65. Funnel plot of relative risk (RR) for stroke comparing the lowest with the highest categories for pentadecanoic acid (15:0) biomarker level.

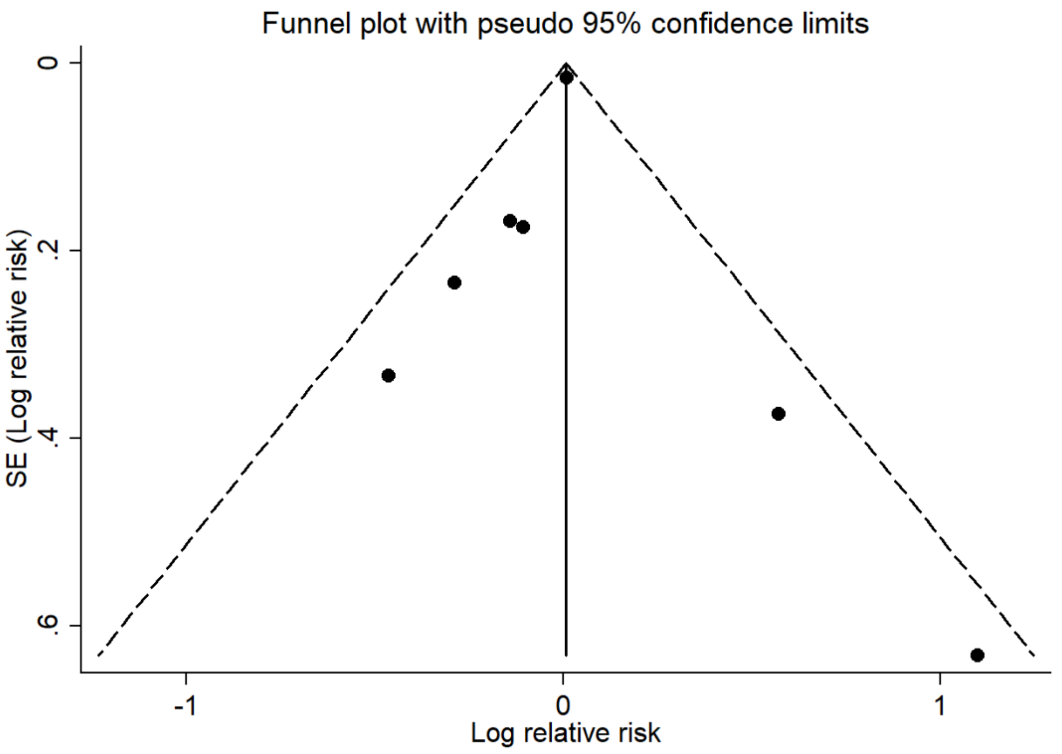

The vertical line represents the pooled RR. The dashed lines represent the pseudo-95% confidence interval of the RR. The circles represent risk estimates for each cohort, and the horizontal line represents standard errors of the RR. RR, relative risk.

Figure S66. Funnel plot of relative risk (RR) for stroke comparing the lowest with the highest categories for margaric acid (17:0) biomarker level.

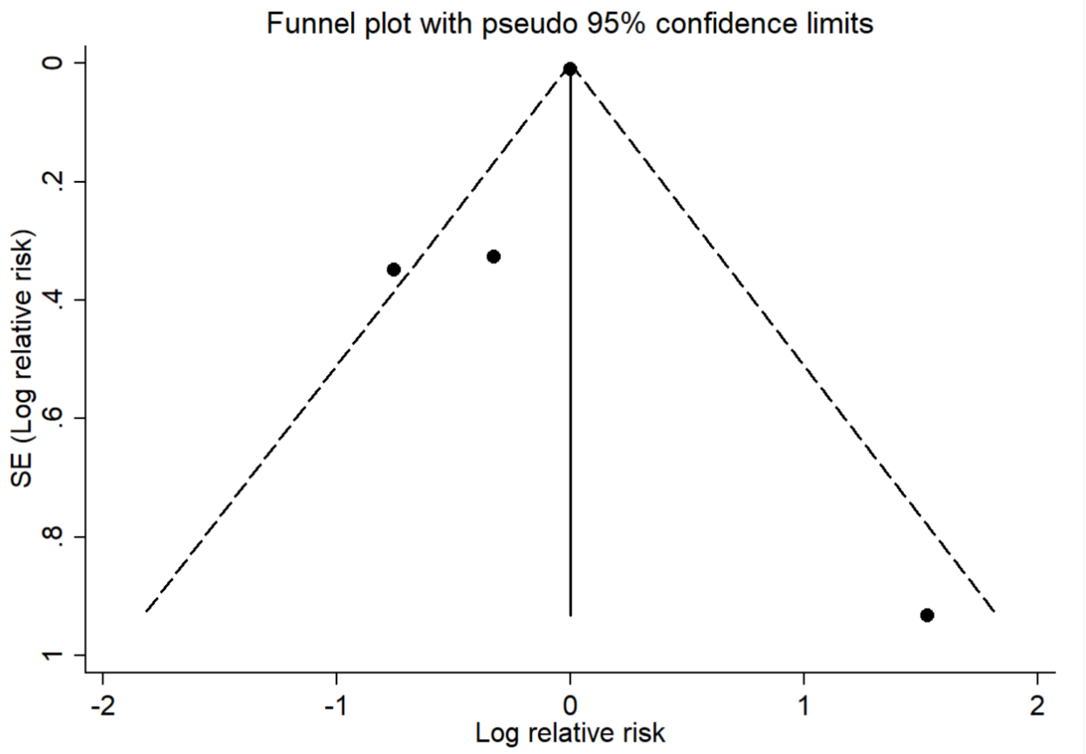

The vertical line represents the pooled RR. The dashed lines represent the pseudo-95% confidence interval of the RR. The circles represent risk estimates for each cohort, and the horizontal line represents standard errors of the RR. RR, relative risk.

Figure S67. Funnel plot of relative risk (RR) for stroke comparing the lowest with the highest categories for stearic acid (18:0) biomarker level.

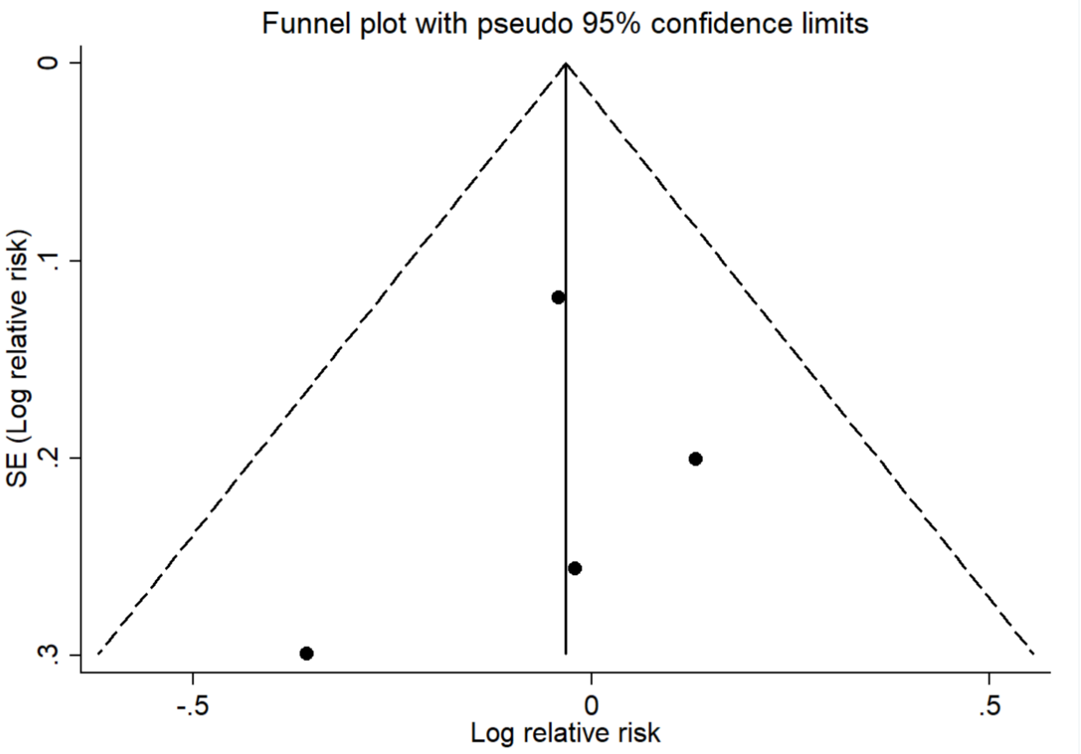

The vertical line represents the pooled RR. The dashed lines represent the pseudo-95% confidence interval of the RR. The circles represent risk estimates for each cohort, and the horizontal line represents standard errors of the RR. RR, relative risk.
