# Supplementary material for: Saturated fatty acid biomarkers and risk of cardiometabolic diseases: A meta-analysis of prospective studies
Source: Front Nutr. 2022 Aug 15;9:963471. doi: 10.3389/fnut.2022.963471 (PMC9421298; doi:10.3389/fnut.2022.963471)
Supplement: Supplementary file 2 [file Table_1.pdf]

Table S1. Summary of prospective studies on saturated fatty acid biomarkers and cardiometabolic diseases

Author, Publication, year, country	Study design	Follow up (year)	Study name	No of participants	Age range (year)	Men (%)	Assay method	Biological sample	Lipid fraction measured	Exposure	Ascertainment method	No of Cases	Adjustment for confounding factors	Study quality*
Laaksonen et al, 2002, Finland[27]	PC	4.0	KIHD	895	42-60	100.0	GC	Serum	Total fatty acid fraction	Total SFAs, 16:0	Records	34 incident T2D	Age, smoking, alcohol drinking, BMI, antihypertensive medications use, DBP, blood lipid, concentrations of glucose and insulin, exercise energy expenditure	High
Wang et al, 2003, USA[28]	PC	9.0	ARIC	2,909	45-64	46.0	GLC	Plasma	Total fatty acid fraction	Total SFAs, 16:0, 18:0	Serum glucose, records	252 incident T2D	Age, sex, education, smoking, alcohol drinking, BMI, WHR, physical activity, family history of diabetes	High
Hodge et al, 2007, Australia[29]	PCC	4.0	MCCS	3,737	36-72	44.1	GLC	Plasma	Phospholipid fraction	Total SFAs, 15:0, 16:0, 18:0	Self-report	346 incident T2D	Age, sex, alcohol drinking, BMI, physical activity, country of birth, WHR, family history of diabetes	High
Krachler et al, 2008, Sweden[30]	NCC	8.8	VIP	450	30-60	NR	GLC	Erythrocyte membrane	Phospholipid fraction	Total SFAs, 14:0, 15:0, 16:0, 17:0, 18:0	Records	159 incident T2D	Age, sex, smoking, alcohol drinking, BMI, physical activity, HbA1c, EMFA	High
Mozaffarian et al, 2010, USA[31]	PC	10.0	CHS	3,736	≥65	42.0	GC	Plasma	Phospholipid fraction	15:0, 17:0	Medication use, blood glucose	304 incident T2D	Age, sex, education, smoking, alcohol drinking, physical activity, race, enrollment site, waist circumference, coronary heart disease, consumption of carbohydrate, protein, red meat, whole-fat dairy foods, low-fat dairy foods, total energy	High
Patel et al, 2010, UK[32]	NCC	10.0	EPIC-Norfolk	383	40-79	53.3	GC	Erythrocyte membrane	Phospholipid fraction	Total SFAs, 14:0, 15:0, 16:0, 17:0, 18:0	Self-report, medication use	199 incident T2D	Age, sex, smoking, alcohol drinking, BMI, physical activity, family history of diabetes	High
Kröger et al, 2011, Europe[33]	NCC	7.0 (mean)	EPIC-Postsdam	2,724	23-71	44.5	GC	Erythrocyte membrane	Phospholipid fraction	Total SFAs, 14:0, 15:0, 16:0, 17:0, 18:0, 20:0, 22:0, 24:0	Self-report, records, medication use	673 incident T2D	Age, sex, education, smoking, alcohol drinking, BMI, physical activity, waist circumference, occupational activity, dietary factors	High

Mozaffarian et al, 2013, USA[34]	PC	7.0	MESA	2,281	45-84	46.7	GC	Plasma	Phospholipid fraction	14:0, 15:0	Serum glucose, medication use	205 incident T2D	Age, sex, education, smoking, alcohol drinking, BMI, physical activity, race-ethnicity, field center, waist circumference, dietary consumption of whole-fat dairy foods, low-fat dairy foods, red meat, total energy	High
Zong et al, 2013,China[35]	PC	6.0	NHAPC	2,066	50-70	45.5	GC	Erythrocyte membrane	Phospholipid fraction	16:0	Serum glucose, medication use	496 incident T2D	Age, sex, education, smoking, alcohol drinking, BMI, physical activity, region, residence, family history of diabetes, total energy intake, percentage of energy intake from carbohydrate, energy-adjusted dietary	High
Forouhi et al, 2014, Europe[36]	PCC	17.0	EPIC-InterAct	27,296	23-71	NR	GC	Plasma	Phospholipid fraction	14:0, 15:0, 16:0, 17:0, 18:0, 20:0, 22:0, 24:0	Self-report, records, medication use	12,132 incident T2D	Age, sex, education, smoking, alcohol drinking, BMI, physical activity, total energy intake, dietary factors	High
Lemaitre et al, 2015, USA[37]	PC	10.0	CHS	3,179	≥65	38.6	GC	Plasma	Phospholipid fraction	20:0, 22:0, 24:0	Medication use, blood glucose	284 incident T2D	Age, sex, education, smoking, alcohol drinking, BMI, physical activity, race, clinic site, waist circumference, treated hypertension, prevalent IHD, self-reported health status at baseline, plasma phospholipid 16:0 and triglycerides at baseline	High
Ma et al, 2015, USA[38]	PC	9.0 (midpoint)	CHS	3,004	≥65	40.0	GC	Plasma	Phospholipid fraction	14:0, 16:0, 18:0	Medication use, blood glucose	297 incident T2D	Age, sex, education, smoking, alcohol drinking, BMI, physical activity, race, clinic site, IHD, hypertension, waist circumference, total energy intake, dietary factors	High
Harris et al, 2016, USA[39]	PC	11.0 (median)	WHIMS	6,379	65-80	0.0	GC	Erythrocyte membrane	Phospholipid fraction	14:0, 16:0, 18:0, 20:0, 22:0, 24:0	Self-report	703 incident T2D	Age, education, smoking, alcohol drinking, physical activity, race, waist girth, dietary glycemic load, family history of diabetes	High
Yakoob et al, 2016, USA[40]	NCC	15.2 (mean)	NHS	1,864	44-70	0.0	GLC	Erythrocyte membrane	Phospholipid fraction	14:0, 15:0, 17:0	Self-report	184 incident T2D	Age, smoking, alcohol drinking, physical activity, race, MI, hypercholesterolemia, hypertension, family history of diabetes, menopausal status, postmenopausal hormone use, dietary factors, plasma 18:1t, 18:2t,16:0, and 18:0	High

Yakoob et al, 2016, USA[40]	NCC	15.2 (mean)	HPFS	1,469	48-83	100.0	GLC	Erythrocyte membrane	Phospholipid fraction	14:0, 15:0, 17:0	Self-report	93 incident T2D	Age, smoking, alcohol drinking, physical activity, race, MI, hypercholesterolemia, hypertension, family history of diabetes, menopausal status, postmenopausal hormone use, dietary factors, plasma 18:1t, 18:2t,16:0, and 18:0	High
Akter et al, 2017, Japan[41]	NCC	5.0	HHS	1,014	34-69	90.9	GC	Serum	Phospholipid fraction	Total SFAs, 14:0, 15:0, 16:0, 17:0, 18:0, 20:0	plasma glucose, glycated hemoglobin , self-report	336 incident T2D	Age, sex, smoking, alcohol drinking, BMI, physical activity, month of examination, shift work, sleep duration, family history of diabetes and hypertension	High
Lin et al, 2018, China[42]	PC	5.6 (median)	GNHS	2,683	40-75	30.3	GC	Erythrocyte membrane	Phospholipid fraction	Total SFAs,14:0, 16:0, 18:0, 20:0, 22:0, 24:0	Fasting glucose, self-report	216 incident T2D	Age, sex, education, smoking, alcohol drinking, BMI, physical activity, WHR, tea drinking, household income, family history of diabetes, total energy intake, blood lipid, glycemic markers	High
Lu et al, 2018, Singapore[43]	NCC	6.0	SCHS	320	45-74	49.4	GC	Serum	Total fatty acid fraction	14:0, 16:0, 18:0	HbA1c	160 incident T2D	Age, sex, smoking, BMI, physical activity, date of blood collection, hypertension, fasting status, triglycerides, HDL cholesterol	Moderate
Korat et al, 2020, USA[20]	NCC	22.0	NHS	1,314	43-70	0.0	GLC	Erythrocyte membrane	Phospholipid fraction	20:0, 22:0, 24:0	Self-report	133 incident T2D	Age, smoking, alcohol drinking, BMI, physical activity, race, family history of diabetes, parental history of myocardial infarction, hypercholesterolemia, hypertension, menopausal status, postmenopausal hormone use, Alternative Healthy Eating Index, glycemic load, total energy	High
Korat et al, 2020, USA[20]	NCC	22.0	HPFS	1,517	47-81	100.0	GLC	Erythrocyte membrane	Phospholipid fraction	20:0, 22:0, 24:0	Self-report	112 incident T2D	Age, smoking, alcohol drinking, BMI, physical activity, race, family history of diabetes, parental history of myocardial infarction, hypercholesterolemia, hypertension, menopausal status, postmenopausal hormone use, Alternative Healthy Eating Index, glycemic load, total energy	High

Bragg et al, 2022, China[17]	PCC	8.0	CKB	1,671	30-79	NR	NMR	Plasma	Total fatty acid fraction	Total SFAs	Fasting glucose testing	882 incident T2D	Age, sex, education, smoking, alcohol drinking, BMI, physical activity, study area, fasting time, dietary factors, family history of diabetes, waist circumference	High
Zhuang et al, 2022, UK[44]	PC	11.6 (mean)	UK Biobank	95,854	37-73	44.5	NMR	Plasma	Total fatty acid fraction	Total SFAs	Records	3,052 incident T2D	Age, sex, education, smoking, alcohol drinking , physical activity, race, Townsend deprivation index, household income, history of hypertension, history of high cholesterol, family history of diabetes, vitamin supplement use, mineral supplement use, aspirin use, remaining plasma fatty acids	High
Warensjö et al, 2008, Sweden[45]	PC	30.7 (median)	ULSAM	2,009	≥50	100.0	GC	Serum	Cholesterol fraction	14:0, 16:0, 18:0	Records	461 CVD deaths	Smoking, BMI, physical activity, total cholesterol, hypertension	High
Woodward et al, 2011, Scotland[46]	PC	19.5 (median)	SHHECS	3,944	40-59	53.1	GC	Adipose tissue	-	Total SFAs, 14:0, 16:0, 18:0	Records	870 incident CVD	Age, sex, smoking, socio-economic status, SBP, BP treatment, diabetes, family history, blood lipid	High
Chien et al, 2013, China (Taiwan)[47]	PC	9.6 (median)	Cohort in Taiwan	1,833	≥35	55.5	GC	Plasma	Total fatty acid fraction	Total SFAs	Records	275 incident CVD	Age, sex, education, smoking, alcohol drinking, BMI, physical activity, occupation, marital status, hypertension, diabetes, blood lipid	Moderate
Fretts et al, 2016, USA[48]	PC	19.0	CHS	3,941	65-98	41.0	GC	Plasma	Phospholipid fraction	16:0, 18:0, 20:0, 22:0, 24:0	Records, interview	1,216 CVD deaths	Age, sex, education, smoking, alcohol drinking, BMI, physical activity, race, clinic site, waist circumference, hypertension, diabetes, CVD, cancer, health status	High
de Oliveira Otto et al, 2018, USA[21]	PC	22.0	CHS	2,907	≥65	36.0	GC	Plasma	Phospholipid fraction	15:0, 17:0	Records, interview	1,301 incident CVD	Age, sex, education, race, enrollment site, drug-treated hypertension, self-reported general health, circulating fatty acids, dietary factors	High
Trieu et al, 2021, Swedish[49]	PC	16.6 (median)	Stockholm Cohort	4,150	≥60	49.0	GC	Serum	Cholesterol fraction	15:0	Records	578 incident CVD	Age, sex, education, smoking, alcohol drinking, BMI, physical activity, prevalent hypertension, hyperlipidaemia, type 2 diabetes	Moderate
Simon et al, 1995, USA[50]	NCC	6.9 (mean)	MRFIT	188	35-57	100.0	GLC	Serum	Phospholipid fraction, cholesterol fraction	14:0, 16:0, 18:0	Records, interview	94 incident CHD	Age, alcohol drinking, clinic site, date of randomization from the MRFIT, blood lipid, serum fatty acids	Moderate

Wang et al, 2003, USA[51]	PC	10.7	ARIC	3,591	45-64	46.0	GLC	Plasma	Phospholipid fraction	Total SFAs, 16:0, 18:0	Records	282 incident CHD	Age, sex, smoking, alcohol drinking, physical activity, dietary factors	High
Warensjö et al, 2004, Sweden[52]	NCC	1.5	VIP MONICA	234	30-60	79.5	GLC	Serum	Phospholipid fraction	15:0, 17:0, 15:0+17:0	Records	78 incident MI	Age, sex, smoking, BMI, geographical region, SBP, DBP, blood lipidspecific insulin, insulin, pro-insulin, leptin, plasminogen activator inhibitor-1,tissue-type plasminogen activator and von Willebrandt factor	High
Sun et al, 2007, USA[53]	NCC	13.0	NHS	493	43-68	0.0	GLC	Erythrocyte membrane	Phospholipid fraction	15:0, 17:0	Records	166 incident IHD	Age, smoking, alcohol drinking, BMI, physical activity, fasting status, time of blood drawing, postmenopausal status, postmenopausal hormone use, aspirin, parental MI before age 65 y, hypertension, hypercholesterolemia, diabetes, plasma or erythrocytes fatty acids	High
Clarke et al, 2009, UK[15]	NCC	6.8 (mean)	Whitehall	355	40-69	100.0	GC	Plasma	Phospholipid fraction	Total SFAs	Records	116 CHD death	Age, smoking, BMI, civil service employment grade, diabetes, treatment for high BP, SBP, blood lipid, CRP, albumin, fibrinogen	High
Warensjö et al, 2010, Sweden[54]	NCC	3.6	NSHDS	1,000	30-60	61.5	GLC	Serum	Phospholipid fraction	15:0, 17:0, 15:0+17:0	Records	444 incident MI	Age, sex, education, smoking, BMI, physical activity, geographical region, diabetes, dietary factors, SBP, apo-ratio	High
Wu et al, 2011, USA[55]	PC	15.0	CHS	2,890	65-97	37.0	GC	Plasma	Phospholipid fraction	16:0	Records	631 incident CHD	Age, sex, education, smoking, alcohol drinking, BMI, physical activity, race, income, diabetes, hypertension history of stroke or transient ischemic attack, total fat and total energy intake	High
Khaw et al, 2012, UK[56]	NCC	13.0 (mean)	EPIC-Norfolk	7,354	40-79	52.2	GC	Plasma	Phospholipid fraction	14:0, 15:0, 16:0, 17:0, 18:0, 15:0+17:0	Records	2,424 incident CHD	Age, sex, education, smoking, alcohol drinking, BMI, physical activity, social class, diabetes, SBP, blood lipid, plasma vitamin C	High
Malik et al, 2015, USA[57]	NCC	13.0	NHS	762	30-55	0.0	GLC	Erythrocyte membrane	Phospholipid fraction	20:0, 22:0, 24:0	Records	348 incident CHD	Age, smoking, alcohol drinking, BMI, physical activity, fasting status time of blood draw, parental history of MI before age 65 years, menopausal status and hormone use, use of aspirin, AHEI score, history of hypercholesterolemia, diabetes, or hypertension, and plasma	High

													trans fatty acids and long-chain n-3 fatty acids	
Malik et al, 2015, USA[57]	NCC	13.0	HPFS	1,265	40-75	100.0	GLC	Erythrocyte membrane	Phospholipid fraction	20:0, 22:0, 24:0	Records	446 incident CHD	Age, smoking, alcohol drinking, BMI, physical activity, fasting status time of blood draw, parental history of MI before age 65 years, menopausal status and hormone use, use of aspirin, AHEI score, history of hypercholesterolemia, diabetes, or hypertension, and plasma trans fatty acids and long-chain n-3 fatty acids	High
Sun et al, 2016, Singapore[58]	NCC	11.5	SCHS	1,454	47-83	64.7	GC-MS/MS	Plasma	Total fatty acid fraction	16:0, 18:0	Records	744 incident MI	Age, sex, education, smoking, alcohol drinking, BMI, physical activity, hours of fasting before blood collection, hypertension, diabetes	Moderate
Chei et al, 2018, Japan[16]	NCC	11.0 (median)	CIRCS	608	40-85	61.1	GC	Serum	Total fatty acid fraction	Total SFAs, 14:0,16:0,18:0	Interview	152 incident CAD	Age, sex, smoking, alcohol drinking, BMI, community, year of serum stored, fasting status	High
Liu et al, 2019, USA[59]	NCC	4.5 (mean)	WHI-OS	2,428	50-79	0.0	GC	Plasma	Phospholipid fraction	Total SFAs	Records	1,214 incident CHD	Age, education, smoking, alcohol drinking, BMI, physical activity, race, enrollment date, hysterectomy status, region, income, family history of MI/diabetes/stroke, medication use, postmenopausal hormone use, self-reported hypertension/diabetes/hypercholesterolemia, percent calories from protein/carbohydrates, total energy intake	High
Trieu et al, 2021, Swedish[49]	PC	16.6 (median)	Stockholm Cohort	4,150	≥60	49.0	GC	Serum	Cholesterol fraction	15:0	Records	192 incident CHD	Age, sex, education, smoking, alcohol drinking, BMI, physical activity, prevalent hypertension, hyperlipidaemia, type 2 diabetes	Moderate
Iso et al, 2002, Kyowa[60]	NCC	9.0	Cardiovascular risk surveys in Kyowa	788	40-85	53.0	GC	Serum	Total fatty acid fraction	Total SFAs, 14:0, 16:0, 18:0	Records	197 incident stroke	Age, sex, smoking, alcohol drinking, BMI, hypertension, blood lipid, serum glucose category, serum fatty acids	High

Wiberg et al, 2006, Sweden[61]	PC	29.3 (median)	ULSAM	2,313	50	100.0	GC	Serum	cholesterol fraction	14:0, 16:0,18:0	Records	421 incident stroke or TIA	Smoking, physical activity, antihypertensive, antidiabetic, lipid-lowering drugs, hypertension, diabetes, atrial fibrillation, CVD, the metabolic syndrome, blood lipid	High
Warensjö et al, 2009, Sweden[62]	NCC	3.0	VIP	324	30-60	61.7	GLC	Plasma	Phospholipid fraction	15:0, 17:0	Records	108 incident stroke	Age, sex, smoking, alcohol drinking, BMI, physical activity, tobacco use, blood lipid, SBP, DBP, dietary factors	High
Yaemsiri et al, 2013, USA[63]	NCC	10.0	WHI-OS	1,928	50-79	0.0	GC	serum	Total fatty acid fraction	14:0, 15:0, 16:0, 17:0, 18:0	Self-report	964 incident ischemic stroke	Age, smoking, BMI, race, time on follow-up, diabetes, aspirin use, SBP, antihypertensive medication use, total cholesterol to HDL-C ratio and normalized-triglycerides	High
Yamagishi et al, 2013, USA[64]	PC	22.0	ARIC	3,870	45-64	48.0	GLC	Plasma	Phospholipid fraction, cholesterol fraction	Total SFAs, 14:0, 15:0, 16:0; 18:0	Records	168 incident ischemic stroke	Age, sex, smoking, alcohol drinking, cigarette-years	High
Yakoob et al, 2014, USA[65]	NCC	8.3 (median)	NHS	944	47-72	0.0	GLC	Erythrocyte membrane	Phospholipid fraction	14:0, 15:0, 17:0	Records	472 incident stroke	Age, smoking, alcohol drinking, BMI, physical activity, month of blood collection, race, family history of diabetes, menopausal status in the NHS, postmenopausal hormone use in NHS, consumption of dietary factors, glycemic load , dietary factors, plasma fatty acids	High
Yakoob et al, 2014, USA[65]	NCC	8.3 (median)	HPFS	244	43-78	100.0	GLC	Erythrocyte membrane	Phospholipid fraction	14:0, 15:0, 17:0	Records	122 incident stroke	Age, smoking, alcohol drinking, BMI, physical activity, month of blood collection, race, family history of diabetes, menopausal status in the NHS, postmenopausal hormone use in NHS, consumption of dietary factors, glycemic load , dietary factors, plasma fatty acids	High
Trieu et al, 2021, Swedish[49]	PC	16.6 (median)	Stockholm Cohort	4,150	≥60	49.0	GC	Serum	Cholesterol fraction	15:0	Records	386 incident stroke	Age, sex, education, smoking, alcohol drinking, BMI, physical activity, prevalent hypertension, hyperlipidaemia, type 2 diabetes	Moderate

Huang et al, 2021, USA[66]	PC	10.5 (median)	CHS	2,028	≥65	39.0	GC	Plasma	Phospholipid fraction	14:0, 15:0, 16:0, 18:0, 20:0, 22:0, 24:0	Self-report, records	338 incident stroke	Age, sex, education, smoking, BMI, physical activity, race, field center, all other NEFAs, serum albumin, alcohol drinking consumption, cystatin C for estimate glomerular filtration rate, aspirin use, waist circumference, hypertension, prevalent diabetes, total serum cholesterol concentration	High

AHEI, the alternate Healthy Eating Index; ARIC, Atherosclerosis Risk in Communities Study; BMI, body mass index; BP, blood pressure; CAD, Coronary Artery Disease; CHD, coronary heart disease; CHS, Cardiovascular Health Study; CIRCS, Circulatory Risk in Communities Study; CKB, the China Kadoorie Biobank; CRP, C-reactive protein; CVD, cardiovascular disease; DBP, diastolic blood pressure; DM, diabetes mellitus; EMFA, erythrocyte membrane fatty acid; EPIC-InterAct, European Prospective Investigation into Cancer and Nutrition (EPIC)–InterAct Study; EPIC-Norfolk, European Prospective Investigation into Cancer and Nutrition (EPIC)–Norfolk Study; EPIC-Postsdam, European Prospective Investigation into Cancer and Nutrition (EPIC)–Potsdam Study; GC, gas chromatography; GC-MS/MS, Gas chromatography-triple quadrupole mass spectrometry; GLC, gas-liquid chromatography; GNHS, the Guangzhou Nutrition and Health Study; HbA1c, hemoglobin A1c; HDL, high-density lipoprotein; HDL-C, high-density lipoprotein cholesterol; HHS, Hitachi Health Study; HPFS, Health Professionals Follow-Up Study; IHD, ischemic heart disease; KIHD, Kuopio Ischemic Heart Disease Risk Factor study; MCCS, Melbourne Collaborative Cohort Study; MI, myocardial infarction; MONICA, Monitoring of Trends and Cardiovascular Disease study; MRFIT, Multiple Risk Factor Intervention Trial; NCC, nested case-control study; NEFAs, nonesterified fatty acids; NHAPC, the Nutrition and Health of Aging Population in China study; NHS, Nurses' Health Study; NMR, nuclear magnetic resonance-based profiling; NR, not reported; NSHDS, the Scottish Heart Health Extended Cohort Study; PC, prospective cohort study; PCC, prospective case-cohort study; SBP, systolic blood pressure; SCHS, Singapore Chinese Health Study; SFAs, saturated fatty acids; SHHECS, the Scottish Heart Health Extended Cohort Study; T2D, type 2 diabetes; TIA, transient ischemic attack; UK, United Kingdom; ULSAM, Uppsala Longitudinal Study of Adult Men; USA, the United States of America; VIP, Västerbotten Intervention Programme; WHIMS, Women's Health Initiative Memory Study; WHI-OS, Women's Health Initiative Observational Study; WHR, waist hip rate.

[*] Study quality was assessed with the Newcastle-Ottawa Scale.

Table S2. Newcastle-Ottawa Scale assessments for prospective studies on saturated fatty acid biomarkers and cardiometabolic diseases

First author, year	Selection	Comparability	Assessment of exposure	Total Score	Quality*
Laaksonen et al, 2002[27]	4	2	2	8	High
Wang et al, 2003[28]	4	2	3	9	High
Hodge et al, 2007[29]	4	2	1	7	High
Krachler et al, 2008[30]	4	0	3	7	High
Mozaffiarian et al, 2010[31]	4	2	3	9	High
Patel et al, 2010[32]	4	2	3	9	High
Kröger et al, 20113[33]	4	2	3	9	High
Mozaffarian et al, 2013[34]	3	2	3	8	High
Zong et al, 2013[35]	4	2	2	8	High
Forouhi et al, 2014[36]	4	2	3	9	High
Lemaitre et al, 2015[37]	3	2	3	8	High
Ma et al, 2015[38]	3	2	3	8	High
Harris et al, 2016[39]	3	2	2	7	High
Yakoob et al, 2016 (NHS)[40]	3	2	3	8	High
Yakoob et al, 2016 (HPFS)[40]	3	2	3	8	High
Akter et al, 2017[41]	4	2	3	9	High
Lin et al, 2018[42]	3	2	3	8	High
Lu et al, 2018[43]	3	0	3	6	Moderate
Korat et al, 2020 (NHS)[20]	3	2	3	8	High
Korat et al, 2020 (HPFS)[20]	3	2	3	8	High
Bragg et al, 2022[17]	4	2	3	9	High
Zhuang et al, 2022[44]	4	2	3	9	High
Warensjö et al, 2008[45]	4	2	3	9	High
Woodward et al, 2011[46]	3	2	2	7	High
Chien et al, 2013[47]	2	2	2	6	Moderate
Fretts et al, 2016[48]	3	2	3	8	High
de Oliveira Otto et al, 2018[21]	3	2	3	8	High
Trieu et al, 2021[49]	4	0	2	6	Moderate
Simon et al, 1995[50]	2	0	3	5	Moderate
Wang et al, 2003[51]	4	2	3	9	High
Warensjö et al, 2004[52]	4	0	3	7	High
Sun et al, 2007[53]	3	2	3	8	High
Clarke et al, 2009[15]	3	2	3	8	High
Warensjö et al, 2010[54]	3	2	3	8	High
Wu et al, 2011[55]	4	2	2	8	High
Khaw et al, 2012[56]	4	2	3	9	High
Malik et al, 2015 (NHS)[57]	3	2	3	8	High
Malik et al, 2015 (HPFS)[57]	3	2	3	8	High
Sun et al, 2016[58]	3	0	3	6	Moderate
Chei et al, 2018[16]	3	2	3	8	High
Liu et al, 2019[59]	3	2	2	7	High
Iso et al, 2002[60]	3	2	3	8	High

Wiberg et al, 2006[61]	4	2	3	9	High
Warensjö et al, 2009[62]	4	0	3	7	High
Yaemsiri et al, 2013[63]	3	2	2	7	High
Yamagishi et al, 2013[64]	4	2	3	9	High
Yakoob et al, 2014 (NHS)[65]	3	2	3	8	High
Yakoob et al, 2014 (HPFS)[65]	3	2	3	8	High
Huang et al, 2021[66]	3	3	3	9	High

*Study quality was assessed based on the nine-star Newcastle–Ottawa Scale (NOS) using pre-defined criteria namely: selection (population representativeness), comparability (adjustment for confounders), and ascertainment of outcome. Studies were defined as low, moderate, and high quality for those scored 0-3, 4-6, and 7-9, respectively (9 reflecting the highest quality)
